# A coordinated multiorgan metabolic response contributes to human mitochondrial myopathy

**DOI:** 10.15252/emmm.202216951

**Published:** 2023-05-24

**Authors:** Nneka Southwell, Guido Primiano, Viraj Nadkarni, Nabeel Attarwala, Emelie Beattie, Dawson Miller, Sumaitaah Alam, Irene Liparulo, Yevgeniya I Shurubor, Maria Lucia Valentino, Valerio Carelli, Serenella Servidei, Steven S Gross, Giovanni Manfredi, Qiuying Chen, Marilena D'Aurelio

**Affiliations:** ^1^ Brain and Mind Research Institute Weill Cornell Medicine New York NY USA; ^2^ Fondazione Policlinico Universitario Agostino Gemelli IRCCS Rome Italy; ^3^ Dipartimento di Neuroscienze Università Cattolica del Sacro Cuore Rome Italy; ^4^ Department of Pharmacology Weill Cornell Medicine New York NY USA; ^5^ IRCCS, Institute of Neurological Sciences of Bologna, Bellaria Hospital Bologna Italy; ^6^ Department of Biomedical and NeuroMotor Sciences (DIBINEM) University of Bologna Bologna Italy

**Keywords:** amino acid metabolism, glucocorticoids, leptin, mitochondrial myopathy, muscle wasting, Metabolism, Musculoskeletal System, Organelles

## Abstract

Mitochondrial diseases are a heterogeneous group of monogenic disorders that result from impaired oxidative phosphorylation (OXPHOS). As neuromuscular tissues are highly energy‐dependent, mitochondrial diseases often affect skeletal muscle. Although genetic and bioenergetic causes of OXPHOS impairment in human mitochondrial myopathies are well established, there is a limited understanding of metabolic drivers of muscle degeneration. This knowledge gap contributes to the lack of effective treatments for these disorders. Here, we discovered fundamental muscle metabolic remodeling mechanisms shared by mitochondrial disease patients and a mouse model of mitochondrial myopathy. This metabolic remodeling is triggered by a starvation‐like response that evokes accelerated oxidation of amino acids through a truncated Krebs cycle. While initially adaptive, this response evolves in an integrated multiorgan catabolic signaling, lipid store mobilization, and intramuscular lipid accumulation. We show that this multiorgan feed‐forward metabolic response involves leptin and glucocorticoid signaling. This study elucidates systemic metabolic dyshomeostasis mechanisms that underlie human mitochondrial myopathies and identifies potential new targets for metabolic intervention.

## Graphical abstract

In patients affected by OXPHOS defects and a mouse model of mitochondrial myopathy, metabolic adaptations initiated by mitochondrial integrated stress response (ISRmt) are part of inter‐organ crosstalk coordinated by myokines and hormonal signaling.

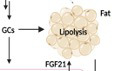

**The paper explained**

**Problem**

Mitochondrial diseases are genetic disorders caused by the impairment of the oxidative phosphorylation (OXPHOS) metabolism, affecting tissues that are heavily energy dependent, and often manifesting with neuromuscular symptoms. Although the energetic defects arising from genetic errors in mitochondrial and nuclear DNA are often known, several aspects of mitochondrial disease pathogenesis are yet to be elucidated. Because of the lack of defined metabolic targets, no proven effective treatments are available for these diseases.

**Results**

In patients affected by OXPHOS defects and a mouse model of mitochondrial myopathy, we find that a starvation‐like response promotes muscle protein breakdown and amino acid utilization to support compensatory energy‐generating mechanism. Diseased muscle communicates with adipose tissue and liver, modulating leptin levels to stimulate the hypothalamic–pituitary–adrenal axis causing increased glucocorticoid levels. This promotes fatty acids release from the adipose tissue. Since muscle is unable to utilize lipids for energy purposes, this leads to potentially toxic intramuscular lipid accumulation.

**Impact**

These findings suggest that metabolic rewiring underlies maladaptive effects in mitochondrial myopathies, and that energy substrate supplementation to prevent endogenous amino acid consumption in combination with inhibition of glucocorticoid signal could be beneficial to treat these diseases.

## Introduction

Myopathy is one of the most common clinical manifestations of mitochondrial diseases (Berardo *et al*, 2010; Ahmed *et al*, 2018; Ng *et al*, 2021). Genetically defined disorders which lead to OXPHOS defects and predominantly affect skeletal muscle are defined as primary mitochondrial myopathies (PMM; Mancuso *et al*, 2017). Symptoms of PMM can range from relatively non‐specific exercise intolerance or exercise‐induced symptoms, to muscle weakness and wasting. No proven effective treatments or cures are available for PMM (Gorman *et al*, 2016) and clinical trials lack consensus on metabolic outcome measures (Mancuso *et al*, 2017). This is largely due to a limited understanding of the metabolic consequences of OXPHOS deficiency in skeletal muscle.

Metabolite dyshomeostasis has been extensively reported in mitochondrial patients. Notably, increased levels of lactic acid and tricarboxylic acid (TCA) cycle intermediates are frequently found in the blood, urine, cerebrospinal fluid, and tissues of patients with OXPHOS deficiencies (Smeitink *et al*, 2006). In these patients, metabolic acidosis often results in irreversible coma and eventual death (Danhauser *et al*, 2015). Amino‐ and fatty acid metabolism is also severely affected in mitochondrial diseases. For example, elevated plasma alanine is considered a biomarker of mitochondrial disease (Morava *et al*, 2006; Shatla *et al*, 2014; Maresca *et al*, 2020). Together, a combination of elevated plasma alanine, glycine, proline, sarcosine, tyrosine, and acyl‐carnitines, accompanied by low levels of free carnitine, supports a diagnosis of mitochondrial disease (Mitochondrial Medicine Society's Committee on Diagnosis *et al*, 2008). Decreased levels of plasma and cerebrospinal fluid folate, cofactor of the serine one‐carbon metabolism, are detected in Kearns–Sayre syndrome patients (Allen *et al*, 1983; Ramaekers & Quadros, 2022). Additionally, alterations of plasma citrulline and arginine have been reported in NARP/MILS (Neuropathy, ataxia, and retinitis pigmentosa/Maternally inherited Leigh syndrome) (Rabier *et al*, 1998; Parfait *et al*, 1999; Debray *et al*, 2010) and MELAS (mitochondrial encephalomyopathy, lactic acidosis, and stroke‐like episodes) (Naini *et al*, 2005; Koga *et al*, 2007) patients. In MELAS, citrulline and arginine supplementation has been proposed as a therapeutic approach to increase nitric oxide synthesis for improved blood perfusion in the brain (El‐Hattab *et al*, 2016, 2017) and stroke‐like symptoms (Koga *et al*, 2005, 2007). However, despite this evidence of altered amino‐ and fatty acid metabolism underlying the systemic organic acid dyshomeostasis of mitochondrial patients, the biochemical mechanisms and pathogenic significance of these alterations remain incompletely defined.

**Figure 1:**
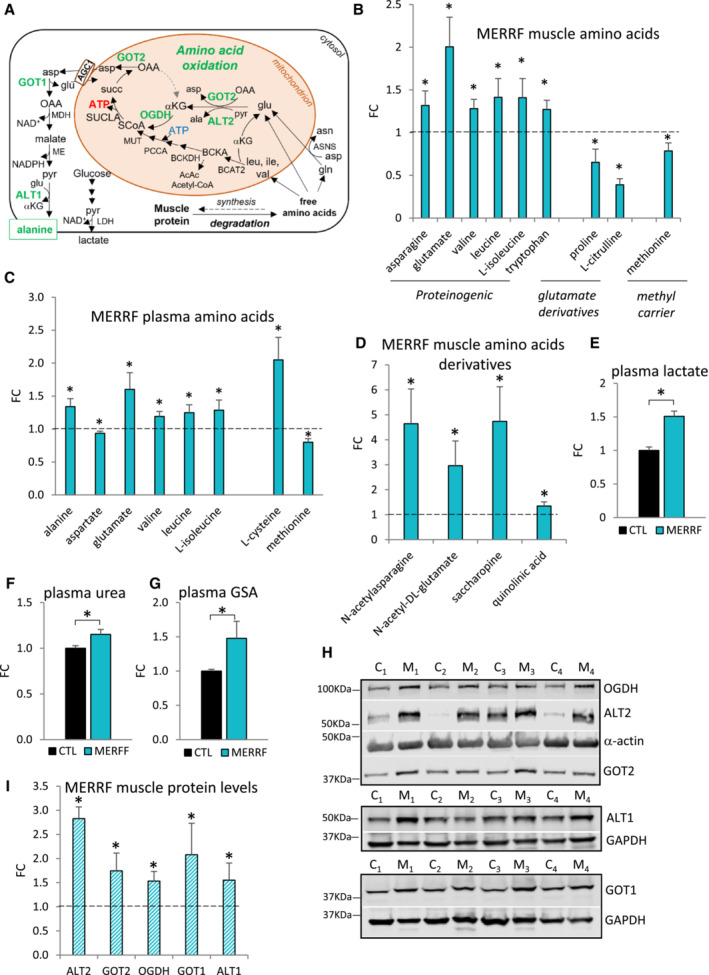
Glutamate oxidation through the TCA cycle is upregulated in MERRF muscle A
Schematic representation of amino acid oxidation in OXPHOS‐defective MERRF muscle. Enzymes of amino acid utilization in green are upregulated in MERRF, as detected by western blot analysis. αKG, α‐ketoglutarate; ala, alanine; asn, asparagine; asp, aspartate; BCKA, branched chain keto acids; glu, glutamate; gln, glutamine; ile, isoleucine; leu, leucine; val, valine; OAA, oxaloacetate; pyr, pyruvate; succ, succinate; SCoA, succinyl‐CoA.B, C
Levels of differential free amino acids in MERRF patients' muscle (B) and plasma (C) by LC–MS analysis, expressed relative to CTL.D
Levels of amino acid derivatives in MERRF muscle by LC–MS analysis, expressed relative to CTL.E–G
Plasma levels of lactate (E), urea (F), and guanidinosuccinic acid (GSA) (G) by LC–MS analysis, expressed relative to CTL value set at 1.H
Western blots of muscle lysates separated by denaturing SDS–PAGE and probed for OGDH, ALT2, α‐actin, and GOT2 (top panel), ALT1 and GAPDH (middle panel), GOT1 and GAPDH (bottom panel), C (CTL), M (MERRF).I
Protein levels in MERRF muscle expressed relative to CTL muscle estimated by band densitometry normalized by α‐actin (OGDH, ALT2, GOT2) and by GAPDH (ALT1, GOT1). Data information: In panels (B–D, and I), the dashed line indicates the Mean CTL value set at 1. In panels (B–G), data are presented as Mean ± SEM. Muscle: MERRF (*n* = 10), CTL (*n* = 15); Plasma: MERRF (*n* = 9), CTR (*n* = 25). In panel (I), data are presented as Mean ± SD. MERRF muscle (*n* = 4), CTL muscle (*n* = 4). Statistically significant differences between the two groups were estimated by unpaired two‐tailed Student's test. *P < 0.05 MERRF versus CTL. Source data are available online for this figure.

The TCA cycle is the hub of energy metabolism, oxidizing nutrients to CO_2_ while producing reducing equivalents (NADH and FADH_2_), which can be oxidized by the OXPHOS system for ATP generation. The main TCA input fuel is acetyl‐CoA, derived from both glycolysis‐derived pyruvate and fatty acid oxidation. However, alternative TCA input sources derive from the carbon skeletons of deaminated amino acids that enter the TCA cycle as α‐ketoglutarate (αKG, derived from glutamate and glutamine), succinyl‐CoA (derived from valine and isoleucine), and acetyl‐CoA (derived from isoleucine and leucine), via a process termed anaplerosis. A common assumption is that a defective OXPHOS system with a restricted capacity for oxidization would limit oxidative decarboxylation flux through the TCA cycle to prevent the accumulation of reducing equivalents (NADH and FADH_2_). Nevertheless, we have demonstrated that in contrast to this common view, an accelerated glutamine‐driven αKG anaplerosis serves as a compensatory energy source for substrate‐level ATP production via the TCA enzyme succinate‐CoA ligase (SUCLA, Fig 1A) in OXPHOS‐defective mtDNA mutant cells (Chen *et al*, 2018). In these cells, accelerated glutamine anaplerosis leads to a cataplerotic efflux of aspartate from mitochondria, with subsequent cytosolic conversion to alanine and lactate and their secretion into the extracellular medium. This strategic metabolic rewiring provides for regeneration of both NAD^+^ and NADPH, while limiting potentially toxic ammonia accumulation (Fig 1A). Importantly, this amino acid oxidation mechanism extends to the *in vivo* setting, in a mouse model of mitochondrial myopathy (Chen *et al*, 2018).

In this study, we define the metabolic responses to OXPHOS defect in human muscle from m.8344A>G MERRF patients affected by severe mitochondrial myopathy. Moreover, we detect similar metabolic responses in a murine model of mitochondrial myopathy. We show that in OXPHOS defective muscle there is an early metabolic shift toward amino acid utilization for energy generation and activation of alternative pathways of mitochondrial NADH oxidation. As disease progresses in the animals, this muscle metabolic rewiring triggers a multiorgan response comprising accelerated liver gluconeogenesis, ureagenesis, and ketogenesis, as well as glucocorticoid‐mediated activation of systemic catabolic signaling. We propose that these previously unappreciated metabolic responses are fundamental effectors of muscle and fat store wasting that are defining features of mitochondrial myopathies.

## Results

### Amino acid catabolism is increased in MERRF skeletal muscle

The metabolic response to OXPHOS defect was studied in the muscle and plasma of MERRF patients with severe myopathy. The cohort of patients used in this study was homogeneous both genetically (m.8344A>G mutation in the *MT‐TK* gene encoding the tRNA^Lys^, heteroplasmy level in muscle 80% ± 6) and clinically (Appendix Table S1). Age‐ and sex‐matched controls (CTL) were diagnosed as disease‐free (Appendix Table S1). LC–MS analysis (Appendix Fig S1A–D) revealed defined MERRF patients' muscle and plasma free amino acid profiles (Fig 1B and C). Notably, leucine, isoleucine, valine (branched chain amino acids, BCAA), glutamate, asparagine, and tryptophan were elevated in muscle (Fig 1B) and BCAA, glutamate, and alanine were increased in plasma (Fig 1C). Free BCAA and glutamate, derived from the diet or released from protein breakdown, are among the few amino acids that can be oxidized in skeletal muscle through the TCA cycle (Wagenmakers, 1998a; Wagenmakers, 1998b, Fig 1A). Moreover, asparagine and glutamate are generated by transamination of glutamine to aspartate (by asparagine synthetase, ASNS, Fig 1A). Increased levels of asparagine, glutamate, and their acetylated derivatives (N‐acetyl asparagine and N‐acetyl glutamate, Fig 1D) suggest an attempt to oxidize amino acids for energy generation. Interestingly, N‐acetyl asparagine is a product of asparagine degradation which is excreted in the urine and N‐acetyl glutamate is the allosteric activator of mitochondrial carbamoyl phosphate synthetase I (CPSI), the first enzyme in the urea cycle. Therefore, an increase in these metabolites suggests the activation of pathways of ammonia disposal in MERRF muscle. Furthermore, we also detected increased levels of saccharopine and quinolinic acid (Fig 1D), degradation intermediates of the catabolism of lysine and tryptophan, respectively. Taken together, these results suggest that MERRF muscle is characterized by increased amino acid catabolism.

### MERRF muscle oxidation of glutamate through a truncated TCA cycle results in increased alanine synthesis and release in the circulation

We found elevated levels of lactate (Fig 1E) and alanine (Fig 1C) in MERRF plasma. In muscle, while lactate is produced by oxidoreductases, alanine is synthesized by the cytosolic (ALT1) and mitochondrial (ALT2) aminotransferases which transfer the amino group of glutamate to pyruvate co‐generating αKG. Since αKG is oxidized through the TCA cycle (Fig 1A), alanine produced in muscle as a byproduct of glutamate oxidation is released in the circulation. Therefore, increased plasma alanine levels in MERRF patients likely reflect increased muscle glutamate oxidation. After being released in circulation, alanine becomes available for liver gluconeogenesis and ureagenesis as a mechanism to limit ammonia accumulation in muscle (glucose‐alanine Cahill cycle; Wagenmakers, 1998a). Therefore, increased levels of plasma urea in MERRF patients (Fig 1F) reflect increased alanine release from muscle and liver disposal. Accumulation of urea in plasma can also activate alternative pathways of liver ammonia disposal, which results in the formation of guanidinosuccinic acid (GSA) (Cohen *et al*, 1968; Noris & Remuzzi, 1999). Elevated levels of plasma GSA in MERRF patients (Fig 1G) therefore reflect enhanced hepatic ureagenesis linked to increased muscle amino acid oxidation.

To further explore the molecular underpinnings of increased glutamate oxidation, we analyzed protein levels of key enzymes of glutamate anaplerosis and cataplerosis in MERRF muscle (Fig 1H and I). We found elevated levels of the two enzymes catalyzing mitochondrial conversion of glutamate to αKG, alanine aminotransferase (ALT2, 180% increase) and aspartate aminotransferase (GOT2, 70% increase). Together with increased levels of α‐ketoglutarate dehydrogenase (OGDH, 50% increase) these results are indicative of glutamate–derived αKG oxidation through the TCA cycle. Finally, upregulation of two key enzymes of the cytosolic alanine production, aspartate aminotransferase (GOT1, 100% increase) and alanine aminotransferase (ALT1, 60% increase) (Fig 1H and I), further suggests that amino acid oxidation is associated with cataplerotic efflux of aspartate from mitochondria, followed by cytosolic conversion of aspartate to alanine. Notably, the conversion of aspartate to alanine and to asparagine (Fig 1B) and acetyl‐asparagine (Fig 1D), could facilitate the glu to αKG oxidative influx in the TCA cycle by preventing accumulation of OAA, a potent inhibitor of succinate dehydrogenase.

### Increased glutamate oxidation parallels the progression of mitochondrial myopathy in COX10 KO mouse

We hypothesized that in OXPHOS defective muscle, a chronic metabolic shift toward amino acid utilization as an energy source contributes to tissue wasting. To test this, we assessed the longitudinal effects of OXPHOS deficiency on skeletal muscle amino acid metabolism in a genetic mouse model of muscle‐specific cytochrome c oxidase (COX, complex IV) deficiency (Diaz *et al*, 2005). In this mouse, genetic excision of the assembly factor heme A:farnesyltransferase (COX10) occurs selectively in skeletal muscle at birth, resulting in muscle COX deficiency throughout the mouse lifespan, confirmed by stable depletion of COX subunits encoded by mtDNA and by nuclear DNA (COXI and COX4, respectively, Appendix Fig S2A–D) and complete loss of fully assembled COX (by BN‐PAGE, Appendix Fig S2E). The COX10 KO mouse develops a progressive myopathy, which is similar to the muscle disease observed in m.8344A>G MERRF patients with COX‐negative muscle fibers (Appendix Fig S1E, La Morgia *et al*, 2020). In the COX10 KO mouse, exercise intolerance is detected by treadmill as early as 40 days of age (Appendix Fig S2F), which is followed by muscle weakness detected by grip strength (Appendix Fig S2G) and by weight loss. Weight loss is associated with decreased visceral fat deposits and muscle mass (Appendix Fig S2H and I) and commences at approximately 100 days of age (Appendix Fig S2J). Notably, the onset of muscle weakness is detected slightly earlier in COX10 KO females than males (at 50 days vs. 65 days, respectively, Appendix Fig S2G), but progresses similarly in both sexes. A previous report (Diaz *et al*, 2005) observed earlier mortality in COX10 KO females versus males (50% at 120 days in females vs. 210 days in males). However, we did not detect spontaneous mortality in either sex for our COX10 KO colony up to 200 days of age, when the mice were euthanized as a humane experimental endpoint. Both survival and sex differences noted above may be explained by a difference in genetic background, as our colony was comprised of pure C57Bl/6 mice, while 129svj and C57Bl/6 mice were cross bred in the previous study (Diaz *et al*, 2005). Nevertheless, to avoid potential confounding factors related to sex, our studies were performed in male mice only.

First, we assessed protein markers in COX10 KO muscle during disease progression at 50 days (early stage of myopathy), 100 days (the age of weight loss onset, intermediate stage of myopathy) and ≥ 170 days (late stage of myopathy). Levels of the inner mitochondrial membrane (IMM) protein TIM23 were found to be increased at 100 and 170 days compared to age‐matched control littermates (90 and 180% increase, respectively, Appendix Fig S2K and L), levels of the 70 kDa subunit of complex II were increased at 170 days (53% increase, Appendix Fig S2M and N), the matrix enzyme citrate synthase was increased at 100 and 170 days (38 and 30% increase, respectively, Appendix Fig S2O and P) and the outer mitochondrial membrane protein VDAC was increased at 170 days (30% increase, Appendix Fig S2Q and R). The variability in the upregulation of mitochondrial proteins (from 30 to 180%) at 200 days, shows that some pathways are more upregulated than others (e.g., TCA cycle and mitochondrial import), precluding their use as mitochondrial markers. Therefore, to compare muscle protein levels across muscle samples, we normalized protein levels to cytosolic GAPDH which was found to be stable in both CTL and COX10 KO muscles throughout the lifespan (Appendix Fig S2S and T).

**Figure 2:**
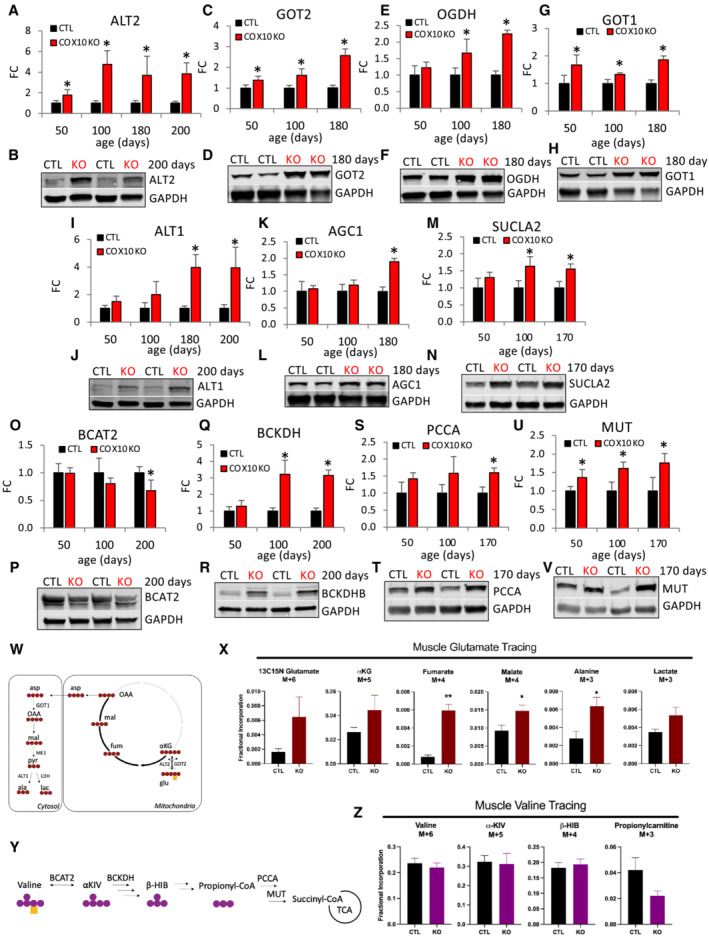
Upregulation of key components of amino acid oxidation progresses with age in COX10 KO muscle A–V
Age‐dependent muscle protein levels of ALT2 (A), GOT2 (C), OGDH (E), GOT1 (G), ALT1 (I), AGC1 (K), SUCLA2 (M), BCAT2 (O), BCKDH (Q), PCCA (S), and MUT (U), estimated by band densitometry normalized by GAPDH, in COX10 KO expressed relative to same age CTL set at 1. Representative western blots of muscle lysates from 170‐ to 200‐day‐old mice separated by denaturing SDS–PAGE and probed for ALT2 (B), GOT2 (D), OGDH (F), GOT1 (H), ALT1 (J), AGC1 (L), SUCLA2 (N), BCAT2 (P), BCKDHB (R), PCCA (T), and MUT (V) and GAPDH (B, D, F, H, J, L, N, P, R, T, V).W
Schematic representation of oxidative glutamate‐αKG flux. Red circles represent ^13^C atoms and yellow square represents ^15^N atom derived from [^13^C5, ^15^N]‐glutamate. αKG, α‐ketoglutarate; OAA, oxaloacetate.X
Muscle fractional incorporation of M + 6 of glutamate, M + 5 of αKG, M + 4 of fumarate, M + 4 of malate, M + 3 of alanine, and M + 3 of lactate, in 200 days COX10 KO and CTL mice IP injected with [^13^C5, ^15^N]‐glutamate.Y
Schematic representation of valine oxidative pathway. Purple circles represent ^13^C atoms and yellow square represent ^15^N atom derived from [^13^C5, ^15^N]‐valine. α‐KIV, α‐ketoisovalerate; β‐HIB, β‐hydroxyisobutyrate.Z
Muscle fractional incorporation of M + 6 of valine, M + 5 of α‐KIV, M + 4 of β‐ΗΙΒ, M + 3 of propionyl carnitine, in 200 days COX10 KO and CTL mice IP injected with [^13^C5, ^15^N]‐valine. Data information: In panels (A, C, E, G, I, K, M, O, Q, S, and U), data are presented as Mean ± SD. COX10 KO (*n* = 4), CTL (*n* = 4). In panels (X and Z), data are presented as Mean ± SEM. In panel (X), COX10 KO (*n* = 4), CTL (*n* = 4). In panel (Z), COX10 KO (*n* = 3), CTL (*n* = 3). Statistically significant differences between the two groups were estimated by unpaired two‐tailed Student's test. *P < 0.05, **P < 0.005 COX10 KO versus CTL. Source data are available online for this figure.

Next, we investigated the levels of key enzymes of amino acid oxidation focusing on glutamate oxidation, which is significantly altered in MERRF muscle (Fig 1). Levels of both ALT2 and GOT2 were increased as early as 50 days of age in COX10 KO versus CTL muscle (80 and 40%, respectively) and upregulation of these enzymes increased progressively with age (380 and 60% at 100 days, 280 and 160% at late stage for ALT2 and GOT2, respectively, Fig 2A–D). Levels of OGDH were elevated at 100 and 180 days, (70 and 130%, respectively, Fig 2E and F). Levels of cytosolic GOT1, which converts aspartate to OAA, were increased as early as 50 days (70% increase) and remained upregulated (33 and 90% increase at 100 and 180 days, respectively, Fig 2G and H). Finally, ALT1 and the aspartate–glutamate carrier (AGC) responsible for aspartate efflux from mitochondria and glutamate influx (Fig 1A) were increased only at late‐stage (300% for ALT1, Fig 2I and J, and 90% increase for AGC1, Fig 2K and L). When the levels of these enzymes were analyzed longitudinally within the COX10 KO group, a progressive increase in levels was paralleled by an apparent worsening of the myopathy (Appendix Fig S2U–Z). Taken together, these results indicate that upregulation of key enzymes involved in glutamate oxidation begins early and progresses with age.

Finally, the TCA enzyme SUCLA2, which catalyzes the conversion of succinyl‐CoA to succinate coupled with ADP phosphorylation/ATP generation (Fig 1A), was upregulated at 100 and 170 days of age (64 and 60%, respectively, Fig 2M and N), suggesting that glutamate can serve as important fuel sources for oxidative energy metabolism in COX10 KO muscle.

Since BCAA can also serve as an anaplerotic succinyl CoA source (Fig 1A) and elevated muscle levels of BCAA are observed in MERRF (Fig 1B), we investigated the age‐dependent regulation of key steps of BCAA utilization. In the first step, the amino group of BCAA is transferred to αKG by the mitochondrial BCAA aminotransferase (BCAT2), generating branched chain keto acids (BCKA) and glutamate (Fig 1A). After oxidation of BCKA by the BCKA dehydrogenase complex (BCKDH), the catabolic pathways of isoleucine and valine converge on the production of propionyl‐CoA, which is subsequently converted to methyl malonyl‐CoA (by propionyl‐CoA carboxylase, PCCA) through a reaction coupled with ATP hydrolysis. Methyl malonyl‐CoA is then converted to succinyl‐CoA (by methyl malonyl‐CoA mutase, MUT). BCAT2 levels were significantly decreased in COX10 KO muscle at 200 days (30% decrease, Fig 2O and P) and levels of BCKDHB (E1 subunit β of BCKDH) were increased at 100 and 200 days (200% increase, Fig 2Q and R). In addition, PCCA levels were increased at 170 days (60% increase, Fig 2S and T) and MUT upregulation occurred at earlier stages and progressed with age (40, 60, and 80% increase at 50, 100, and 170 days, respectively, Fig 2U and V). A decrease in BCAT2‐mediated transaminase would limit BCKA availability and oxidation despite upregulated downstream steps of BCAA catabolism. Of note, the removal of αKG by BCAT2‐mediated transamination would impair the energy‐generating glutamate anaplerosis. Therefore, downregulation of BCAT2 in muscle would ensure a continuous oxidative flux of glutamate and limit BCAA oxidation which is not net energy‐generating due to the ATP‐dependent PCCA reaction.

Together, these results indicate that preferred glutamate oxidation through the TCA circuit constitutes an early energetic response to OXPHOS deficiency in COX10 KO muscle. This energy‐generating compensatory mechanism would be driven by increased protein catabolism and progressive upregulation of glutamate utilization.

To confirm that glutamate oxidation through the TCA cycle is increased in COX10 KO muscle, we performed MS‐based *in vivo* stable isotope tracing study of labeled glutamate, via intraperitoneal injections (IP) in 200‐day‐old mice. In this tracer experiment, oxidative metabolism of [^13^C5, ^15^N]‐glutamate (M + 6) would predictably generate M + 5 form of αKG and M + 4 forms of succinate, malate, aspartate, owing to the incorporation of five or four ^13^C atoms in each of these species (Fig 2W). Moreover, the M + 4 form of aspartate, resulting from glu‐αKG oxidative flux, predictably generates M + 3 forms of alanine and lactate (Fig 2W). Isotopologue analysis of ^13^C enrichment in TCA cycle intermediates showed that in COX10 KO muscle, fumarate and malate have a significantly greater M + 4 fractional incorporation (Fig 2X), along with increased ^13^C enrichment and abundance (i.e., the combination of all labeled and unlabeled isotopologues, Appendix Fig S2Aa). Our tracing studies also revealed a significant enrichment in the M + 3 form of alanine (Fig 2X) confirming accelerated glutamate utilization and alanine production in COX10 KO muscle. Transamination of [^13^C5, ^15^N]‐glutamate (by GOT and ALT) generates ^15^N forms of aspartate and alanine (Appendix Fig S2Bb), which were detected in both muscle and plasma (Appendix Fig S2Cc). In accord with increased alanine synthesis and secretion, we found elevated levels of plasma ^15^N alanine in COX10 KO (Appendix Fig S2Cc). These results indicate that elevated plasma alanine levels reflect increased glutamate oxidation.

We also investigated muscle BCAA oxidation, by performing LC–MS‐based *in vivo* stable isotope tracing study of [^13^C5, ^15^N]‐valine, IP injected in 200‐day‐old mice. In this experiment, oxidative metabolism of [^13^C5, ^15^N]‐valine would predictably generate M + 5 form of α‐ketoisovalerate (α‐KIV), M + 4 form of β‐hydroxyisobutyrate (β‐HIB), and M + 3 form of propionyl CoA owing to the incorporation of five, four, or three ^13^C atoms in each of these species, respectively (Fig 2Y). No difference was found in the fractional incorporation of the α‐KIV, β‐HIB, and propionyl (carnitine) isotopologues (Fig 2Z), confirming that BCAA oxidation is not increased in COX10 KO muscle. We also found decreased α‐KIV abundance (Appendix Fig S2Dd), possibly reflecting limited BCAT levels (Fig 2O), and increased abundance of β‐HIB (Appendix Fig S2Dd), possibly reflecting reduced downstream oxidation.

Overall, these metabolite tracing results indicate that glutamate, but not BCAA, serves as an important alternative fuel source for oxidative energy metabolism in COX10 KO muscle.

### Upregulation of the glutamate flux through the TCA cycle is adaptive in COX10 KO muscle

The role of glutamate oxidation through the TCA cycle in COX10 KO muscle was further investigated by inhibiting glutamate conversion to α‐KG with aminooxy acetate (AOA). *In vivo* treatments with AOA, an inhibitor of PLP‐dependent transaminases (e.g., ALT, GOT), is effective in reducing cancer growth through the inhibition of the glutamine‐glutamate utilization pathway (Qing *et al*, 2012; Korangath *et al*, 2015). Therefore, we tested the effect of AOA administration in COX10 KO mice following the experimental protocols reported previously (Qing *et al*, 2012). Briefly, 70‐day‐old COX10 KO and CTL mice were intraperitoneally injected daily with 10 mg/Kg of AOA (treated) or PBS (untreated). After 7 weeks of treatment, exercise endurance (measured by treadmill) was decreased in COX10 KO mice treated with AOA versus COX10 KO untreated (Appendix Fig S2Ee), whereas no effect was observed in CTL mice (Appendix Fig S2Ff). Moreover, AOA had no effect on the body weight of COX10 KO and CTL mice (Appendix Fig S2Gg), suggesting that COX10 KO weight loss (Appendix Fig S2J) is independent of glutamate transamination.

To validate the *in vivo* inhibition of transaminases by AOA, we performed tracing studies with [^13^C5, ^15^N]‐glutamate at the end of the AOA treatment (130 days of age). We found decreased incorporation of M + 3 (^13^C) and M + 1 (^15^N) forms of alanine in muscle and plasma of mice treated with AOA (Appendix Fig S2Hh and Ii) and decreased muscle and plasma alanine (incorporated plus non‐incorporated) in COX10 KO AOA versus COX10 KO PBS (although not reaching statistical significance in plasma, Appendix Fig S2Hh), which confirmed inhibition of conversion of glutamate to alanine. In summary, our results suggest that upregulated oxidative glutamate flux in the TCA cycle is a beneficial metabolic adaptation of COX10 KO muscle.

### mTORC1 mediates inhibition of protein translation in COX10 KO muscle

**Figure 3:**
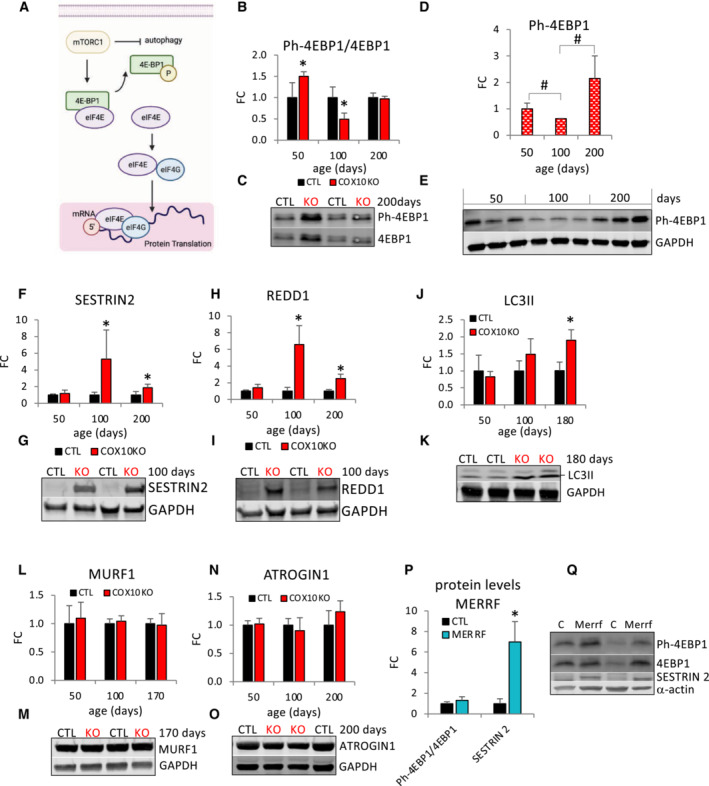
4E‐BP1 phosphorylation is decreased at intermediate stage and autophagy is elevated at late stage in COX10 KO muscle A
Schematic representation of mTORC1‐mediated regulation of protein translation through phosphorylation and inactivation of the translation inhibitor 4E‐BP1.B
Age‐dependent Ph‐4EBP1/4E‐BP1 ratio in COX10 KO expressed relative to same age CTL set at 1.C
Representative western blot of muscle lysates from 200‐day‐old mice separated by denaturing SDS–PAGE and probed for Ph‐4E‐BP1 and for 4E‐BP1.D
Age‐dependent protein levels of Ph‐4E‐BP1 in COX10 KO muscle estimated by band densitometry normalized by GAPDH expressed relative to 50‐days COX10 KO set at 1.E
Western blots of muscle lysates from 50‐, 100‐, and 200‐day‐old COX10 KO mice, separated by denaturing SDS–PAGE and probed for Ph‐4E‐BP1 and GAPDH.F–O
Age‐dependent protein levels of SESTRIN2 (F), REDD1 (H), LC3II (J), MURF1 (L), and ATROGIN1 (N) estimated by band densitometry normalized by GAPDH, in COX10 KO expressed relative to same age CTL set at 1. Representative western blot of muscle lysates from 100‐ and 170‐ to 200‐day‐old mice separated by denaturing SDS–PAGE and probed for SESTRIN 2 (G), REDD1 (I), LC3II (K), MURF1 (M), ATROGIN1 (O), and GAPDH (G, I, K, M, O).P
Ph‐4EBP1/4E‐BP1 ratio and SESTRIN 2 protein levels normalized by α‐actin, estimated by band densitometry, in MERRF muscle expressed relative to CTL muscle set at 1.Q
Representative western blot of MERRF and CTL muscle lysates separated by denaturing SDS–PAGE and probed for Ph‐4EBP1, 4EBP1, SESTRIN 2, and α‐actin. Data information: In panels (B, F, H, J, L, and N), data are presented as Mean ± SD. COX10 KO (*n* = 4), CTL (*n* = 4). *P < 0.05 COX10 KO versus same age CTL. In panel (D), data are presented as Mean ± SD. ^#^ *P* < 0.05 COX10 KO versus COX10 KO at different ages. 50 days (*n* = 3), 100 days (*n* = 3), 200 days (*n* = 3). In panel (P), data are presented as Mean ± SD. MERRF (*n* = 4), CTL (*n* = 4). *P < 0.05 MERRF versus CTL. Statistically significant differences between the two groups for all panels were estimated by unpaired two‐tailed Student's test. Source data are available online for this figure.

Increased muscle amino acid oxidation, decreased muscle mass, and progressive weight loss in COX10 KO mice suggest a net negative balance of muscle proteins caused by increased breakdown, decreased synthesis, or a combination of both. One of the major regulators of muscle mass, mTORC1, stimulates *de novo* synthesis of proteins, nucleotides, and lipids, as well as inhibits autophagy. Therefore, we investigated whether mTORC1 regulation is involved in COX10 KO muscle proteolysis. The phosphorylation of the eukaryotic translation initiation factor 4E‐binding protein 1 (4E‐BP1) by mTORC1 causes the release of eukaryotic translation initiation factor 4E (eIF4E). Once free to combine with the translation initiation complex, eIF4E promotes cap‐dependent protein translation (Fig 3A, Qin *et al*, 2016; Broer & Broer, 2017). In COX10 KO mice, the levels of phospho‐4E‐BP1 relative to CTL levels (phospho‐4E‐BP1/total 4EBP1) were upregulated at 50 days, downregulated at 100 days, and unchanged at 200 days (Fig 3B and C; Appendix Fig S3A). The decrease in 4E‐BP1 phosphorylation at 100 days would predictably downregulate muscle protein translation. Longitudinal comparison within the COX10 KO muscle samples confirms decreased 4E‐BP1 phosphorylation at 100 days versus 50 days (40% decrease, Fig 3D and E) and a relative increase at 200 days versus 100 days (150% increase, Fig 3D and E). Interestingly, protein levels of total 4E‐BP1 are highly upregulated at 200 days versus 50 days (200% increase) and 100 days (150% increase), (Appendix Fig S3B and C). Therefore, in the presence of such elevated levels of total 4E‐BP1, the unphosphorylated fraction of the protein may still inhibit protein synthesis at 200 days, despite a phospho‐4E‐BP1/4E‐BP1 ratio similar to CTL. Unbiased transcriptomics of muscle RNA at 200 days of age highlighted the overexpression of two genes involved in mTORC1 inhibition, *Sesn2* (SESTRIN2) and DNA‐damage‐inducible transcript 4 (*Ddit4*, REDD1) (Ben‐Sahra *et al*, 2013; Parmigiani *et al*, 2014; Britto *et al*, 2018) in COX10 KO muscle (Appendix Fig S3D). To support these findings, we performed age‐dependent western blot analyses of these proteins which showed unchanged levels of SESTRIN2 and REDD1 at 50 days, followed by strong upregulation at 100 days (5.3‐ and 6.6‐fold, respectively, Fig 3F–I), which persisted although to a lesser extent at 200 days (1.9‐ and 2.5‐fold, respectively, Fig 3F–I). These findings suggest that SESTRIN2 and REDD1 are likely involved in mTORC1 inhibition at 100 days.

Next, we investigated mTORC1 signaling in MERRF muscle. Phosphorylation levels of 4EBP1 (Phospho‐4EBP1/4EBP1 ratio, Fig 3P and Q) were similar in MERRF and CTL muscle, despite highly upregulated SESTRIN2 levels (7‐fold, Fig 3P and Q) in MERRF muscle.

In addition to mTORC1 signaling, two main proteolytic systems can contribute to muscle loss. The autophagy–lysosome system removes dysfunctional organelles, protein aggregates, and unfolded proteins, whereas the ubiquitin–proteasome system degrades predominantly myofibrillar proteins. Therefore, we measured markers of autophagy and proteasomal activity at different ages to assess their contribution to muscle proteostasis in COX10 KO mice. The levels of the autophagy marker proteins LC3II and p62 were significantly elevated only at the late stage of myopathy (90 and 44% increase, respectively, Fig 3J and K; Appendix Fig S3E and F). On the other hand, the levels of the proteasomal activity markers ATROGIN1 and MURF1 were unchanged throughout the lifespan (Fig 3L–O), suggesting that the proteasome system is not a significant contributor to muscle loss in COX10 KO mice.

In summary, decreased 4E‐BP1 phosphorylation at 100 days coincides with the age at which COX10 KO mice start showing a decline in body weight, suggesting that mTORC1‐mediated protein synthesis inhibition plays a key role in initial arrest of muscle growth. Later, autophagy‐mediated protein degradation, together with a persistent 4E‐BP1‐mediated protein translation inhibition, contributes to the negative balance of muscle proteins.

### ISR^mt^ activation with systemic release of FGF21 is an early event in COX10 KO myopathy

**Figure 4:**
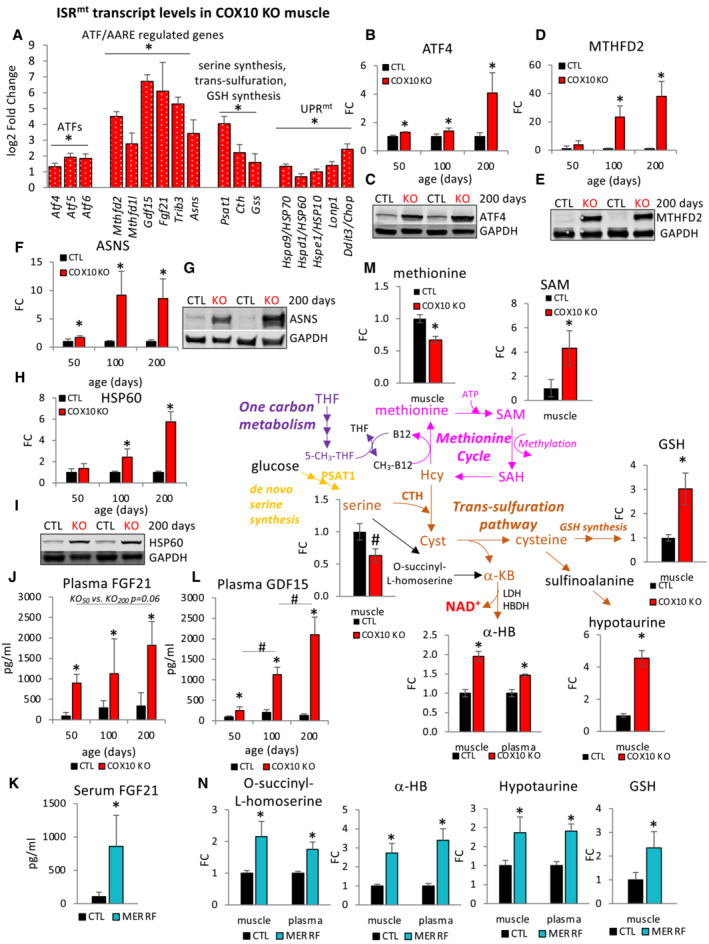
OXPHOS defective muscle triggers activation of ISR^mt^ and upregulation of the trans‐sulfuration pathway for GSH and α‐HB synthesis A
Muscle transcript levels of ISR^mt^ genes in 200‐day‐old COX10 KO expressed as log2 fold change relative to CTL.B–I
Age‐dependent levels of ATF4 (B), MTHFD2 (D), ASNS (F), and HSP60 (H), estimated by band densitometry normalized by GAPDH, in COX10 KO expressed relative to same age CTL set at 1. Representative western blot of muscle lysates from 200‐day‐old mice separated by denaturing SDS‐PAGE and probed for ATF4 (C), MTHFD2 (E), ASNS (G), and HSP60 (I) and GAPDH (C, E, G, I).J
Plasma concentration of FGF21 in 50‐, 100‐, and 200‐day‐old mice. Data are presented as Mean ± SD. *P < 0.05 COX10 KO versus same age CTL.K
Serum concentration of FGF21 in MERRF and CTL patients.L
Plasma concentration of GDF15 in 50‐, 100‐, and 200‐ (*n* = 4 per genotype) day‐old mice.M
Levels of metabolites of methionine cycle and trans‐sulfuration pathway in 200‐day‐ old COX10KO muscle and plasma expressed relative to CTL muscle and plasma, and levels of GSH in 200‐day‐old COX10KO muscle expressed relative to CTL muscle by LC–MS analysis. α‐HB, α‐hydroxybutyrate; GSH, glutathione; SAM, S‐adenosyl methionine; SAH, S‐adenosyl homocysteine; Hcy, homocysteine; Cyst, cystathionine; α‐KB, α‐ketobutyrate; THF, tetrahydrofolate; 5‐CH_3_‐THF, methyl tetrahydrofolate.N
Levels of metabolites of trans‐sulfuration pathway by LC–MS analysis in MERRF muscle and plasma expressed relative to CTL muscle and plasma. α‐HB, α‐hydroxybutyrate; GSH, glutathione. Data information: In panel (A), data are presented as Mean ± SE. *P.adj < 0.05 COX10 KO versus CTL. COX10 KO (*n* = 6), CTL (*n* = 6). Two‐tail unpaired Student's *t*‐tests were used for sample comparisons. Corrected *P* values were calculated based on the Benjamini–Hochberg method to adjust for multiple comparisons. In panels (B–I), data are presented as Mean ± SD. *P < 0.05 COX10 KO versus same age CTL. For each age, COX10 KO (*n* = 4), CTL (*n* = 4). In panel J, data are presented as Mean ± SD. 50 and 200 days: COX10 KO (*n* = 3), CTL (*n* = 3). 100 days: COX10 KO (*n* = 6), CTL (*n* = 6). In panel (K), data are presented as Mean ± SD. MERRF (*n* = 9), CTL (*n* = 25). *P < 0.05 MERRF versus CTL. In panel (L), data are presented as Mean ± SD. 50, 100, 200 days: COX10 KO (*n* = 4), CTL (*n* = 4). *P < 0.05 COX10 KO versus same age CTL. ^#^ *P* < 0.05 COX10 KO at 100 days versus COX10 KO at 50 and 200 days. In panel (M), data are presented as Mean ± SEM. For methionine, SAM, serine, hypotaurine, and α‐HB in muscle and plasma: COX10 KO (*n* = 5), CTL (*n* = 6). For GSH, measurements in muscle and plasma: COX10 KO (*n* = 3), CTL (*n* = 6). *P < 0.05; ^#^ *P* = 0.05 COX10 KO versus CTL. In panel (N), data are presented as Mean ± SEM. Muscle: MERRF (*n* = 10), CTL (*n* = 15). Plasma: MERRF (*n* = 9), CTL (*n* = 25). *P < 0.05 MERRF versus CTL. Statistically significant differences between the two groups for all panels were estimated by unpaired two‐tailed Student's test. Source data are available online for this figure.

The mitochondrial integrated stress response (ISR^mt^) is an elaborate signaling pathway activated in mammalian cells in response to mitochondrial damage and dysfunction to preserve metabolic homeostasis (Khan *et al*, 2017; Mick *et al*, 2020; Ahola *et al*, 2022). For example, in murine skeletal muscle ISR^mt^ can be activated by mild mitochondrial dysfunction associated with mtDNA deletions (Forsstrom *et al*, 2019). Importantly, both in mouse models and human mitochondrial myopathy, the ISR^mt^ has been associated with profound metabolic rewiring (Nikkanen *et al*, 2016). Adaptive metabolic changes occur not only in muscle but also systemically via secretion of signaling myokines such as fibroblast growth factor 21 (FGF21) (Tyynismaa *et al*, 2010) and growth differentiation factor 15 (GDF15) (Forsstrom *et al*, 2019). In accord, in COX10 KO muscle at 200 days, we found transcriptional upregulation of key genes coordinating the ISR^mt^, including activating transcription factors (*ATFs*) and ATF‐regulated genes, phosphoserine aminotransferase 1 (*Psat1*), cystathionine gamma‐lyase (*Cth*), glutathione synthetase (*Gss*), and mitochondrial unfolded protein response genes (UPR^mt^) (Fig 4A). To investigate temporal activation of ISR^mt^, we quantified age‐dependent changes in the levels of key ISR^mt^ proteins. Upregulation of ATF4 was detected at early disease stage and increased progressively with age relative to CTL muscle (1.3‐, 1.4‐, and 4‐fold at 50, 100, and 200 days, respectively, Fig 4B and C) and to COX10 KO at 50 days in longitudinal comparison within the COX10 KO muscle samples (Appendix Fig S4A and B). Upregulation of Methylenetetrahydrofolate dehydrogenase 2 (MTHFD2), an ATF4‐induced key enzyme of mitochondrial one carbon metabolism (Ben‐Sahra *et al*, 2016), was detected at the intermediate stage of myopathy and increased with age (23‐ and 38‐fold at 100 and 200 days, respectively, Fig 4D and E; Appendix Fig S4C and D). ATF4‐mediated upregulation of asparagine synthetase (ASNS) was found at the early stage of myopathy and increased markedly at later stages (1.7‐, 9‐, and 9‐fold at 50, 100, and 200 days, respectively, Fig 4F and G). Upregulation of the mitochondrial 60 kDa heat shock protein (HSP60), a component of the UPR^mt^, was detected at 100 days and increased at 200 days (2‐ and 6‐fold increase, at 100 and 200 days, respectively, Fig 4H and I; Appendix Fig S4E and F). Interestingly, ATF4 and ASNS protein levels were upregulated also in MERRF muscle (3‐and 8.5‐fold, respectively, Appendix Fig S4G and H), consistent with ISR^mt^ activation.

One of the most sensitive targets of ATF4, with three amino acid response elements (AARE) in its promoter region, is FGF21 (Maruyama *et al*, 2016), the hormone‐like myokine with autocrine and endocrine properties that serves as a primary coordinator of the ISR^mt^ in muscle (Khan *et al*, 2020). We found increased transcript levels of *Fgf21* in COX10 KO muscle (Fig 4A) and chronically elevated levels of circulating FGF21 protein commencing from the earliest stage of myopathy (9‐, 4‐, and 5‐fold at 50, 100, and 200 days, respectively, Fig 4J). Since liver can be a source of FGF21 in conditions of starvation (Badman *et al*, 2007), we investigated the potential hepatic contribution to circulating FGF21 levels in COX10 KO mice. Transcript levels of *Fgf21*, as well as most of the ISR^mt^ genes, were not upregulated in liver which is not affected by COX deficiency in this mouse model (Appendix Fig S4I), indicating that muscle is the primary contributor to circulating levels of FGF21. Notably, FGF21 is considered an established biomarker of human mitochondrial myopathy (Suomalainen, 2013), and as expected, plasma FGF21 levels were also increased in MERRF patients (8‐fold, Fig 4K).

Another myokine regulated by ISR^mt^ is GDF15, often linked to mitochondrial stress and dysfunction (Johann *et al*, 2021). In accord, we found increased transcript levels of *Gdf15* in COX10 KO muscle (Fig 4A). This corresponded to elevated plasma levels of GDF15, starting from the early myopathy stage and increasing with age (3‐, 6‐, and 16‐fold change at 50, 100, and 200 days, respectively, Fig 4L).

ISR^mt^ in muscle activates the one‐carbon metabolism and *de novo* serine synthesis, which converge on the trans‐sulfuration pathway for *de novo* cysteine and glutathione (GSH) biosynthesis (Fig 4M). In accord with trans‐sulfuration pathway activation, several products and intermediates were increased in COX10 KO muscle (i.e., GSH, hypotaurine, and α‐hydroxybutyrate) and plasma (α‐hydroxybutyrate) (Fig 4M). Moreover, reduced levels of muscle serine (Fig 4M) were likely the result of upregulated transcription of Cystathionine gamma‐lyase (CTH, Fig 4A), the rate‐limiting step of trans‐sulfuration which mediates the condensation of serine with homocysteine to form cystathionine (Fig 4M). Furthermore, upregulated CTH and increased synthesis of S‐adenosyl methionine (SAM), (Fig 4M), could be the mechanistic basis for the observed decrease in levels of the essential amino acid methionine (Fig 4M). Importantly, increased muscle GSH levels and elevated muscle and plasma intermediates of the trans‐sulfuration pathway were also found in MERRF patients (Fig 4N), consistent with ISR^mt^ and trans‐sulfuration also being activated. Notably, trans‐sulfuration pathway activation affects homeostasis of methionine, which was found to be decreased in both MERRF muscle and plasma (Fig 1B and C). In addition, by stimulating cysteine synthesis, the trans‐sulfuration likely contributes to the increased levels of circulating cysteine observed in MERRF patients (Fig 1C).

Together, these results indicate that COX deficiency restricted to skeletal muscle is sufficient to elicit progressive activation of ISR^mt^ and systemic release of FGF21 and GDF15 into the circulation, starting at an early disease stage. Furthermore, these responses in the COX10 KO mouse model recapitulate findings from studies of symptomatic MERRF patient muscle and plasma.

### Impaired fatty acid oxidation in OXPHOS defective muscle results in lipid accumulation

**Figure 5:**
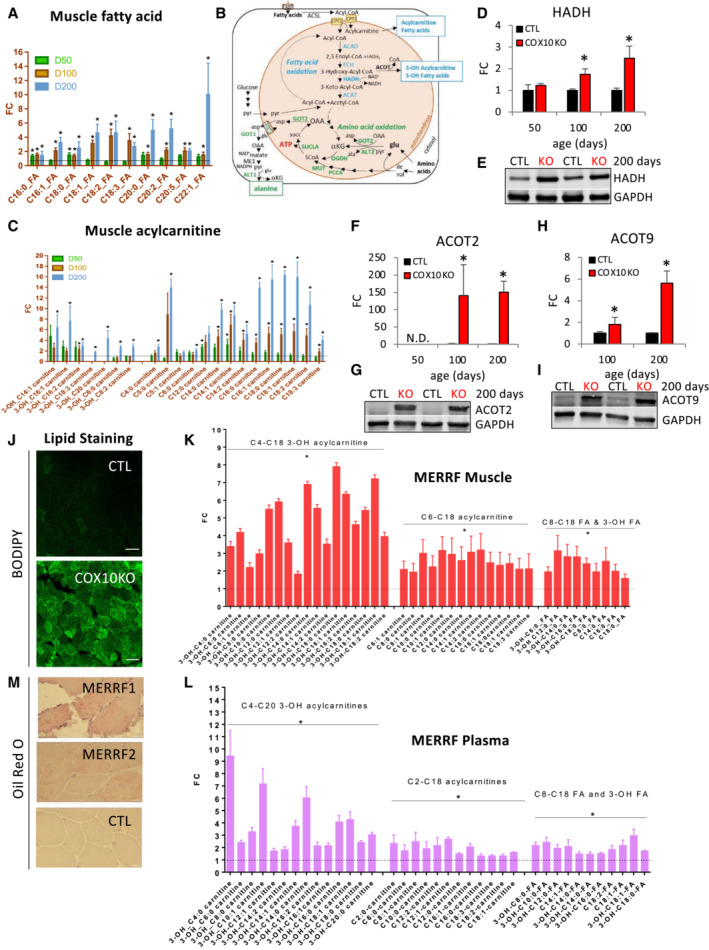
Lipid metabolism is altered in COX10 KO and MERRF muscle A
Muscle levels of fatty acids by LC–MS analysis in COX10 KO expressed relative to same age CTL.B
Schematic representation of pathways of fatty acid (blue) and amino acid (green) oxidation in COX10 KO muscle. 3‐OH‐fatty acids, 3‐hydroxy fatty acid; 3‐OH‐acylcarnitine, 3‐hydroxy acylcarnitine; CoA, Coenzyme A; CPT1, carnitine acyltransferase 1; CPT2, carnitine acyltransferase 2; ACAD, acyl CoA dehydrogenase; ECH, enoyl‐CoA hydratase; HADH, 3‐hydroxyacyl‐CoA dehydrogenase; ACAT, 3‐ketoacyl‐CoA thiolase; ACOT, acyl‐CoA thioesterase.C
Muscle levels of acylcarnitine and 3‐OH carnitines by LC–MS analysis in COX10 KO expressed relative to same age CTL.D–I
Age‐dependent levels of HADH (D), ACOT2 (F), and ACOT9 (H), estimated by band densitometry normalized by GAPDH, in COX10 KO expressed relative to same age CTL set at 1. Representative western blot of muscle lysates from 200‐day‐old mice separated by denaturing SDS–PAGE and probed for HADH (E), ACOT2 (G), ACOT9 (I), and GAPDH (E, G, I).J
Image of calf muscle cross section stained for lipids with BODIPY (green) from 200‐day‐old COX10 KO and CTL muscle. Images are taken at 40X magnification; bar scale, 50 μm.K, L
Levels of acylcarnitine, 3‐OH acylcarnitine, fatty acid and 3‐OH fatty acid in MERRF muscle (K) and plasma (L) by LC–MS analysis, expressed relative to CTL.M
Image of deltoid muscle cross section stained for lipids with Oil red O (red) from two MERRF patients and one CTL. Images are taken at 20X magnification; bar scale, 50 μm. Data information: In panels (A, C, K, and L), data by LC–MS analysis are presented as Mean ± SEM. In panels A and C, 50, 100, 200 days: COX10 KO (*n* = 6), CTL (*n* = 6). *P < 0.05 COX10 KO versus same age CTL. In panels (D, F, and H), data are presented as Mean ± SD. COX10 KO (*n* = 4), CTL (*n* = 4). *P < 0.05 COX10 KO versus same age CTL. In panels (K and L), muscle: MERRF (*n* = 10), CTL (*n* = 15); plasma: MERRF (*n* = 9), CTL (*n* = 25). *P < 0.05 MERRF versus CTL. Statistically significant differences between the two groups for all panels were estimated by unpaired two‐tailed Student's test. Source data are available online for this figure.

FGF21 is known to induce lipolysis in white adipose tissue (WAT) and fatty acid oxidation in other tissues, including skeletal muscle (Tezze *et al*, 2019). We found that levels of fatty acids were elevated in COX10 KO plasma (Appendix Fig S5A and B) and muscle (Fig 5A), suggesting that increased WAT lipolysis mediated through FGF21 signaling may be responsible for free fatty acid release into the circulation and uptake by skeletal muscle. After transport into muscle via fatty acid‐binding proteins (FABPs), fatty acids are converted to acyl‐CoAs (by Acyl‐CoA synthetases, such as the long chain synthase, ACSL). These acyl‐CoAs enter mitochondria as acyl‐carnitines (by Carnitine palmitoyltransferase 1, CPT1) and are then converted back to acyl‐CoAs (by Carnitine palmitoyltransferase 2, CPT2). In mitochondria, acyl CoAs are oxidized by the process of β‐oxidation, sequentially catalyzed by Acyl CoA dehydrogenase (ACAD), Enoyl‐CoA hydratase (ECH), 3‐Hydroxyacyl‐CoA dehydrogenase (HADH), and 3‐Ketoacyl‐CoA thiolase (ACAT1) (Fig 5B). Significant transcriptional upregulation of several fatty acid metabolic genes in COX10 KO muscle, including *Fabp3*, *Acsl1*, *Cps2*, *Acadl*, *Echs1*, *Hadha*, and *Hadhb* (Appendix Fig S5C), indicated an increased fatty acid uptake in muscle to drive β‐oxidation. However, we found increased levels of muscle acyl carnitines and hydroxy‐acyl carnitines that bear a hydroxyl group on the third carbon (3‐OH acyl carnitines), (Fig 5C). Increased levels of 3‐OH acyl carnitines strongly suggest lipid accumulation due to incomplete β‐oxidation in COX10 KO muscle. In fact, in the third step of β‐oxidation of acyl‐CoAs, HADH catalyzes the formation of 3‐ketoacyl‐CoA from 3‐L‐hydroxyacyl‐CoA and NAD^+^ (Appendix Fig S5D). Hence, we surmise that progressive accumulation of 3‐OH acyl forms in COX10 KO muscle (Fig 5C) is due to impaired HADH activity. Since HADH protein levels were elevated (1.7‐and 2.5‐fold at 100 and 200 days, respectively, Fig 5D and E), the most likely explanation is that HADH activity is limited by NAD^+^ availability due to suppressed oxidation of NADH to NAD^+^ resulting from attenuated OXPHOS activity. In accord with this interpretation, we found elevated NADH levels and a decreased NAD^+^/NADH ratio in COX10 KO versus CTL muscle (Appendix Fig S5E).

Further impacting fatty acid metabolism, we found highly elevated protein levels of the mitochondrial Acyl‐CoA thioesterase 2 (ACOT2, 140‐ and 150‐fold at 100 and 200 days, respectively, Fig 5F and G) and mitochondrial ACOT9 (1.8‐ and 5.6‐fold change at 100 and 200 days, respectively, Fig 5H and I). Interestingly, upregulation of ACOTs, which catalyze the hydrolysis of acyl‐CoA esters to free fatty acids and CoA, mitigates mitochondrial fatty acid overload and prevents CoA limitation during incomplete fatty acid oxidation (Moffat *et al*, 2014; Bekeova *et al*, 2019). Thus, increased levels of ACOTs further confirm that β‐oxidation is impaired in COX10 KO muscle. After CoA removal, free fatty acids and fatty acid oxidation intermediates are transported out of the mitochondria as acyl carnitines and then released into circulation. Therefore, progressive increase in ACOT activity can explain the age‐dependent accumulation of acyl‐carnitines, in both muscle and plasma of COX10 KO mice (Fig 5C; Appendix Fig S5F). Moreover, a combination of chronic WAT lipolysis and impaired β‐oxidation could be responsible for the temporal increase in fatty acids in COX10 KO muscle, which accumulate in the form of lipid droplets (Fig 5J; Appendix Fig S5G).

Alteration of lipid metabolism was also detected in patient‐derived MERRF muscle and plasma. Similar to COX10 KO mice, the MERRF muscle lipid profile is characterized by increased levels of acyl‐carnitines, 3‐OH acyl‐carnitines, fatty acids, and 3‐OH fatty acids (Fig 5K). In accord with skeletal muscle being the major contributor to the circulating acyl‐carnitine pool (Koves *et al*, 2008), increased plasma levels of acyl‐carnitines and 3‐OH acyl‐carnitines (Fig 5L) mirrored the muscle lipid profile. Accumulation of 3‐OH acyl forms in muscle and plasma, in combination with altered redox homeostasis (Appendix Fig S5H), strongly suggests that, similar to COX10 KO mice, impaired β‐oxidation in human patients can be attributed to decreased NAD^+^‐dependent HADH activity. Moreover, as in COX10 KO muscle, lipid droplets accumulate in human MERRF muscle (Fig 5M).

### OXPHOS defective muscle activates non‐anaplerotic pathways of glutamate metabolism for mitochondrial NAD ^+^ regeneration and nitrogen disposal

**Figure 6:**
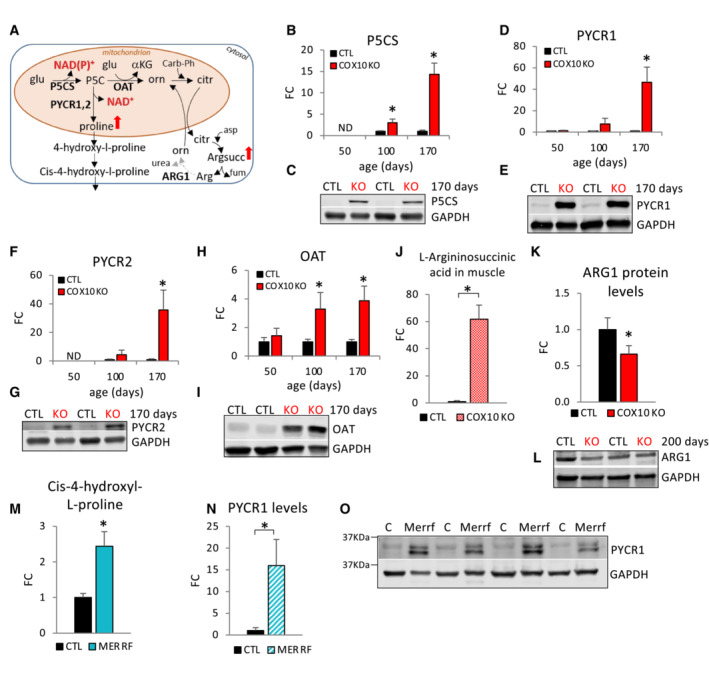
Alternative pathways of mitochondrial NADH oxidation and ammonia disposal are activated in OXPHOS defective muscle A
Schematic representation of non‐anaplerotic pathways of glutamate utilization in MERRF and COX10 KO muscle. glu, glutamate; α‐KG, α‐ketoglutarate; P5C, pyrroline‐5‐carboxylate; orn, ornithine; citr, citrulline; carb‐Ph, carbamoyl‐phosphate; argsucc, L‐argininosuccinic acid; arg, arginine; P5CS, pyrroline‐5‐carboxylate synthase; OAT, ornithine aminotransferase; PYCR1, pyrroline‐5‐carboxylate reductase 1; PYCR2, pyrroline‐5‐carboxylate reductase 2; ARG1, arginase 1. Enzymes in bold indicate regulated steps detected by western blot analysis. The dashed arrow indicates downregulation of ARG1.B–I
Age‐dependent muscle protein levels of P5CS (B), PYCR1 (D), PYCR2 (F) OAT (H), estimated by band densitometry normalized by GAPDH, in COX10 KO expressed relative to same age CTL set at 1. Data are presented as Mean ± SD. *P < 0.05 COX10 KO versus same age CTL. Representative western blots of muscle lysates from 170‐day‐old mice separated by denaturing SDS–PAGE and probed for P5CS (C), PYCR1 (E), PYCR2 (G), OAT (I), and GAPDH (C, E, G, I).J
Muscle levels of L‐argininosuccinic acid by LC–MS analysis, in 200‐day‐old COX10 KO expressed relative to CTR set at 1.K
Muscle protein levels of ARG1, estimated by band densitometry normalized by GAPDH, in 200‐day‐old COX10 KO expressed relative to CTL set at 1.L
Representative western blot of muscle lysates from 200‐day‐old mice separated by denaturing SDS–PAGE and probed for ARG1 and GAPDH.M
MERRF plasma levels of 4‐hydroxyl‐L‐proline by LC–MS analysis, expressed relative to CTL value set at 1.N
PYCR1 protein levels, estimated by band densitometry normalized by GAPDH, in MERRF muscle expressed relative to CTL muscle.O
Western blots of MERRF and CTL muscle lysates separated by denaturing SDS–PAGE and probed for PYCR1 and GAPDH. Data information: In panels (B, D, F, H, and K), data are normalized to same age CTL set to 1. Data are presented as Mean ± SD. 50, 100, 200 days: COX10 KO (*n* = 4), CTL (*n* = 4). *P < 0.05 COX10 KO versus CTL. In panel (J), data are presented as Mean ± SEM. COX10KO (*n* = 6), CTL (*n* = 6). *P < 0.05 COX10 KO versus CTL. In panel (M), data are presented as Mean ± SEM. MERRF (*n* = 9), CTL (*n* = 25). In panel (N), data are presented as Mean ± SD. MERRF (*n* = 4), CTL (*n* = 4). *P < 0.05 MERRF versus CTL. Statistically significant differences between the two groups for all panels were estimated by unpaired two‐tailed Student's test. Source data are available online for this figure.

We hypothesized that COX10 KO muscle responds to an inadequate mitochondrial NAD^+^ pool by upregulating a compensatory, OXPHOS‐independent pathway for matrix NADH oxidation achieved by the shunting of mitochondrial glutamate toward proline synthesis (Fig 6A). In this NAD^+^ repletion pathway, glutamate is initially converted to pyrroline‐5‐carboxylate (P5C) by Pyrroline‐5‐carboxylate synthase (P5CS), generating NAD(P)^+^. Then, P5C is converted to proline by the Pyrroline‐5‐carboxylate reductases 1 and 2 (PYCR1 and PYCR2), generating NAD^+^. In accord with the upregulation of this NAD^+^‐generating pathway, we found increased levels of muscle proline (200%, Appendix Fig S6A) as well as evidence of pathway upregulation at both the transcriptional (Appendix Fig S6B) and protein levels (Fig 6B–G). While P5CS upregulation was detected at 100 days and progressed with age (3‐ and 14‐fold, at 100 and 170 days, respectively, Fig 6B and C; Appendix Fig S6C and D), PYCR1 and PYCR2 levels were highly upregulated only at late disease stage (47‐ and 36‐fold, respectively, at 170 days; Fig 6D–G; Appendix Fig S6E and F).

As an alternative to proline synthesis, glutamate‐derived P5C can receive the amino group of glutamate to yield ornithine and αKG as co‐products, a reaction catalyzed by ornithine aminotransferase (OAT, Fig 6A). While αKG feeds into the TCA cycle, ornithine enters the urea cycle as citrulline, which subsequently condensates with aspartate to produce argininosuccinate (by argininosuccinate synthase, ASS). Increased levels of OAT (3‐ and 4‐fold at 100 and 170 days, respectively, Fig 6H and I; Appendix Fig S6F and G) and a dramatic increase in argininosuccinate (61‐fold at 200 days, Fig 6J) strongly suggest an upregulation of the glutamate‐P5C‐argininosuccinate pathway. In addition, the levels of argininosuccinate lyase (ASL), which converts argininosuccinate to arginine and fumarate, was increased (73%, Appendix Fig S6H and I), in tandem with decreased levels of arginase 1 (ARG1) in COX10 KO versus CTL muscle (34% decrease, Fig 6K and L). Because ARG1 mediates the conversion of arginine to urea, ARG1 decrease is a likely contributor to the accumulation of argininosuccinate. The condensation of citrulline and aspartate may also contribute to the removal of OAA‐derived aspartate, which could facilitate glutamate oxidation through the TCA cycle. In addition, since it carries four amino groups, increased production of argininosuccinate could provide an alternative mechanism for disposal of nitrogen for facilitated amino acid catabolism in COX10 KO muscle.

Upregulation of the glutamate‐proline pathway was also detected in MERRF muscle. Decreased levels of muscle proline (Fig 1B) accompanied by increased plasma levels of the proline‐derivative 4‐hydroxyl‐L‐proline (Fig 6M) and increased levels of PYCR1 in muscle (Fig 6N and O) strongly suggest increased conversion of glutamate to proline for NAD^+^ regeneration (Fig 6A).

It was previously shown that during respiratory inhibition and anoxic conditions, which are functionally comparable to COX deficiency, mitochondrial diaphorases can contribute up to 80% to the NAD^+^ matrix pool and support OGDHC‐mediated substrate‐level phosphorylation (Kiss *et al*, 2014). Diaphorases, also known as DT‐diaphorases or NAD(P)H:quinone oxidoreductases (NQO), are flavoenzymes catalyzing the oxidation of reduced pyridine nucleotides (both NADH and NADPH) by endogenous electron acceptors. Among the several diaphorases isoforms, NQO1 has been found to localize in the cytosol and mitochondria from several tissues (Dong *et al*, 2013). Interestingly, transcript levels of *Nqo1* were upregulated in COX10 KO versus CTL (Appendix Fig S6J) suggesting that DT‐diaphorases could contribute to the matrix NAD^+^ pool regeneration in COX10 KO muscle. Interestingly, the re‐oxidation of the reducible substrates of diaphorases is mediated by respiratory chain Complex III (Kiss *et al*, 2014), which would also allow SDH activity. However, the electron acceptor of reduced cytochrome c under conditions of oxygen deprivation or dysfunctional complex IV remains to be determined.

Another potential source of matrix NAD^+^ could derive from the reverse operation of isocitrate dehydrogenase (IDH2 and IDH3 isoforms). Reductive carboxylation of glutamate‐derived αKG would predictably generate M + 5 forms of citrate (i.e., with the TCA cycle running in a counter‐clockwise direction). This possibility was tested by tracing muscle incorporation of [^13^C5, ^15^N]‐glutamate into the TCA cycle intermediates. Our data show that muscle citrate displays an overall greater incorporation (Appendix Fig S6K), demonstrating increased oxidative flux of glutamate (i.e., with the TCA cycle running in a clockwise direction) in COX10 KO versus CTL muscle. However, M + 5 citrate was not detected, indicating that reductive carboxylation does not play a major role in NAD^+^ regeneration in COX10 KO muscle, at least in the time frame of the experiment (30 min).

Taken together, these data indicate that OXPHOS‐defective muscle adapts metabolically by implementing alternative pathways for the rewiring of redox and metabolic homeostasis.

### FGF21‐ and leptin‐mediated starvation‐like response activates glucocorticoid signaling

**Figure 7:**
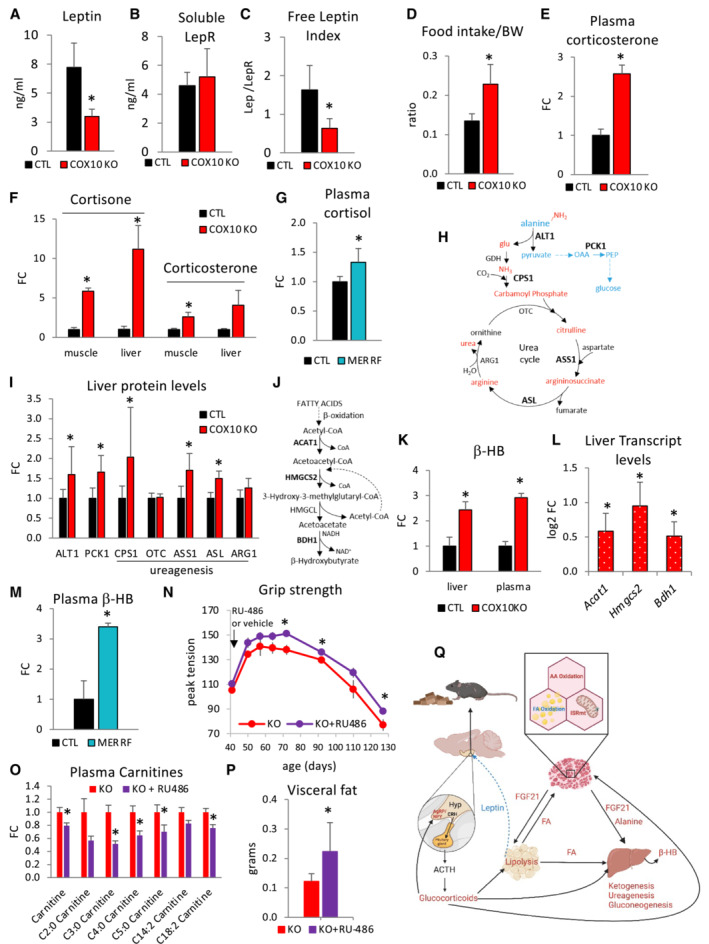
Leptin and glucocorticoids are altered in COX10 KO mice and MERRF patients A–C
Plasma levels of Leptin (A), soluble leptin receptor (LepR) (B), and free leptin index (C) by ELISA, in 200‐dayold CTL and COX10 KO mice.D
Daily food intake normalized by body weight (BW) in 200‐day‐old CTL and COX10 KO mice.E, F
Levels of glucocorticoids by LC–MS/MS analysis in COX10 KO plasma (E), liver, and muscle (F), at 200 days of age, expressed relative to CTL value set at 1.G
Levels of glucocorticoids in MERRF plasma by LC–MS analysis, expressed relative to CTL value set at 1.H
Schematic representation of hepatic alanine disposal through pathways of gluconeogenesis (blue) and ureagenesis (red). ALT1, alanine aminotransferase 1; PCK1, phosphoenolpyruvate carboxykinase 1; CPS1, carbamoyl‐phosphate synthase 1; OTC, ornithine transcarbamylase; ASS1, argininosuccinate synthase 1; ASL, argininosuccinate lyase; ARG1, arginase 1. Enzymes in bold indicate upregulated steps detected by western blot analysis.I
Liver protein levels of ALT1, PCK1, CPS1, OTC, ASS1, ASL, and ARG1, estimated by band densitometry normalized by β‐actin, in 200‐day‐old COX10 KO expressed relative to same age CTL set at 1.J
Schematic pathway of ketogenesis in liver: ACAT1, acetyl‐CoA acetyltransferase; HMGCS2, HMG‐CoA synthase; HMGCL, HMG‐CoA lyase; BDH1, β‐hydroxybutyrate dehydrogenase 1.K
Liver and plasma levels of β‐hydroxybutyrate (β‐HB) by LC–MS analysis, in 200 days old COX10 KO expressed relative to CTL set at 1.L
Liver transcript levels of *Acat1*, *Hmgcs2*, and *Bdh1* genes in 200‐day‐old COX10 KO expressed as log2 fold change relative to CTL. *P*.adj < 0.05 COX10 KO versus CTL. COX10 KO (*n* = 6), CTL (*n* = 6). Two‐tail unpaired Student's *t*‐tests were used for sample comparisons. Corrected *P* values were calculated based on the Benjamini–Hochberg method to adjust for multiple comparisons.M
Plasma levels of β‐HB by LC–MS analysis in MERRF expressed relative to CTL set at 1.N
Age‐dependent grip strength of COX10 KO mice treated with RU‐486 and vehicle. The black arrow indicates the beginning of the treatment at 43 days.O
Plasma levels of acylcarnitine in COX10 KO mice treated with RU‐486 or vehicle at 100 days of age, by LC–MS analysis, expressed relative to KO.P
Visceral fat deposits of COX10 KO mice treated with RU‐486 and vehicle at 100 days of age.Q
Schematics of the proposed inter‐organ hormonal signaling mechanisms leading to systemic metabolic responses in mitochondrial myopathy. FA, fatty acids; AA, amino acids; β‐HB, β‐hydroxybutyrate; CRH, Corticotrophin‐releasing hormone; ACTH, Adrenocorticotropic hormone; AgRP, Agouti‐related peptide; NPY, neuropeptide Y; Hyp, Hypothalamus. Increased metabolites, hormones, and pathways are labeled in red; decreased hormone and pathway are labeled in blue. Data information: In panels (A–C), data are presented as Mean ± SD. COX10 KO (*n* = 5), CTL (*n* = 5). *P < 0.05 COX10 KO versus CTL. In panel (D), data are presented as Mean ± SD. COX10 KO (*n* = 9), CTL (*n* = 10). In panels (E, F), data measured in duplicate are presented as Mean ± SEM. Plasma: COX10 KO (*n* = 3), CTL (*n* = 3). Liver: COX10 KO (*n* = 3), CTL (*n* = 3). Muscle: COX10 KO (*n* = 3), CTL (*n* = 3). *P < 0.05 COX10 KO versus CTL. In panels (G and M), data are presented as Mean ± SEM. MERRF (*n* = 9), CTL (*n* = 25). In panel (I), data are presented as Mean ± SD. ALT1, PCK1, and CPS1: COX10 KO (*n* = 8), CTL (*n* = 8). OTC, ASS1, ASL, and ARG1: COX10 KO (*n* = 4), CTL (*n* = 4).*P < 0.05 COX10 KO versus CTL. In panel (K), data are presented as Mean ± SEM. Liver: COX10 KO (*n* = 3), CTL (*n* = 3). Plasma: COX10 KO (*n* = 6), CTL (*n* = 6) *P < 0.05 COX10 KO versus CTL. In panel (L), data are presented as Mean ± SE. COX10 KO (*n* = 6), CTL (*n* = 6). In panels (N, O), data are presented as Mean ± SEM. Panel (N): KO + RU486 (*n* = 6), KO (*n* = 4). Panel O: KO + RU486 (*n* = 6), KO (*n* = 6). In panel (P), data are presented as Mean ± SD. KO + RU486 (*n* = 6), KO (*n* = 6). *P < 0.05 KO + RU486 versus KO. Statistically significant differences between the two groups for all panels were estimated by unpaired two‐tailed Student's test. Source data are available online for this figure.

FGF21 has an established role in lipid metabolism regulation through induction of a decrease in fat biomass, accelerated metabolic rate, and upregulation of critical target genes, including the Leptin receptor (LepR), Peroxisome proliferator‐activated receptor gamma coactivator 1‐alpha (PGC1α), and Uncoupling protein 1 (UCP1). WAT and liver are the primary mediators of endocrine FGF21 metabolic effects, which require the presence of β‐klotho protein (Adams *et al*, 2012). In agreement with increased levels of circulating FGF21 (Fig 4J), we found highly elevated hepatic expression of LepR and PGC1α in COX10 KO mice (16‐ and 2.6‐fold, respectively, Appendix Fig S7A). Leptin is produced primarily by adipose cells and contributes to regulation of adipose tissue mass by a central action in the control of appetite. Indeed, leptin suppresses appetite and increases energy expenditure by binding to and activating the membrane‐bound LepR (isoform b) in the hypothalamus (Cohen *et al*, 2005). Interestingly, leptin signaling can also be modulated by the soluble LepR (sLepR), which is mainly produced by liver through the cleavage and shedding of the membrane‐bound LepR (Cohen *et al*, 2005). To the extent that circulating leptin binds to its soluble receptor, as the leptin‐sLepR complex, it is incapable of activating the hypothalamic LepR. In 200‐day‐old COX10 KO mice, circulating leptin levels were decreased (58%, Fig 7A), whereas circulating sLepR levels were unchanged (Fig 7B) and the ratio of circulating leptin to sLepR (referred to as free leptin index, FLI), an indicator of active leptin (Yamada *et al*, 2019), was decreased (61%, Fig 7C). In 100‐day‐old COX10 KO mice, circulating leptin levels were unchanged, sLepR levels were increased (51%, Appendix Fig S7B), and FLI was decreased (40%, although not reaching statistical significance, Appendix Fig S7B).

Low circulating leptin levels reflect a loss of body fat and are used as a signal for the hypothalamus to increase food intake to regain adipose mass (Ahima & Osei, 2004). Chronic leptin deficiency in a genetic KO model (the Lep^ob/ob^ mouse) results in hyperphagia and obesity (Wang *et al*, 2014). Therefore, since leptin levels were low in COX10 KO mice, we investigated the potential for an increase in appetite by measuring daily food intake. COX10 KO daily food intake normalized to body weight was higher than CTL (Fig 7D), suggesting that attenuated levels of circulating leptin signal to the hypothalamus and trigger compensatory hyperphagia.

It has been recently shown that the stimulation of hunger by low leptin levels requires activation of the hypothalamic–pituitary–adrenocortical (HPA) axis, triggering the release of glucocorticoids (GCs) by the adrenal gland (Perry *et al*, 2019). In accord with low leptin‐mediated induction of the HPA axis and GC release, we found increased levels of corticosterone in plasma (2.6‐fold, Fig 7E; Appendix Fig S7C) and muscle (3‐fold, Fig 7F) and increased cortisone in muscle and liver of COX10 KO mice (6‐ and 11‐fold, respectively, Fig 7F). Interestingly, elevated levels of cortisol, the primary glucocorticoid in humans, were also found in MERRF plasma (33% increase, Fig 7G), indicating that activation of the HPA axis and hypercortisolemia also occurs in mitochondrial patients.

Increased corticosterone induces expression of the orexigenic (appetite stimulating factors), neuropeptide Y (NPY), and agouti‐related peptide (AgRP) in the arcuate nucleus of the hypothalamus to increase hunger (Perry *et al*, 2019). In accord with the activation of this GC‐mediated signaling pathway, hypothalamus transcriptomics revealed upregulated expression of *Agrp*, and *Npy* genes (Appendix Fig S7D), suggesting that low leptin stimulates the HPA axis and increases corticosterone which activates NPY/AgRP‐mediated orexigenic signaling triggering hyperphagia.

### Hepatic gluconeogenesis, ureagenesis, and ketogenesis are upregulated in COX10 KO

GCs are major regulators of glucose metabolism, stimulating multi‐organ processes that collectively maintain circulating glucose homeostasis. Among the metabolic effects of GCs, the stimulation of liver gluconeogenesis and the mobilization of amino acids from extrahepatic tissues is used as substrates for gluconeogenesis. Therefore, we looked for evidence of increased gluconeogenesis in COX10 KO mice and MERRF patients. Alanine, a glucogenic amino acid, was elevated in plasma from COX10 KO mice (Appendix Fig S7E) and MERRF patients (Fig 1C). Circulating alanine homeostasis is the result of skeletal muscle excretion, hepatic uptake, and metabolic disposal. Alanine taken up by the liver is converted by ALT1 to pyruvate and glutamate, carrying the carbon skeleton and the amino group of alanine, respectively (Fig 7H). Notably, while the amino group is ultimately converted to urea, the carbon skeleton provides a substrate for gluconeogenesis. Therefore, we investigated the pathways of alanine utilization in COX10 KO liver, which is unaffected by COX10 genetic excision and therefore fully‐retains COX activity. Consistent with increased alanine disposal, we found elevated levels of ALT1 (60% increase) and cytosolic phosphoenolpyruvate carboxykinase (PCK1, 66% increase) (Fig 7I; Appendix Fig S7F), which converts OAA to phosphoenolpyruvate (PEP) as a major entry point into the gluconeogenic pathway (Fig 7H). Notably, GCs promote gluconeogenesis by stimulating PCK1 transcription (Kuo *et al*, 2015). In accord with the increased disposal of muscle ammonia, we found elevated levels of three enzymes of the urea cycle (CPS1, 100%; ASS1, 70%; ASL, 50%, Fig 7I; Appendix Fig S7F). These results suggest that increased amino acid oxidation in muscle is accompanied by elevated alanine‐derived synthesis of glucose and urea in liver. They also suggest that increased circulating alanine, a telling biomarker for recognition of PMM patients (Mitochondrial Medicine Society's Committee on Diagnosis *et al*, 2008), could result from excessive skeletal muscle excretion which cannot be fully cleared by the liver.

Both FGF21 and GCs stimulate WAT lipolysis and trigger the release of fatty acids for muscle and liver utilization. The ketone body β‐hydroxybutyrate (β‐HB) is synthesized through liver ketogenesis from acetyl CoA generated by β‐oxidation (Fig 7J). β‐HB released into the circulation becomes available to support the energetic needs of peripheral organs to preserve glucose homeostasis. In accord with hepatic ketogenesis activation, we found elevated levels of β‐HB in COX10 KO liver and plasma at 200 days (2.4‐ and 2.9‐fold, respectively, Fig 7K) and upregulated transcript levels of three enzymes of the ketogenic pathway (*Acat1*, *Hmgcs2*, *Bdh1*, Fig 7L). Increased levels of plasma β‐HB were also detected at 50 and 100 days (2‐ and 3.4‐fold respectively, Appendix Fig S7G), suggesting that activation of liver ketogenesis is an early event. Interestingly, plasma β‐HB levels were also significantly increased in MERRF patients (3.4‐fold, Fig 7M). These results are indicative of a MERRF metabolic profile characterized by increased lipolysis and fatty acid availability (Fig 5L), elevated FGF21 (Fig 4K), and hypercortisolemia (Fig 7G), which stimulates ketogenesis.

### GCs signaling contributes to the systemic regulation of COX10 KO lipid dysmetabolism

To investigate if GCs signaling pathway participates in muscle and fat wasting and contributes to mitochondrial myopathy, we treated COX10 KO mice with the GC receptor (GR) antagonist RU‐486. It was previously shown that RU‐486 attenuates muscle atrophy in sepsis, cachexia, and starvation in rodents (Menconi *et al*, 2007), and prevents the lipolytic action of GCs (Xu *et al*, 2009). RU‐486 administration starting at 43 days of age improved muscle strength (10% increase at 70 days of age, Fig 7N) and this improvement persisted throughout the duration of the treatment. However, despite the muscle strength amelioration, RU‐486 did not prevent disease progression (Fig 7N; Appendix Fig S7H). An independent cohort of COX10 KO mice was treated for 50 days to investigate the metabolic effects of RU‐486. First, we found that FGF21 plasma levels were unchanged by RU‐486 (Appendix Fig S7I), confirming that GC response is downstream of ISR^mt^ signaling. Second, we detected a decrease in the plasma levels of several short‐chain acyl carnitines (Fig 7O) and an increase in visceral fat (84%, Fig 7P), indicating that GR inhibition reduces adipose store depletion and lipid mobilization in COX10 KO mice. Taken together, the improved muscle strength and the increase in lipid stores suggest that reducing fat wasting by targeting the GC signaling ameliorates mitochondrial myopathy.

## Discussion

The ramified metabolic consequences of OXPHOS dysfunction are under intense investigation to identify common metabolic pathway perturbations that can provide tractable therapeutic targets for diverse genetic forms of mitochondrial myopathies. In our study, we compared m.8344A>G MERRF human patients affected by severe mitochondrial myopathy to a mouse model of mitochondrial myopathy, the muscle‐specific COX10 KO mouse. The rationale behind the MERRF/COX10 KO comparison resides in their muscle bioenergetic, phenotypic, and metabolic similarities. The most common MERRF mutation is the m.8344A>G in the *MT‐TK* gene encoding for transfer RNA^Lysine^, which affects the translation of mtDNA‐encoded proteins. Although this mutation can potentially affect complex I, III, IV, and V assembly, complex IV is the most affected complex in MERRF muscle, as clearly shown by an abundance of COX‐negative fibers (Appendix Fig S1E) and impaired COX activity by biochemical testing (Antonicka *et al*, 1999; Catteruccia *et al*, 2015). On the other hand, the pathogenic m.3243A>G mutation in the *MT‐TL1* gene encoding mitochondrial tRNA^Leucine^ is often associated with complex I dysfunction (Sharma *et al*, 2021), suggesting that mutations in mitochondrial tRNAs can impair some respiratory chain complexes more severely than others. Thus, MERRF and COX10 KO muscles share severe COX deficiency despite their genetic differences. MERRF syndrome is a complex disease with possible multisystem involvement. However, the MERRF phenotype is poorly represented by its acronym (myoclonus epilepsy with ragged‐red fibers), because it is mostly characterized by myopathy (Mancuso *et al*, 2013; Catteruccia *et al*, 2015). Indeed, MERRF is categorized as one of the primary mitochondrial myopathies (PMM) (de Barcelos *et al*, 2019), a genetically defined group of disorders leading to defects of OXPHOS affecting predominantly skeletal muscle (Mancuso *et al*, 2017).

We postulated that metabolic responses to OXPHOS defects could lead to a common adaptive process that drives muscle degeneration in both mice and humans. To test this hypothesis, integrated multi‐omic profiles were investigated to recognize metabolic pathway perturbations common to MERRF human patients and the COX10 KO mouse. Our results confirm the hypothesis that shared metabolic events underlie the pathogenesis of mitochondrial myopathy in mice and humans, and unveil an inter‐organ crosstalk and fundamental systemic features of PMM.

In COX10 KO muscle, upregulated amino acid oxidation through the TCA cycle commences at an early stage of myopathy and increases with age. The accelerated catabolism of glutamate for energy generation is driven by the progressive upregulation of specific mitochondrial aminotransferases to feed the TCA cycle for anaplerotic replenishment. Treatment with aminotransferases inhibitor AOA reduces exercise endurance on the treadmill, suggesting that upregulation of the energy‐generating glutamate flux through the TCA cycle is an adaptive response in COX10 KO muscle. Of note, because of the non‐selective inhibition of AOA (e.g., AOA is also inhibitor of cystathionine β synthase, CBS; cystathionine γ lyase, CSE; GABA aminotransferase, GABA‐T; ornithine aminotransferase, OAT) we cannot exclude that suppression of additional pathways (trans‐sulfuration, GABA, arginine metabolism) could contribute to the worsening of COX10 KO exercise endurance. Further investigation would be required to dissect specific pathway contribution and fully address this point.

Since transaminase enzyme directionality is determined by the relative amounts of substrates and products, sustained oxidative influx of glutamate into the TCA cycle requires that the resulting aminated species, such as OAA‐derived aspartate and pyruvate‐derived alanine, are continually removed from mitochondria (cataplerosis). This explains our findings that alanine derived by aspartate (via ALT1) and pyruvate (via ALT2) is efficiently excreted from muscle. In liver, alanine is used for gluconeogenesis and ureagenesis. Overall, multi‐omics findings are consistent with a metabolic remodeling typified by increased muscle amino acid oxidation and hepatic gluconeogenesis and ureagenesis. MERRF patient muscles also show increased amino acid oxidation and alanine release into the circulation.

Loss of muscle protein occurs when net breakdown exceeds synthesis. Therefore, the elevated free amino acid levels in OXPHOS defective muscle can derive from an increased rate of proteolysis, a decreased rate of synthesis, or both. Unphosphorylated 4E‐BP1 is an inhibitor of protein translation through its binding to eIF4E and consequent prevention of translation initiation complex formation that is required for cap‐dependent protein translation (Showkat *et al*, 2014). In 100 days old COX10 KO mice, we found a decreased fraction of phosphorylated 4E‐BP1 (phospho‐4E‐BP1/total 4E‐BP1, Fig 3B), suggesting that slowed protein translation contributes to decreased muscle mass. Furthermore, phosphorylation of 4E‐BP1 is directly catalyzed by mTORC1, and mTORC1 signaling is considered the main contributor to 4E‐BP1 phosphorylation (Showkat *et al*, 2014). Therefore, reduced mTORC1 activity from upregulation of the mTORC1 inhibitors, REDD1 and SESTRIN2 (Fig 3F–I), at 100 days is compatible with suppressed muscle mass gain. Moreover, at 200 days we detected an increase in 4E‐BP1 levels and a normal fraction of phospho‐4E‐BP1. Restored 4EBP1 phosphorylation at 200 days could be due to partial mTORC1 reactivation through increased free amino acid pool generated during autophagy, which would allow for minimal anabolism to occur concomitantly with catabolism at late stage of myopathy. A similar regulation was also described in fasting *Drosophila*, where TORC1 acute down‐regulation at the onset of fasting was followed by a partial reactivation, which was dependent on autophagy induction (Jouandin *et al*, 2022). Interestingly, reduced mTORC1 activity promotes autophagic degradation and suppresses anabolism, thus lowering energy demand, a potentially necessary adaptation for the survival of OXPHOS defective muscle. As an alternative interpretation, upregulation of LC3II and p62 at 200 days of age could reflect a block in autophagy, which could at least in part be explained by the re‐activation of mTORC1 as shown by recovery of phospho‐4EBP1 levels (Fig 3B). Since ATF4 induces upregulation of REDD1 and SESTRIN2 implicated in mTORC1 inhibition (Xu *et al*, 2020; Jang *et al*, 2021) and mediates 4E‐BP1 induction (Yamaguchi *et al*, 2008; Kang *et al*, 2017), we propose that a balance between the conflicting ATF4/mTORC1 signaling regulates catabolic/anabolic state and proteostasis of OXPHOS defective muscle.

Although mitochondrial dysfunction is associated with ISR^mt^, the underlying triggers remain uncertain. Tracing ISR^mt^ mechanisms *in vivo* can be challenging not only because of ISR tissue‐specificity and dependency on the severity of mitochondrial dysfunction (Croon *et al*, 2022) but also because dyshomeostasis driving ISR can be rapidly cleared through tissue rewiring and inter‐organ exchange. Increased asparagine levels (Fig 1B) (Chen *et al*, 2018) and highly upregulated ASNS (Fig 4F) suggest that different from proliferating myoblasts (Mick *et al*, 2020), ISR activation in COX10 KO muscle is not triggered by defective asparagine synthesis. Redox dyshomeostasis (Appendix Fig S5E and H) and early activation of compensatory mechanisms for NAD^+^ regeneration (Fig 6A) suggest that similar to non‐proliferating myotubes (Mick *et al*, 2020), energy and redox imbalance could be primary ISR drivers in COX10 KO muscle.

COX10 KO mouse and MERRF muscle share profound alterations in lipid metabolism. Despite upregulation of muscle β‐oxidation enzymes, lipid profiles strongly suggest that β‐oxidation is impaired. In particular, marked accumulation of 3‐OH fatty acids and 3‐OH acyl carnitines suggest suppressed HADH activity which likely arises from the diminished availability of mitochondrial NAD^+^, a co‐substrate of HADH. Notably, plasma 3‐OH acyl carnitines and 3‐OH fatty acids are among the new validated markers for MELAS patients that strongly correlate with the disease severity (Sharma *et al*, 2021). Accumulation of 3‐OH fatty acid species was also detected in a mouse model of Ndufs4 deficiency (Leong *et al*, 2012) and in isolated mitochondria treated with respiratory chain inhibitors (Jin *et al*, 1992), suggesting that this metabolic signature attributable to NAD^+^/NADH redox imbalance can be shared by genetically diverse forms of mitochondrial myopathy. Therefore, 3‐OH fatty acid species elevated in muscle and plasma could be disease biomarkers potentially useful for the clinical management and testing of novel therapies for mitochondrial disorders. In addition, these markers justify therapeutic approaches targeting the NADH/NAD^+^ imbalance (Russell *et al*, 2020).

Increased fatty acid levels in plasma (from WAT lipolysis) and impaired fatty acid β‐oxidation in muscle result in accumulation of lipids droplets in both COX10 and MERRF muscle potentially contributing to the myopathic phenotype. The ectopic accumulation of lipids in non‐adipose tissue is known to negatively affect cells, tissues, and organs that store it. Specifically, a mouse model of muscle lipid overload, the muscle‐specific hLPL‐mouse (Levak‐Frank *et al*, 1995), shows skeletal muscle mass reduction, ultrastructural damage, impairment in regeneration, and severe myopathy (Tamilarasan *et al*, 2012). Similarly, human lipid storage disorders (LSDs) lead to intramuscular lipid accumulation and impaired mitochondrial bioenergetics causing progressive myopathy (Debashree *et al*, 2018) and lipid accumulation has been previously described in skeletal muscle of MERRF patients (Munoz‐Malaga *et al*, 2000). Moreover, recent meta‐analysis of clinical data from multiple cohorts of mitochondrial patients revealed that increased energy expenditure (hypermetabolism) is the most common disease feature which prevents accumulation of body fat and contributes to shortened patient's lifespan (Sturm *et al*, 2023). According to this study, ISR activation induced by OXPHOS defects with the increased extracellular secretion of metabokines produces a negative energy balance with reduced adiposity. Therefore, our investigation of the ISR‐mediated starvation‐like response leading to COX10 KO muscle and WAT wasting sheds new light on the systemic muscle‐WAT regulation and could guide the development of treatments to preserve adipose stores.

Strong upregulation of two mitochondrial Acyl‐CoA thioesterases in COX10 KO muscle, ACOT2 and ACOT9, may serve to terminate fatty acid oxidation allowing them to exit mitochondria, thereby preventing accumulation of toxic fatty acids. Therefore, it is likely that the muscle buildup of 3‐OH lipids results from decreased activity of HADH accompanied by the coordinated action of Enoyl‐CoA hydratase (ECH) and ACOTs (Fig 5B). The FGF21‐induced WAT lipolysis and the upregulation of muscle ACOT, in the presence of OXPHOS impairment and NAD^+^ limitation, is a likely mechanistic explanation for the age‐dependent buildup of muscle lipid droplets. In addition, increased valine‐derived catabolic intermediate β‐HIB (Appendix Fig S2Bb), a paracrine regulator free to cross mitochondrial and plasma membranes (Jang *et al*, 2016), could further promote muscle fatty acids uptake and lipid accumulation.

Evidence that mitochondrial NAD^+^ is limited in COX10 KO muscle comes from activation of the mitochondrial glutamate‐P5C‐proline pathway via upregulation of two consecutive enzymes in this pathway, P5CS and PYCR. In this mode of glutamate utilization, increased NADH oxidation by PYCR would provide mitochondrial NAD^+^, in partial compensation for defective OXPHOS. Of note, upregulation of P5CR and PYCR1 has been previously reported in another mouse model of mitochondrial myopathy (muscle‐specific COX15 KO mouse; Dogan *et al*, 2018), and we also detect upregulated PYCR1 in MERRF muscle, suggesting that proline synthesis is a common metabolic adaptation in mitochondrial myopathies. Activation of mitochondrial DT‐diaphorase detected in COX10 KO muscle also contributes to matrix NAD^+^ supply. Nevertheless, accumulation of glutamate/acetyl‐glutamate and 3OH‐fatty acids/3OH‐acylcarnitines suggests that, despite the utilization of alternative pathways of NAD^+^ regeneration, such as proline synthesis and DT‐diaphorase, the NAD^+^ matrix pool remains inadequate to fully support glutamate and fatty acid oxidation in COX10 KO and MERRF muscle.

Since leptin is produced and released by adipocytes, plasma leptin levels depend on fat mass. Therefore, low levels of plasma leptin at late disease stage in COX10 KO mice could result from the severe loss of fat deposits. Interestingly, at earlier disease stages, plasma leptin levels are normal, but sLepR, which interferes with leptin signaling, is increased (Appendix Fig S7B). This finding suggests that in early stages of myopathy, low leptin signaling may be mediated by circulating sLepR synthesized by the liver, as previously described in fasting and dietary protein restriction (Cohen *et al*, 2005; Yamada *et al*, 2019).

Decreased leptin levels have been linked to altered mitochondrial metabolism of the adipose tissue and previously described in patients with multiple symmetrical lipomatosis (MSL) caused by mutant mitofusin 2 (Rocha *et al*, 2017) and mouse models of primary adipose tissue pathology (Vernochet *et al*, 2012; Becker *et al*, 2018). Here, we show that OXPHOS dysfunction affecting muscle specifically can cause secondary adipose tissue metabolic changes, resulting in low plasma leptin levels.

Low plasma leptin stimulates the HPA axis, activating GC synthesis and release. Consistently, we find increased GC levels in plasma from both MERRF patients and COX10 KO mice, with similar increases observed in mouse muscle and liver (Fig 7E–G). As a major regulator of energy homeostasis, GCs rapidly mobilize energy stored in the form of carbohydrates, fats, and proteins. While adaptive during acute stress (e.g., infections and fasting), chronically elevated GCs lead to a profound depletion of energy stores, muscle atrophy (Braun & Marks, 2015), and dyslipidemia (Lee *et al*, 2014). GC‐induced dysmetabolic effects are remarkably similar to the metabolic changes in COX10 KO mice and MERRF patients. Therefore, chronic GC elevation in mitochondrial diseases could be a fundamental contributor to the metabolic response that leads to muscle protein breakdown and lipid mobilization. To investigate the role of GCs in muscle and fat wasting, we treated COX10 KO mice with the GC receptor (GR) antagonist RU‐486. A moderate improvement in muscle strength in RU‐486 COX10 KO‐treated mice suggests that GCs contribute to muscle weakness. RU‐486 treatment reduces fat store depletion and plasma carnitines accumulation. However, GR inhibition does not improve the ISR‐induced FGF21 signaling that controls the chronic catabolic state. These findings are suggestive of a multi‐layered regulation of COX10 KO muscle proteostasis, which limit the therapeutic potential of RU‐486. In addition, it is possible that the treatment with RU‐486 at 50 mg/kg/day inhibited GC signaling only partially, and that dose optimization may be necessary for a stronger effect on the myopathy.

In conclusion, our results indicate that mouse and human skeletal muscle subjected to chronic OXPHOS defects implement similar patterns of energy and redox pathway rewiring at the expense of metabolic homeostasis. Metabolic dyshomeostasis, resulting in a buildup of amino acids, fatty acids, and ketone bodies can contribute to severe and possibly fatal acidosis (Danhauser *et al*, 2015). The muscle metabolic adaptations are part of inter‐organ crosstalk coordinated by autocrine and endocrine effects of myokines and hormones, which take place in distinct phases (Fig 7Q). First, OXPHOS defects result in activation of the ISR^mt^ with increased FGF21 synthesis, secretion, and induction of ATF4. This is followed by a second phase, when an increased translation of specific metabolic enzymes involved in maintaining muscle energy homeostasis occurs in parallel to the inhibition of mTORC1 and protein synthesis. During this phase, FGF21 signals to liver and WAT, affecting lipolysis and leptin metabolism. In the third phase, low leptin signaling activates the HPA axis, inducing hyper‐corticosteronemia, which acts synergistically with FGF21 to cause adipose stores depletion and muscle lipid accumulation. These findings provide novel insights into the mechanisms of mitochondrial myopathies and suggest therapeutic strategies aimed at maintaining metabolic homeostasis. These could include dietary supplementation to increase the availability of oxidative amino acids which could be further potentiated by preventing catabolic signaling, for example, by inhibiting GC‐mediated responses.

## Materials and Methods

### Patients

Skeletal muscle and blood samples from patients and controls were collected and stored at the Fondazione Policlinico Universitario Agostino Gemelli IRCCS, Rome, Italy. The study was approved by the Ethics Committee of the Università Cattolica del Sacro Cuore (Rome, Italy; ID: 3754). Informed consent was obtained from all human subjects and the experiments conformed to the principles set out in the WMA Declaration of Helsinki and the Department of Health and Human Services Belmont Report. Biopsy of deltoid muscle and blood collection were performed after 8 h of fasting, for diagnostic purpose, at symptomatic stage, at the Fondazione Policlinico Universitario Agostino Gemelli IRCCS. Patients did not follow special diets. All patients underwent extensive clinical and radiological evaluations. Patients' and controls' information can be found in Appendix Table S1.

### Mouse model

Homozygous COX10^Flx^ mice, a generous gift from Dr. Francisca Diaz (University of Miami), were crossed with muscle‐Cre mice (*Myl1* ^*tm1(cre)Sjb*^/J, RRID:IMSR_JAX:024713) to generate the muscle‐specific COX10 KO mice. The phenotype of these mice has been described previously (Diaz *et al*, 2005). Only male mice were utilized for our experiments and data analysis, unless otherwise indicated. Calf (gastrocnemius and soleus) muscle, liver, hypothalamus, and plasma were collected from all animals at the same time of the day (10–11 am). All animal procedures were conducted in accordance with protocols approved by the Weill Cornell Research Animals Resource Center (RARC, protocol number 0610‐549A).

### Treadmill test

Endurance was measured weekly as time to fatigue on a conventional treadmill running task. The treadmill was equipped with a motivational grid (Columbus Instruments). After three training sessions, mice were placed on the treadmill at a fixed 5% incline with a speed starting at 5 m/min increasing incrementally by 1 m every 3 min up to 11 m/min and then and by 1 m every 1 min, as previously described (Castro & Kuang, 2017). Time of fatigue was recorded once mice failed to maintain pace.

### Muscle strength

Forepaw muscle strength was measured using a digital grip‐strength meter (Columbus Instruments). Animals were trained to grasp a horizontal grasping ring. The testing was repeated three consecutive times at 15 min intervals and the average was recorded (Meyer *et al*, 1979).

### Food intake

Mice were housed individually for 1 week to monitor body weight and food intake.

### Bodipy staining

Unfixed frozen muscle tissue was cryo‐sectioned at 10 μm, stained with 1 mg/ml BODIPY 493/503 (Invitrogen; Cat#D3922) for 30 min and washed in PBS three times for 10 min (Spangenburg *et al*, 2011). Slides were imaged on a Leica TCS SP5 confocal laser‐scanning microscope using identical parameters for CTL and COX10 KO sections.

### ORO staining and COX/SDH histochemistry

Deltoid muscle specimen was collected by open biopsy under local anesthesia from MERRF patients and Control person with no histological, histochemical, and immunohistochemical abnormalities. A fragment of the fresh biopsy was snap‐frozen in isopentane pre‐cooled in liquid nitrogen and stored at −80°C. Cryosections were stained with Oil red O and COX/SDH histochemistry using standard procedures (Ross, 2011; Mehlem *et al*, 2013; Alston *et al*, 2021).

### Quantification of leptin and soluble leptin receptor

Plasma leptin and sLepR concentrations were determined using the mouse/rat Leptin ELISA kit (R&D Systems; United States; MOB00B) and mouse Leptin R ELISA kit (Invitrogen; United States; EM48RB), respectively, according to manufacturer's instructions.

### Quantification of FGF21 and GDF15

Mouse plasma FGF21 and GDF15 concentrations were determined using the mouse/rat ELISA kits (R&D Systems; United States; MF2100 and MGD150, respectively) and following the manufacturer's instructions. Serum FGF21 concentration in MERRF and Controls was measured using Simple Plex cartridges and Ella apparatus (ProteinSimple, San Jose, CA, USA) according to the manufacturer's instructions.

### Gene expression analysis

Total RNA was extracted from snap‐frozen liver and muscle in Trizol reagent (Thermofisher #15596026) using the RNeasy Mini Kit (Qiagen #74104). RNA concentration was measured with NanoDrop1000 Spectrophotometer (Thermofisher) and integrity was checked in agarose gel electrophoresis. Extracted RNA (500 ng) was used to prepare 3′RNAseq libraries using the Lexogen QuantSeq 3′mRNA‐Seq Library Prep Kit forward (FWD) for Illumina. Libraries were sequenced with single‐end 86 bps on an Illumina NextSeq500 sequencer (Cornell Genomics Facility). Raw sequence reads were processed using the BBDuk program in the BBMap package. Trimmed reads were aligned to the mouse genome assembly GRCm38.p6 using the STAR aligner (version 2.5.0a). SAM files were converted to BAM to read overlapping reads per gene using HTSeq‐count (version 0.6.1; Anders *et al*, 2015). The R package DESeq2 (version 1.30.0; Love *et al*, 2014) was used to obtain both normalized and variance stabilized counts between CTL and COX10 KO animals.

### Protein analysis

#### SDS‐PAGE

Skeletal muscle and liver were rapidly dissected, snap‐frozen in liquid nitrogen, and kept at −80°C for western blot analysis. Tissues (approximately 20 mg) were pulverized in liquid nitrogen, with porcelain mortar and pestle (CoorsTek) placed on dry ice and suspended in RIPA buffer (500 μl) supplemented with protease inhibitors (Sigma‐Aldrich, 11697498001) and phosphatase inhibitors (100×, NaF 100 mM, Na_3_VO_4_ 100 mM, Pyrophosphate 100 mM, Imidazole 200 mM, pH 7). After 30 min incubation on ice, tissue homogenate was centrifuged at 15,000 *g* for 20 min at 4°C and supernatant was collected. Protein concentration was determined using the DC Protein Assay (BioRad, #5000112). For electrophoresis, proteins (50 μg) were suspended in Laemmli buffer (BioRad, #1610737) containing β‐mercaptoethanol, loaded on Any kD Mini‐Protean TGX protein gels (BioRad, #456‐8124), and separated by SDS‐PAGE. Proteins were transferred to PVDF membranes (BioRad, #1704275) using a Trans‐Blot^®^ Turbo‐transfer system (BioRad) and blocked in Intercept Blocking Buffer (LI‐COR Biosciences, #927‐60001). Membranes were probed overnight at 4°C with specific primary antibodies against the proteins of interest (anti‐GOT1 (RRID: AB_2113630), anti‐GOT2 (RRID: AB_2247898), anti‐OGDH (RRID: AB_2156759), anti‐ALT1 (RRID: AB_2230815), anti‐ALT2 (RRID: AB_2112098), anti‐GAPDH (RRID: AB_2107436), anti‐PCCA (RRID: AB_2878963), anti‐PCK1 (RRID: AB_2160031), anti‐MUT (RRID: AB_2147263), anti‐SUCLA2 (RRID: AB_2271200), anti‐PYCR1 (RRID: AB_2172878), anti‐PYCR2 (RRID: AB_2253344), anti‐P5CS (RRID:AB_2223896), anti‐MURF1/TRIM63 (RRID:AB_11232209), anti‐CPS1 (RRID: AB_2084238), anti‐ASS1 (RRID: AB_2060466), anti‐HADH (RRID: AB_10667408), anti‐ACOT2 (RRID: AB_2221535), anti‐ACOT9 (RRID: AB_2221519), anti‐ARG1 (RRID:AB_2881528), anti‐OTC (RRID: AB_2880528), anti‐ASL (RRID: AB_2882885), anti‐ATF4 (RRID: AB_2058598), anti‐MTHFD2 (RRID: RRID:AB_2147525), anti‐SESTRIN2 (RRID: AB_2185480), anti‐REDD1 (RRID: AB_2245711), anti‐ASNS (RRID: AB_2060119), anti‐β‐actin (RRID:AB_2687938) Proteintech; anti‐LC3B, (RRID: AB_796155) Sigma‐Aldrich; anti‐p62/SQSTM1 (RRID:AB_10011069), Novus/Abnova; anti‐ATROGIN 1 (RRID:AB_2104262), Novus Biologicals; anti‐TIM23 (RRID:AB_398754), BD Transduction Laboratories; anti‐α‐actin (RRID:AB_11143753), anti‐CS (RRID:AB_11143209), abcam; anti‐OAT (RRID:AB_263174), Bethyl Laboratories; anti‐COX1‐CoIV (RRID:AB_2532240), anti‐COX4‐Co‐IV (RRID:AB_2535839), anti‐70 kDa‐CoII (Cat # A11142—discontinued), Thermofisher; anti‐AGC1 (RRID: AB_2799655), anti‐Ph‐4E‐BP1 (RRID: AB_560835), anti‐4E‐BP1 (RRID: AB_331692), Cell Signaling; anti‐VDAC1, RRID:AB_2716304), Millipore Sigma. For protein detection, membranes were incubated for 1 h at room temperature with anti‐mouse (LI‐COR Biosciences, #926‐32212) or anti‐rabbit (LI‐COR Biosciences, # 926‐68073) IgG secondary fluorescent antibodies (1:15,000 dilution) and imaged using the LI‐COR CLx imaging system. Band intensities were quantified using Image Studio software (vs3.1, LI‐COR Biosciences). GAPDH, α‐actin, and β‐actin were used to normalize proteins of muscle and liver homogenates.

#### Blue native PAGE

Frozen muscle (20 mg) was homogenized by Tissue Tearor (BioSpec Products) in cold isolation buffer (225 mM D‐Mannitol, 75 mM Sucrose, 20 mM HEPES, 1 mM EGTA) supplemented with 1 mg/ml fatty acid‐free BSA (Sigma‐Aldrich, #A9647‐100G). Muscle homogenates were centrifuged twice at 1,500 *g* for 5 min at 4°C. The resulting supernatant was centrifuged at 15,000 *g* for 20 min at 4°C and the pellet suspended in isolation buffer without BSA was centrifuged again at 15,000 *g* for 20 min at 4°C. The crude mitochondrial pellet was suspended in 50 μl of isolation buffer without BSA and protein concentration was measured. Mitochondria (50 μg) were suspended in 50 μl buffer (1.5 M aminocaproic acid, 50 mM Bis‐tris, pH 7) containing 4 mg/ml lauryl maltoside (Invitrogen, #BN2008), incubated on ice for 5 min, and centrifuged at 20,000 *g* for 30 min at 4°C. The resulting supernatant was collected and 10x sample buffer (750 nM aminocaproic acid, 50 mM Bis‐Tris, 0.5 mM EDTA, pH 7), 5% Brilliant Blue G (Sigma‐Aldrich, #B0770‐5G) was added. Solubilized native proteins (25 μl) were separated on 4–16% NativePage gradient gel (Invitrogen). Electrophoresis was performed as described (Nijtmans *et al*, 2002). After overnight transfer of native protein onto PVDF membrane at 30 V, the membrane was incubated overnight at 4°C with monoclonal antibodies (Thermofisher) against 70 kDa of complex II and subunit I of complex IV. For immunodetection of mitochondrial complexes membrane was incubated for 1 h with anti‐mouse HRP‐conjugated secondary antibodies (Jackson Immunoresearch, #115‐035‐146) and imaged by chemiluminescence on a ChemiDoc system (BioRad).

### *In vivo*‐labeled metabolites tracing studies

COX10 KO and CTL mice were individually housed in metabolic cages with access to water for the duration of the experiment. For glutamate tracing, mice were injected intraperitoneally three times, 10 min apart, with 1.7 mg ^13^C5, ^15^N‐glutamate (^13^C5, 99%; ^15^N, 99%, Cambridge Isotope Laboratories) dissolved in 200 μl of 0.9% saline. Plasma and tissues were collected 10 min after the last injection and frozen in liquid nitrogen. For valine tracing, mice were injected intraperitoneally with 1 mg of ^13^C5, ^15^N‐valine (^13^C5, 99%; ^15^N, 99%, Cambridge Isotope Laboratories), ^12^C6, ^14^N‐leucine, and ^12^C6, ^14^N‐isoleucine dissolved in 200 μl of 0.9% saline. Plasma and tissues were collected 10 min after the injection and frozen in liquid nitrogen.

### RU‐486 treatment

Individually housed COX10 KO mice were administered 0.5% carboxymethylcellulose (CMC; Sigma 419273) or 50 mg/kg RU‐486 (Sigma M8046) dissolved in 0.5% CMC, daily through oral gavage (200 μl) starting at 43–50 days of age. Mice were evaluated for grip strength and body weight. Plasma and tissues were collected at the end of the treatment (100 and 130 days of age) and frozen in liquid nitrogen.

### AOA treatment

COX10 KO and CTL mice were intraperitoneally injected daily with 10 mg/kg of Aminooxyacetate (Sigma C13408, dissolved in PBS) or PBS, starting at 70 days of age. During the treatment, mice were evaluated for body weight and exercise endurance (by treadmill). *In vivo* tracing studies with [^13^C5, ^15^N]‐glutamate were performed at the end of the AOA treatment (130 days of age).

### Metabolomics

#### Reagents

LC–MS grade acetonitrile (ACN), isopropanol (IPA), and methanol (MeOH) were purchased from Fisher Scientific. High‐purity deionized water (ddH2O) was filtered from Millipore (18 OMG). OmniTrace glacial acetic acid and ammonium hydroxide were obtained from EMD Chemicals. Ammonium acetate, ammonium formate, and all other chemicals and standards were obtained from Sigma‐Aldrich in the best available grade.

#### Metabolite extraction

Tissue samples were washed with ice‐cold PBS, followed by metabolite extraction using −70°C 80:20 methanol:water (LC–MS grade methanol, Fisher Scientific). The tissue–methanol mixture was subjected to bead‐beating for 45 s using a Tissuelyser cell disrupter (Qiagen). Extracts were centrifuged for 5 min at 18500 x *g* to pellet insoluble protein and supernatants were transferred to clean tubes. The extraction procedure was repeated two additional times and all three supernatants were pooled, dried in a Vacufuge (Eppendorf), and stored at −80°C until analysis. The methanol‐insoluble protein pellet was solubilized in 0.2 M NaOH at 95°C for 20 min and protein was quantified using a BioRad DC assay. On the day of metabolite analysis, dried cell extracts were reconstituted in 70% acetonitrile at a relative protein concentration of 4 μg/ml, and 4 μl of this reconstituted extract was injected for LC/MS‐based targeted and untargeted metabolite profiling.

#### LC/MS metabolomics platform for untargeted metabolite profiling

Tissue extracts were analyzed by LC/MS as described previously (Chen *et al*, 2018), using a platform comprised of an Agilent Model 1290 Infinity II liquid chromatography system coupled to an Agilent 6550 iFunnel time‐of‐flight MS analyzer. Chromatography of metabolites utilized aqueous normal phase (ANP) chromatography on a Diamond Hydride column (Microsolv). Mobile phases consisted of (A) 50% isopropanol, containing 0.025% acetic acid, and (B) 90% acetonitrile containing 5 mM ammonium acetate. To eliminate the interference of metal ions on chromatographic peak integrity and electrospray ionization, EDTA was added to the mobile phase at a final concentration of 6 μM. The following gradient was applied: 0–1.0 min, 99% B; 1.0–15.0 min, to 20% B; 15.0 to 29.0, 0% B; 29.1 to 37 min, 99% B. Raw data were analyzed using MassHunter Profinder 8.0 and MassProfiler Professional (MPP) 15.1 software (Agilent technologies). Student's *t*‐tests (*P* < 0.05) were performed to identify significant differences between groups.

Plasma metabolites were extracted by the addition of 1 part plasma to 20 parts 70% acetonitrile in ddH_2_O (vol:vol). The mixture was briefly vortexed and then centrifuged for 5 min at 16,000 *g* to pellet precipitated proteins. An aliquot of the resulting extract (3 μl) was subjected to LC/MS untargeted metabolite profiling in positive and negative ion modes.

#### Tandem LC–MS/MS analysis of glucocorticoids

Tandem LC–MS/MS consisting of Agilent 6460A triple quadrupole mass spectrometer and Agilent 1290‐infinity LC system was used to separate and detect glucocorticoids (GC) isomers (corticosterone and cortisone) in mouse samples. The GCs were separated on an Agient Zorbax SB‐Aq column (1.8 μm, 2.1× 100 mm) reversed phase column and detected by positive ion multiple reaction monitoring (MRM) with an injection volume of 4 μl and column temperature of 30°C. Mobile phase solvent A contained 99% H_2_O, 1% ACN, 0.1% formic acid, and 1 mM ammonium formate, and solvent B contained 99% ACN, 1% H_2_O, 0.1% formic acid, and 1 mM ammonium formate. The following LC gradient was applied: 0–1.0 min, 0% B; 1.0–10.0 min, to 98% B; 10.0 to 15.0, 100% B; 15.1 to 23 min, 0% B. Corticosterone MRM spectra were acquired between 3 and 5 min with the primary transition of m/z 347.2 → 329.1(quantifier) at collision energy 14v and secondary transition of m/z 347.2 → 121.1(qualifier) at collision energy 30 V. Cortisone MRM spectra were acquired at fragmentor 172 V between 6 and8 min, using primary transition of m/z 361.3 → 163 (quantifier) at collision energy 26 V and secondary transition of m/z 362.1 → 145 (qualifier) at collision energy 38 V. The optimized source parameters were as follows: nebulizer, 26 psi; gas temperature, 320°C; gas flow, 5 l/min; sheath gas temperature, 350°C; sheath gas flow, 10 l/min; capillary voltage, 2,500 V; nozzle voltage, 300 V.

#### Metabolite structure specification

To ascertain the identities of differentially expressed metabolites (*P* < 0.05), LC/MS data were searched against an in‐house annotated personal metabolite database created using MassHunter PCDL manager 8.0 (Agilent Technologies), based on monoisotopic neutral masses (< 5 ppm mass accuracy) and chromatographic retention times. A molecular formula generator (MFG) algorithm in MPP was used to generate and score empirical molecular formulae, based on a weighted consideration of monoisotopic mass accuracy, isotope abundance ratios, and spacing between isotope peaks. A tentative compound ID was assigned when PCDL database and MFG scores concurred for a given candidate molecule. Tentatively assigned molecules were verified based on a match of LC retention times and/or MS/MS fragmentation spectra for pure molecule standards contained in a growing in‐house metabolite database.

#### Stable isotope tracing of [U‐^13^C, ^15^N] glutamate and [U‐^13^C, ^15^N] valine

An in‐house untargeted stable isotope tracing workflow (Chen *et al*, 2018, 2020, 2021) was employed to obtain all possible fates of tracer amino acids and quantitative information on the relative incorporation of tracer‐derived metabolites on the basis of the stable isotope labeling pattern. Isotope‐enriched metabolites were identified based on a pre‐curated database of isotopologues. Isotope enrichment was calculated after correcting the natural abundance contribution of heavy isotope in samples.

### Statistical analyses

In all the assays, the values are averages of at least three independent measurements. Error bars indicate standard deviation (S.D.) or standard error of the mean (S.E.M.). Statistically significant differences between two groups were estimated by unpaired two‐tailed Student's test with significance set at *P* < 0.05. For metabolomics data analysis, commercially available Agilent metabolomics software package was used to extract raw data. Peak areas of targeted metabolite‐extracted ion chromatograms were compared between groups. Missing values were excluded from statistical analyses. For heatmap generation, median normalized ion abundances were log2 transformed.

## Author contributions

**Nneka Southwell:** Data curation; formal analysis; investigation; writing – review and editing. **Guido Primiano:** Data curation; formal analysis; investigation; writing – review and editing. **Viraj Nadkarni:** Data curation; investigation; writing – review and editing. **Nabeel Attarwala:** Data curation; formal analysis; methodology. **Emelie Beattie:** Data curation; investigation; writing – review and editing. **Dawson Miller:** Data curation; methodology. **Sumaitaah Alam:** Data curation. **Irene Liparulo:** Data curation; formal analysis. **Yevgeniya Shurubor:** Data curation. **Maria Lucia Valentino:** Conceptualization; supervision. **Valerio Carelli:** Conceptualization; supervision. **Serenella Servidei:** Conceptualization; supervision. **Steven S Gross:** Conceptualization; formal analysis; funding acquisition; writing – review and editing. **Giovanni Manfredi:** Conceptualization; formal analysis; supervision; funding acquisition; writing – review and editing. **Marilena D'Aurelio:** Conceptualization; formal analysis; supervision; funding acquisition; validation; writing – original draft; writing – review and editing. **Qiuying Chen:** Conceptualization; formal analysis; supervision; funding acquisition; validation; writing – original draft; writing – review and editing.

## Disclosure and competing interests statement

The authors declare that they have no conflict of interest.

## Supporting information



Appendix S1

Source Data for Appendix

Source Data for Figure 1

Source Data for Figure 2

Source Data for Figure 3

Source Data for Figure 4

Source Data for Figure 5

Source Data for Figure 6

Source Data for Figure 7

## Data Availability

The dataset produced in this study is available in the following database: GEO: Gene Expression Omnibus GSE224780 (https://www.ncbi.nlm.nih.gov/geo/query/acc.cgi?acc=GSE224780).
