# Supplementary material for: A coordinated multiorgan metabolic response contributes to human mitochondrial myopathy
Source: EMBO Mol Med. 2023 May 24;15(7):e16951. doi: 10.15252/emmm.202216951 (PMC10331581; doi:10.15252/emmm.202216951)
Supplement: Supplementary file 1 — Appendix S1 [file EMMM-15-e16951-s003.pdf]

A coordinated multiorgan metabolic response contributes to human mitochondrial myopathy

Appendix

Contents

Appendix Tables **2**

Appendix Table S1: Patient and Control Information 2

Appendix Figures **3**

Appendix Figure S1: Differentially expressed metabolites and associated pathway enrichments in MERRF muscle and plasma 3

Appendix Figure S2: COX10 excised selectively in skeletal muscle results in stable complex IV deficiency, progressive myopathy and weight loss, age-dependent upregulation of key enzymes of amino acid oxidation, and preferred glutamate utilization 5

Appendix Figure S3: Phosphorylation and protein levels of the translation repressor 4EBP1 and protein levels of the autophagy marker p62 are age-dependent in COX10 KO muscle 10

Appendix Figure S4: ISRmt protein levels increase with age in COX10 KO muscle. Transcript levels of ISRmt genes are not upregulated in COX10 KO liver 11

Appendix Figure S5: Lipid metabolism and redox homeostasis are altered in OXPHOS defective muscle 12

Appendix Figure S6: Non-anaplerotic pathways of glutamate utilization are upregulated in COX10 KO muscle 14

Appendix Figure S7: Altered Leptin signaling and increase in plasma alanine and b-hydroxybutyrate occur at early disease stage in COX10 KO mice 16

Appendix Table

Appendix Table S1: Patient and CTL information.

Diagnosis	Specimen	Sex	Age at diagnosis	Mutation	COX-negative fibers	% Muscle Heteroplasmy
MERRF	muscle (10)	F(6), M(4)	40±13	m.8344A>G/*MT-TK*	+	80±5.8 (10)
	plasma/serum (9)	F(5), M(4)	43±6			
Healthy CTL	muscle (15)	F(8), M(7)	36±15	N/A	-	N/A
	plasma/serum (25)	F(11), M(14)	39±13			

Data are presented as Mean ± SD. F= females, M= Males. The number of individuals in each group is indicated in parenthesis. N/A=data not available. Presence (+) or absence (-) of COX-negative fibers.

Appendix Figures

Fig. S1

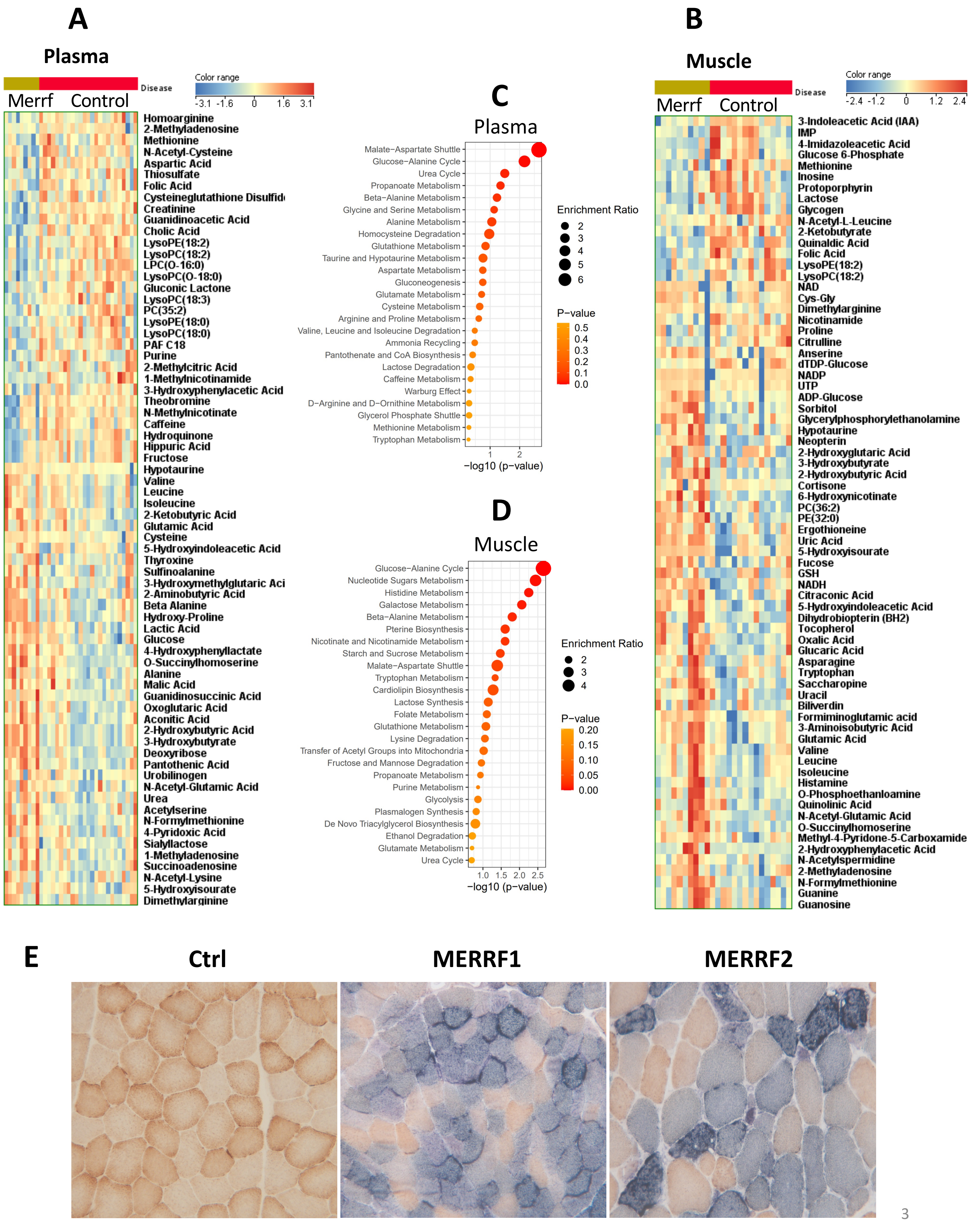

Appendix Figure S1: Differentially expressed metabolites and associated pathway enrichments in MERRF muscle and plasma.

Heatmap of the abundance of metabolites differentially expressed in MERRF plasma **(A)** and muscle **(B)** relative to control. Differentially expressed fatty acid and acylcarnitines (shown in Fig. 5 K and L) were excluded for heatmap generation and pathway enrichment analysis. Metabolite ion abundance was log2 transformed and normalized to the median using MassProfiler Professional 5.1 data analysis software (Agilent Technologies). Colors were based on the normalized ion abundance in the range of -3.1 to +3.1 for plasma and -2.4 to 2,4 for muscle, where blue denoted lowest abundance and red denoted highest abundance. Overview of the top 25 metabolic pathway enrichments in MERRF plasma **(C)** and muscle **(D)** generated by metabolite set enrichment analysis (MSEA, Metaboanalyst 5.0,). Enrichment ratio referred to the number of metabolite hits relative to the total number of corresponding pathway metabolites in Small Molecule Pathway Database (SMPDB). P value was calculated using hypergeometric test. **(E)** Sequential COX/SDH histochemical reactions in muscle sections showing numerous COX deficient fibers with mitochondrial proliferation (ragged-blue fibers). Images are taken at 10X magnification.

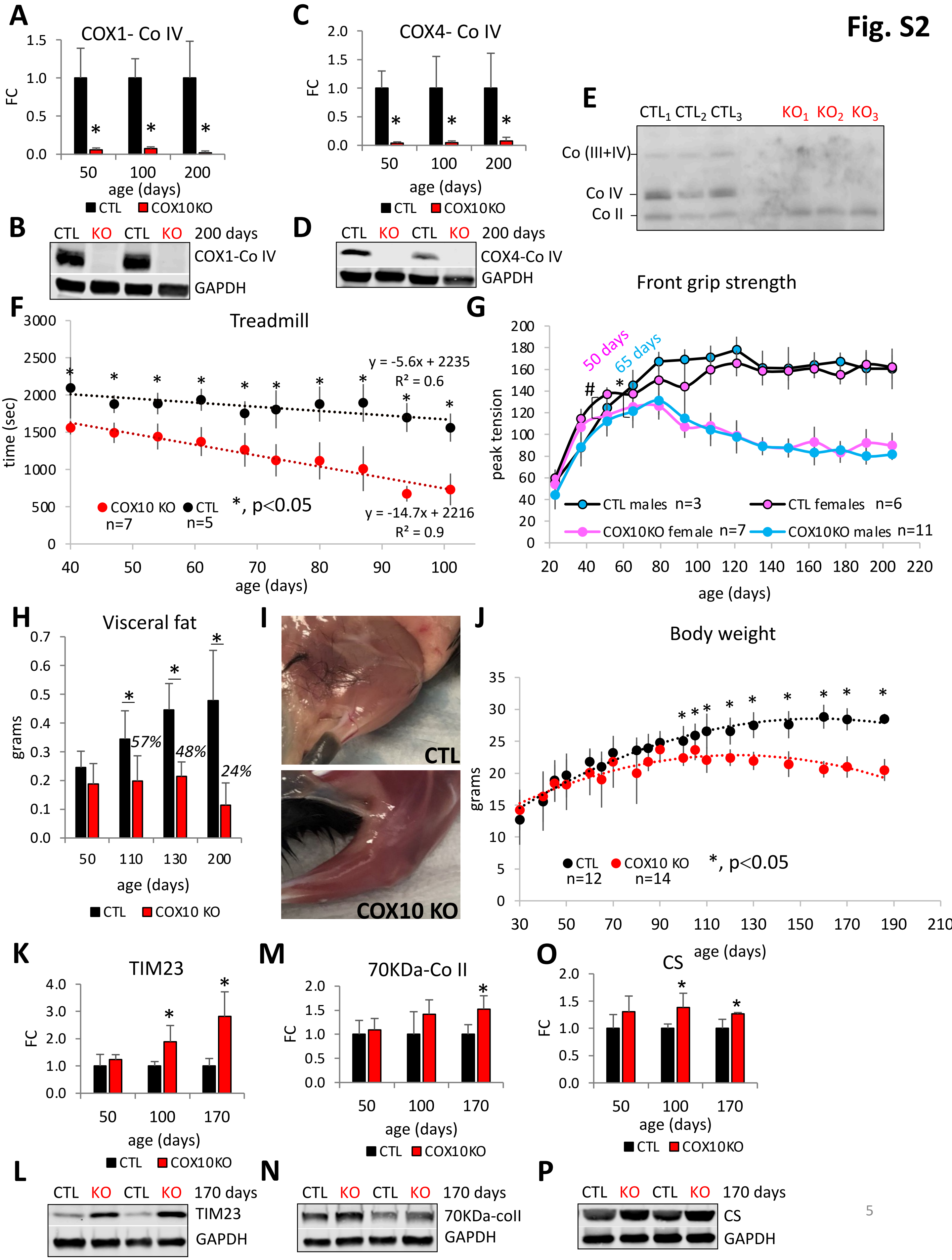

Fig. S2
A
COX1- Co IV
FC
age (days)
CTL
COX10KO
B
CTL KO CTL KO 200 days
COX1-Co IV
GAPDH
C
COX4- Co IV
FC
age (days)
CTL
COX10KO
D
CTL KO CTL KO 200 days
COX4-Co IV
GAPDH
E
CTL1 CTL2 CTL3 KO1 KO2 KO3
Co (III+IV)
Co IV
Co II
F
Treadmill
time (sec)
y = -5.6x + 2235
R² = 0.6
y = -14.7x + 2216
R² = 0.9
COX10 KO n=7
CTL n=5
*, p<0.05
age (days)
G
Front grip strength
peak tension
50 days
65 days
CTL males n=3
CTL females n=6
COX10KO female n=7
COX10KO males n=11
age (days)
H
Visceral fat
grams
57%
48%
24%
age (days)
CTL
COX10 KO
I
CTL
COX10 KO
J
Body weight
grams
CTL n=12
COX10 KO n=14
*, p<0.05
age (days)
K
TIM23
FC
age (days)
CTL
COX10KO
L
CTL KO CTL KO 170 days
TIM23
GAPDH
M
70KDa-Co II
FC
age (days)
CTL
COX10KO
N
CTL KO CTL KO 170 days
70KDa-coII
GAPDH
O
CS
FC
age (days)
CTL
COX10KO
P
CTL KO CTL KO 170 days
CS
GAPDH

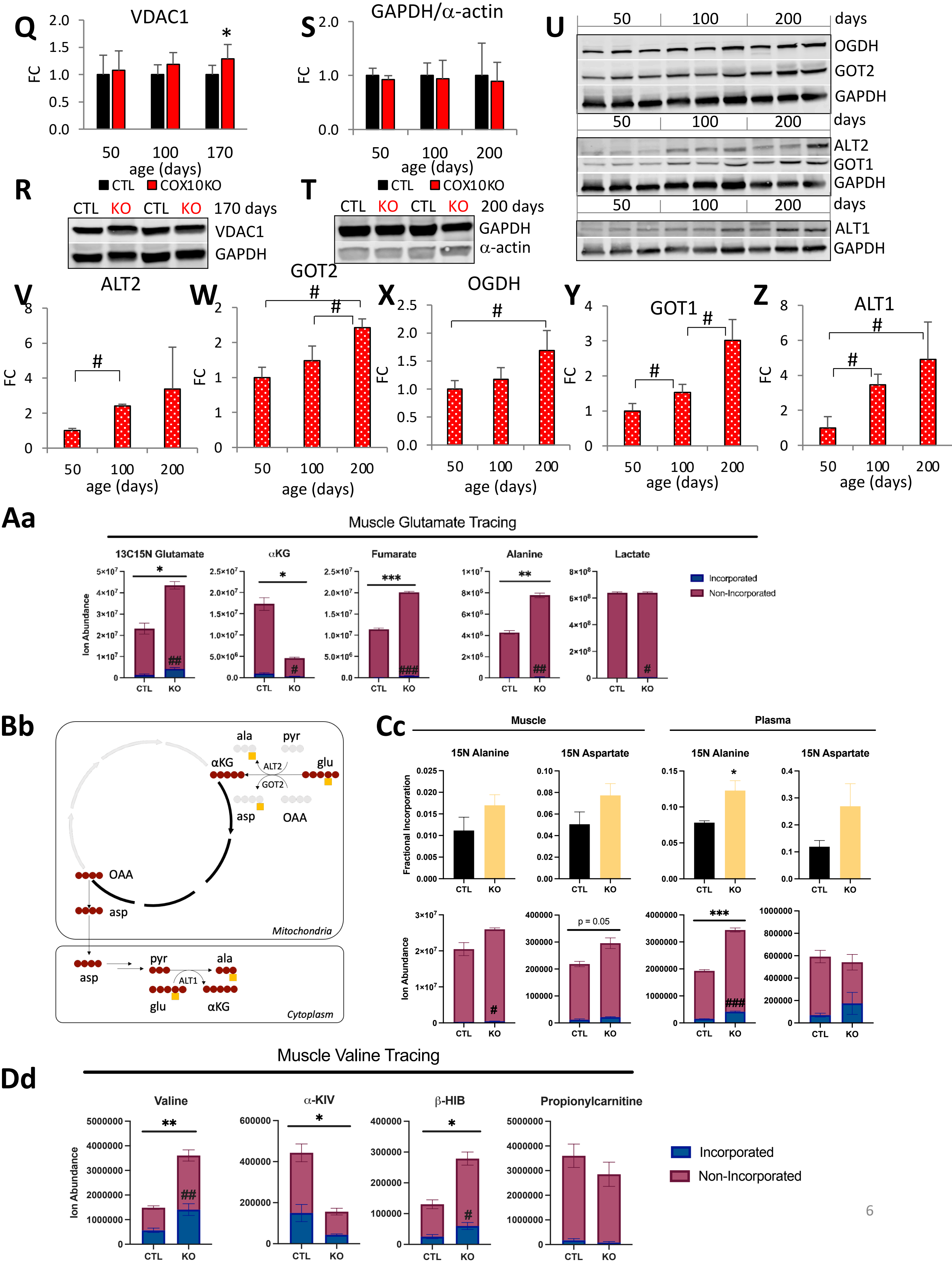

Q
VDAC1
FC
age (days)
R
CTL KO CTL KO 170 days
VDAC1
GAPDH
CTL COX10KO
S
GAPDH/α-actin
FC
age (days)
T
CTL KO CTL KO 200 days
GAPDH
α-actin
U
50 100 200 days
OGDH
GOT2
GAPDH
ALT2
GOT1
ALT1
V
ALT2
W
GOT2
X
OGDH
Y
GOT1
Z
ALT1
Aa
Muscle Glutamate Tracing
13C15N Glutamate
αKG
Fumarate
Alanine
Lactate
Ion Abundance
Incorporated
Non-Incorporated
Bb
ala
pyr
αKG
ALT2
GOT2
glu
asp
OAA
Mitochondria
pyr
ala
ALT1
glu
αKG
Cytoplasm
Cc
Muscle
Plasma
15N Alanine
15N Aspartate
Fractional Incorporation
Ion Abundance
p = 0.05
Dd
Muscle Valine Tracing
Valine
α-KIV
β-HIB
Propionylcarnitine
Ion Abundance
Incorporated
Non-Incorporated

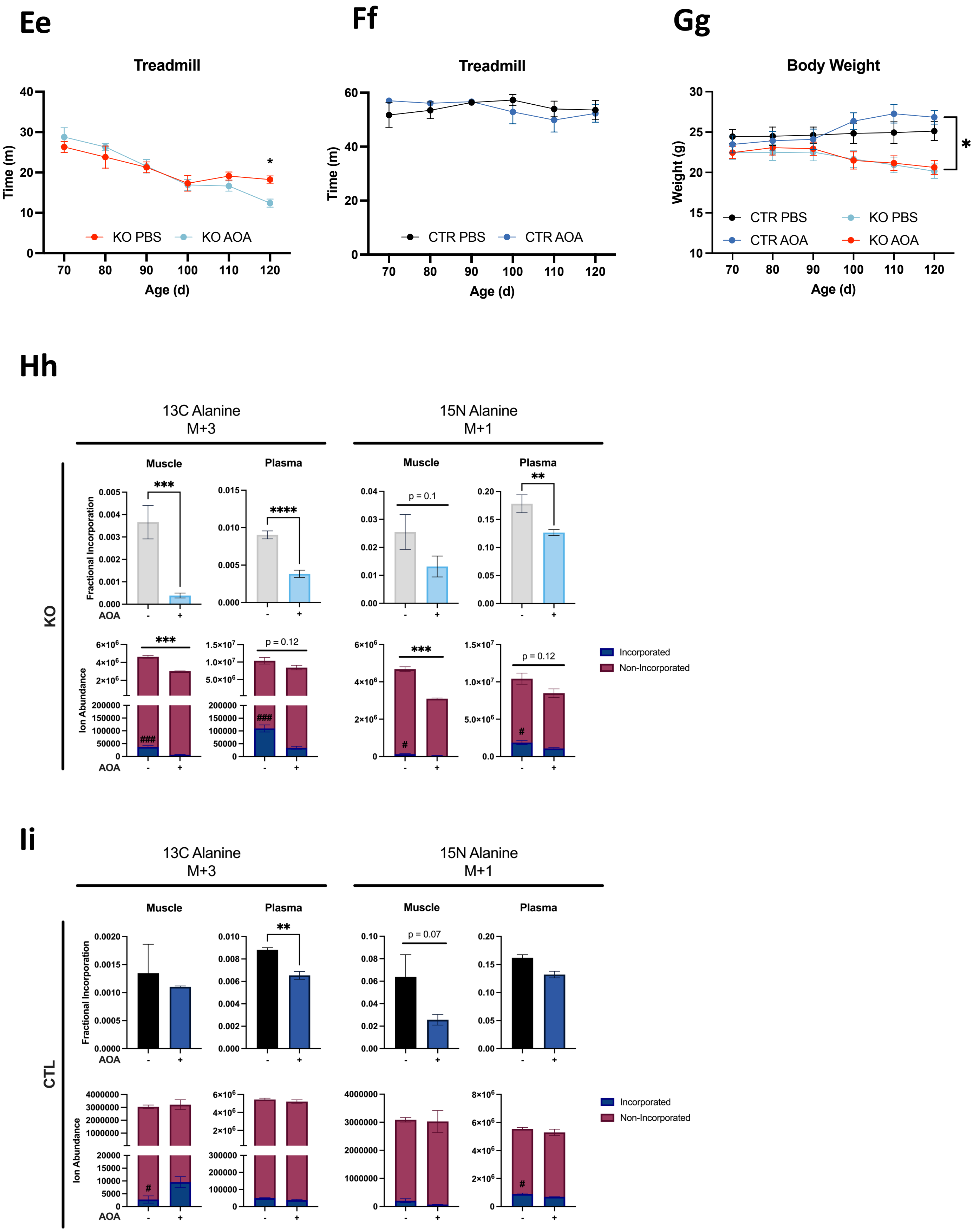
Ee
Treadmill
Time (m)
Age (d)
KO PBS
KO AOA
*
Ff
Treadmill
Time (m)
Age (d)
CTR PBS
CTR AOA
Gg
Body Weight
Weight (g)
Age (d)
CTR PBS
KO PBS
CTR AOA
KO AOA
*
Hh
13C Alanine
M+3
15N Alanine
M+1
KO
Muscle
Plasma
Fractional Incorporation
Ion Abundance
AOA
***
****
p = 0.1
**
p = 0.12
###
#
Incorporated
Non-Incorporated
Ii
13C Alanine
M+3
15N Alanine
M+1
CTL
Muscle
Plasma
Fractional Incorporation
Ion Abundance
AOA
**
p = 0.07
#
Incorporated
Non-Incorporated

Appendix Figure S2: COX10 excised selectively in skeletal muscle results in stable complex IV deficiency, progressive myopathy and weight loss, age-dependent upregulation of key enzymes of amino acid oxidation, and preferred glutamate utilization.

Age-dependent muscle protein levels of COX1 **(A)** and COX4 **(C)** subunits of Complex IV (CoIV) estimated by band densitometry normalized by GAPDH in COX10 KO (n=4 per age group) expressed relative to same age CTL (n=4 per age group) set at 1. Representative western blots of muscle lysates from 200 days old mice separated by denaturing SDS–PAGE and probed for COX1 and GAPDH **(B)** and COX4 and GAPDH **(D)**. **(E)** Western blot of mitochondria isolated from muscle of 200 days old CTL (n=3) and COX10 KO (n=3) mice, separated by BN–PAGE, probed for COX1 detecting Complex IV and supercomplex III+IV, and probed for 70KDa subunit of Complex II, detecting Complex II. **(F)** Age-dependent exercise intolerance of COX10 KO (n=7) vs. CTL (n=5) mice measured by treadmill. **(G)** Age-dependent muscle strength, measured by front grip strength of CTL females (n=6), COX10 KO females (n=7), CTL males (n=3), COX10 KO males (n=11). Data are presented as Mean ± SD. Muscle strength difference between COX10 KO females and CTL females starts at 50 days (#, $p< 0.05$) and between COX10 KO males and CTL males starts at 65 days (*, $p< 0.05$). **(H)** Visceral fat deposits of 50 days old COX10 KO (n=4) and CTL (n=4), 110 days old COX10 KO (n=5) and CTL (n=5), 130 days old COX10 KO (n=4) and CTL (n=6), and 200 days old COX10 KO (n=10) and CTL (n=15) . **(I)** CTL and COX10 KO mice hind limb at 200 days of age. **(J)** Age-dependent body weight of COX10 KO (n=14) and CTL (n=12) mice. In A, C, F, H, and J data are presented as Mean ± SD. *, $p<0.05$ COX10 KO vs. same age CTL. Age-dependent muscle levels of mitochondrial protein TIM23 **(K)**, 70KDa-CoII **(M),** CS **(O)**, and VDAC1 **(Q)** estimated by band densitometry normalized by GAPDH, and of cytosolic protein GAPDH **(S)** normalized by a-actin, in COX10 KO (n=4 per age group) expressed relative to same age CTL (n=4 per age group) set at 1. Data are presented as Mean ± SD. *, $p<0.05$ COX10 KO vs. same age CTL. Representative western blots of muscle lysates from 170-200 days old mice separated by denaturing SDS–PAGE and probed for TIM23 **(L)**, 70KDa-CoII **(N),** CS **(P)**, VDAC1 **(R),** GAPDH **(L, N, P, R, T)**, and a-actin **(T)**. **(U)** Western blots of muscle lysates from 50, 100, and 200 days old COX10 KO mice (n=3 per age group), separated by denaturing SDS–PAGE and probed for OGDH, GOT2, and GAPDH (top panel), ALT2, GOT1, and GAPDH (middle panel), ALT1 and GAPDH (bottom panel). Age-dependent COX10 KO muscle protein levels of ALT2 **(V)**, GOT2 **(W)**, OGDH **(X)**, GOT1 **(Y)**, and ALT1 **(Z)** estimated by band densitometry normalized by GAPDH, expressed relative to 50 days old COX10 KO set at 1. n=3 per age group. Data are presented as Mean ± SD. #, $p<0.05$ COX10 KO vs.COX10 KO at different age. **(Aa)** Muscle levels of total ($^{13}C^{15}N$-labeled in blue and unlabeled in purple) glutamate and total (^{13}C-labeled in blue and unlabeled in purple) αKG, fumarate, malate, alanine, and lactate. n=4 per genotype. Data are presented as Mean ± SEM. *, $p<0.05$; **, $p<0.01$; ***, $p<0.001$ on the bar, total metabolite abundance, CTL vs. COX10 KO. #, $p<0.05$; ##, $p<0.01$, labeled metabolite abundance, CTL vs. COX10 KO. **(Bb)** Schematic representation of [$^{13}C5$, ^{15}N]-glutamate transamination by GOT and ALT generating ^{15}N aspartate and ^{15}N alanine. Yellow square represents ^{15}N atom derived from [$^{13}C5$, ^{15}N]-glutamate. αKG, α-ketoglutarate; OAA, oxaloacetate; ala, alanine; asp, aspartate. **(Cc)** Fractional incorporation of ^{15}N alanine and ^{15}N aspartate in muscle and plasma and total (^{15}N-labeled in blue and unlabeled in purple) alanine and aspartate in muscle and plasma. Data are presented as Mean ± SEM. *, $p<0.05$, COX10 KO vs CTL. ***, $p<0.001$ on the bar, total metabolite abundance, CTL vs. COX10 KO. #, $p<0.05$; ###, $p<0.001$, labeled metabolite abundance, CTL vs. COX10 KO. **(Dd)** Muscle levels of total ($^{13}C^{15}N$-labeled in blue and unlabeled in purple) valine and total (^{13}C-labeled in blue and unlabeled in purple) α-KIV, β-HIB, and Propionylcarnitine. n=3 per genotype. Data are presented as Mean ± SEM. *, $p<0.05$; **, $p<0.01$ on the bar, total metabolite abundance, CTL vs. COX10 KO. #, $p<0.05$; ##, $p<0.01$, labeled metabolite abundance, CTL vs. COX10 KO.

Appendix Figure S2: COX10 excised selectively in skeletal muscle results in stable complex IV deficiency, progressive myopathy and weight loss, age-dependent upregulation of key enzymes of amino acid oxidation, and preferred glutamate utilization.

Age-dependent exercise intolerance of COX10 KO PBS (n=6) vs. COX10 KO AOA (n=7) mice **(Ee)** and of CTL PBS (5) vs. CTL AOA (6) mice **(Ff)**, measured by treadmill. Data are presented as Mean ± SEM. *, p< 0.05 COX10 KO PBS vs. COX10 KO AOA. **(Gg)** Body weight of COX10 KO PBS (n=6), COX10 KO AOA (n=7), CTL PBS (5), and CTL AOA (6) mice. Data are presented as Mean ± SEM. *, p< 0.05 COX10 KO PBS vs. CTL PBS starting at 110 days. Fractional incorporation of ^{13}C and ^{15}N alanine (**Hh**, top panel) and total alanine (**Hh**, bottom panel) in muscle and plasma of 130 days old COX10 KO PBS (n=5) and COX10 KO AOA (n=6) mice IP injected with [$^{13}C5$, ^{15}N]-glutamate. Fractional incorporation of ^{13}C and ^{15}N alanine (**Ii**, top panel) and total alanine (**Ii**, bottom panel) in muscle and plasma of 130 days old CTL PBS (n=4) and CTL AOA (n=5) mice IP injected with [$^{13}C5$, ^{15}N]-glutamate. In **Hh** and **Ii** data are presented as Mean ± SEM. **, p<0.01; ***, p<0.001 PBS vs AOA (top panels). #, p<0.05; ##, p<0.01; ###, p<0.001 labeled PBS vs. labeled AOA (bottom panels). *, p<0.05; ***, p<0.01; ***, p<0.001 total PBS vs total AOA (bottom panels)

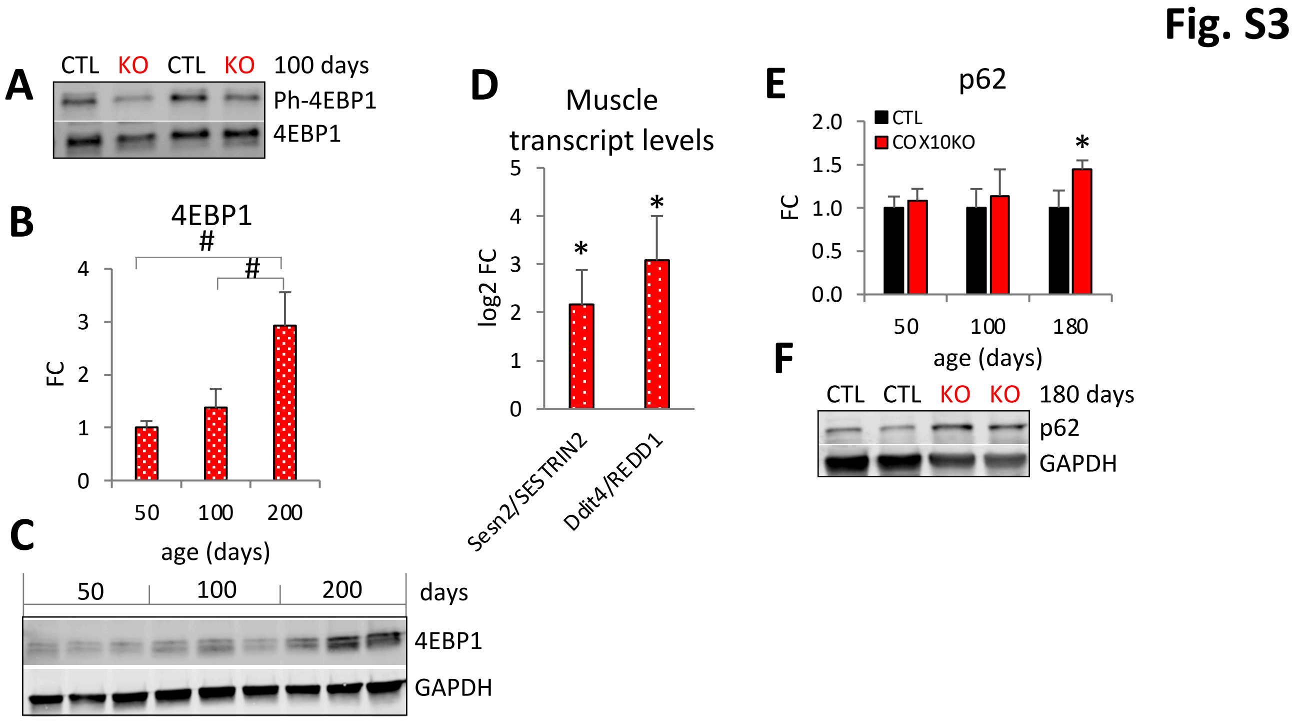

Appendix Figure S3: Phosphorylation and protein levels of the translation repressor 4EBP1 and protein levels of the autophagy marker p62 are age-dependent in COX10 KO muscle.
(A) Representative western blot of muscle lysates from 100 days old mice separated by denaturing SDS–PAGE and probed for Ph-4E-BP1 and for 4E-BP1 (relative to Fig. 3B). **(B)** Age-dependent protein levels of 4E-BP1 in COX10 KO muscle, estimated by band densitometry normalized by GAPDH expressed relative to 50 days COX10 KO set at 1. n=3 per age group. Data are presented as Mean ± SD. #, $p<0.05$ COX10 KO vs.COX10 KO at different age. **(C)** Western blots of muscle lysates from 50, 100, and 200 days old COX10 KO mice, separated by denaturing SDS–PAGE and probed for 4E-BP1 and GAPDH. **(D)** Muscle transcript levels of *Sens2* and *Ddit4* in 200 days old COX10 KO (n=6) expressed as log2 fold change relative to CTL (n=6). Data are presented as Mean ± SE. *, $p<0.05$ COX10 KO vs. CTL. **(E)** Age-dependent protein levels of p62 estimated by band densitometry normalized by GAPDH in COX10 KO (n=4 per age group) expressed relative to same age CTL (n=4 per age group) set at 1. Data are presented as Mean ± SD. *, $p<0.05$ COX10 KO vs. same age CTL. **(F)** Representative western blot of muscle lysates from 180 days old mice separated by denaturing SDS–PAGE and probed for p62 and GAPDH.

Fig. S4

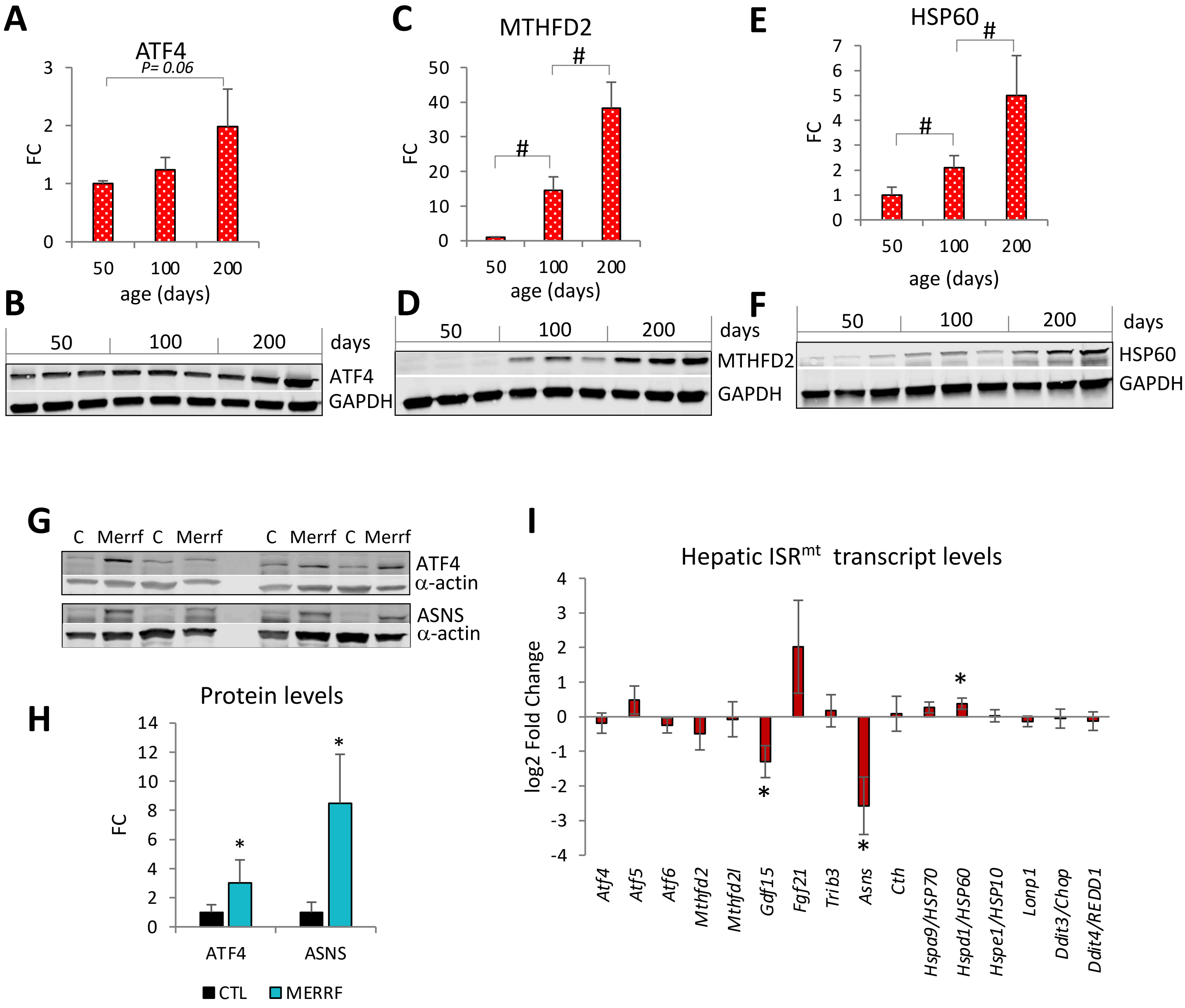

Appendix Figure S4: ISR^{mt} protein levels increase with age in COX10 KO muscle. Transcript levels of ISR^{mt} genes are not upregulated in COX10 KO liver.

Age-dependent COX10 KO muscle protein levels of ATF4 **(A)**, MTHFD2 **(C)**, and HSP60 **(E)**, estimated by band densitometry normalized by GAPDH, expressed relative to 50 days COX10 KO set at 1. n=3 per age group. Data are presented as Mean ± SD. #, p<0.05 COX10 KO vs. COX10 KO at different age. Western blots of muscle lysates from 50, 100, and 200 days old COX10 KO mice separated by denaturing SDS–PAGE and probed for ATF4 **(B)**, MTHFD2 **(D)**, and HSP60 **(F)** and GAPDH. **(G)** Western blots of MERRF and CTL muscle lysates separated by denaturing SDS–PAGE and probed for ATF4, ASNS, and α-actin. **(H)** ATF4 and ASNS protein levels estimated by band densitometry normalized by α-actin, in MERRF muscle (n=4) expressed relative to CTL muscle (n=4). Data are presented as Mean ± SD. *, p<0.05 MERRF vs. CTL. **(I)** Hepatic transcript levels of ISR^{mt} genes in 200 days old COX10 KO (n=6) expressed as log2 fold change relative to CTL (n=6). Data are presented as Mean ± SE. *, p<0.05 COX10 KO vs. CTL.

Fig. S5

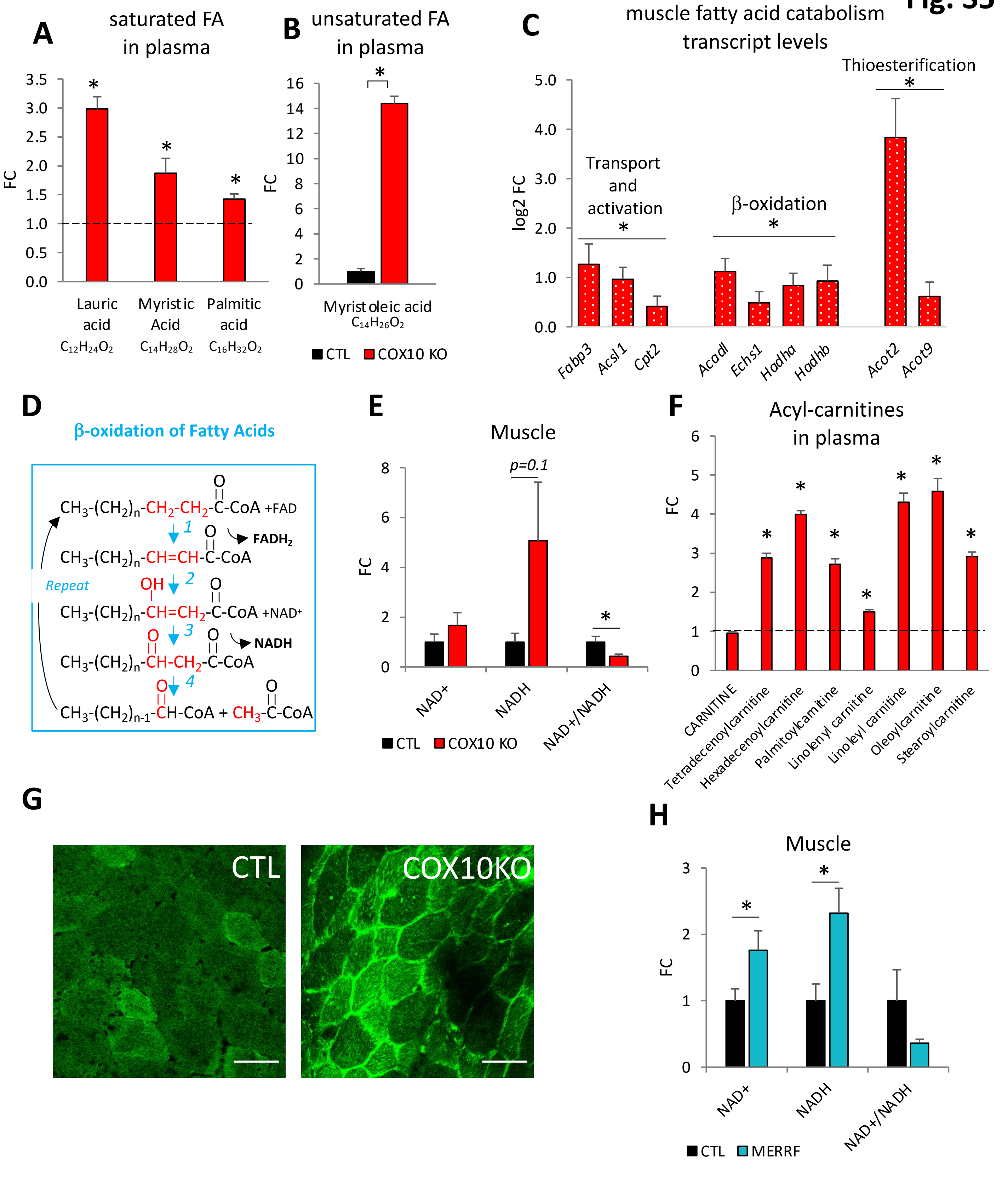
A
saturated FA in plasma
FC
Lauric acid
$C_{12}H_{24}O_2$
Myristic Acid
$C_{14}H_{28}O_2$
Palmitic acid
$C_{16}H_{32}O_2$
B
unsaturated FA in plasma
FC
Myristoleic acid
$C_{14}H_{26}O_2$
CTL
COX10 KO
C
muscle fatty acid catabolism transcript levels
log2 FC
Transport and activation
β-oxidation
Thioesterification
Fabp3
Acsl1
Cpt2
Acadl
Echs1
Hadha
Hadhb
Acot2
Acot9
D
β-oxidation of Fatty Acids
$CH_3-(CH_2)_n-CH_2-CH_2-C(=O)-CoA$ +FAD
FADH2
$CH_3-(CH_2)_n-CH=CH-C(=O)-CoA$
Repeat
$CH_3-(CH_2)_n-CH(OH)=CH_2-C(=O)-CoA$ +NAD+
NADH
$CH_3-(CH_2)_n-C(=O)H-CH_2-C(=O)-CoA$
$CH_3-(CH_2)_{n-1}-C(=O)H-CoA + CH_3-C(=O)-CoA$
E
Muscle
FC
p=0.1
NAD+
NADH
NAD+/NADH
CTL
COX10 KO
F
Acyl-carnitines in plasma
FC
CARNITINE
Tetradecenoylcarnitine
Hexadecenoylcarnitine
Palmitoylcarnitine
Linolenyl carnitine
Linoleyl carnitine
Oleoylcarnitine
Stearoylcarnitine
G
CTL
COX10KO
H
Muscle
FC
NAD+
NADH
NAD+/NADH
CTL
MERRF

Appendix Figure S5: Lipid metabolism and redox homeostasis are altered in OXPHOS defective muscle. Plasma levels of saturated **(A)** and unsaturated **(B)** fatty acids (FA) by LC-MS analysis in 200 days old COX10 KO (n=6) expressed relative to CTL (n=6) set at 1 (dashed line in A). Data are presented as Mean ± SEM. * p<0.05 COX10 KO vs. CTL. **(C)** Muscle transcript levels of fatty acid oxidation genes in 200 days old COX10 KO (n=6) expressed as log2 fold change relative to CTL (n=6). Data are presented as Mean ± SE. *, p<0.05 COX10 KO vs. CTL. *Fabp3*, fatty acid-binding protein 3; *Acsl1*, acyl-CoA synthetase long chain family member 1; *Cpt2*, carnitine palmitoyltransferase 2; *ACOT*, acyl-CoA thioesterase. **(D)** Steps of β-oxidation of fatty acids generating $FADH_2$, NADH, and acetyl-CoA and resulting in fatty acyl chain shortened by two carbons: 1. acyl CoA dehydrogenase (ACAD), 2. enoyl-CoA hydratase (ECH), 3. 3-hydroxyacyl-CoA dehydrogenase (HADH), 4. 3-ketoacyl-CoA thiolase (ACAT1). **(E)** Muscle levels of NADH, NAD^+, and NAD^+/NADH by LC-MS analysis in 200 days COX10 KO (n=6) expressed relative to CTL (n=6) set at 1. Data are presented as Mean ± SEM. * p<0.05 COX10 KO vs. CTL. **(F)** Plasma levels of acyl carnitines by LC-MS analysis in 200 days COX10 KO mice expressed relative to CTL value set at 1 (dashed line). n=6 per genotype. Data are presented as Mean ± SEM. * p<0.05 COX10 KO vs. CTL. **(G)** Image of calf muscle cross section stained for lipids with BODIPY (green) from 100 days old COX10 KO and CTL. Images are taken at 40X magnification (scale bar, 50 µm). **(H)** Muscle levels of NADH, NAD^+, and NAD^+/NADH by LC-MS analysis in MERRF (n=8) expressed relative to CTL (n=12) set at 1. Data are presented as Mean ± SEM. * p<0.05 MERRF vs. CTL.

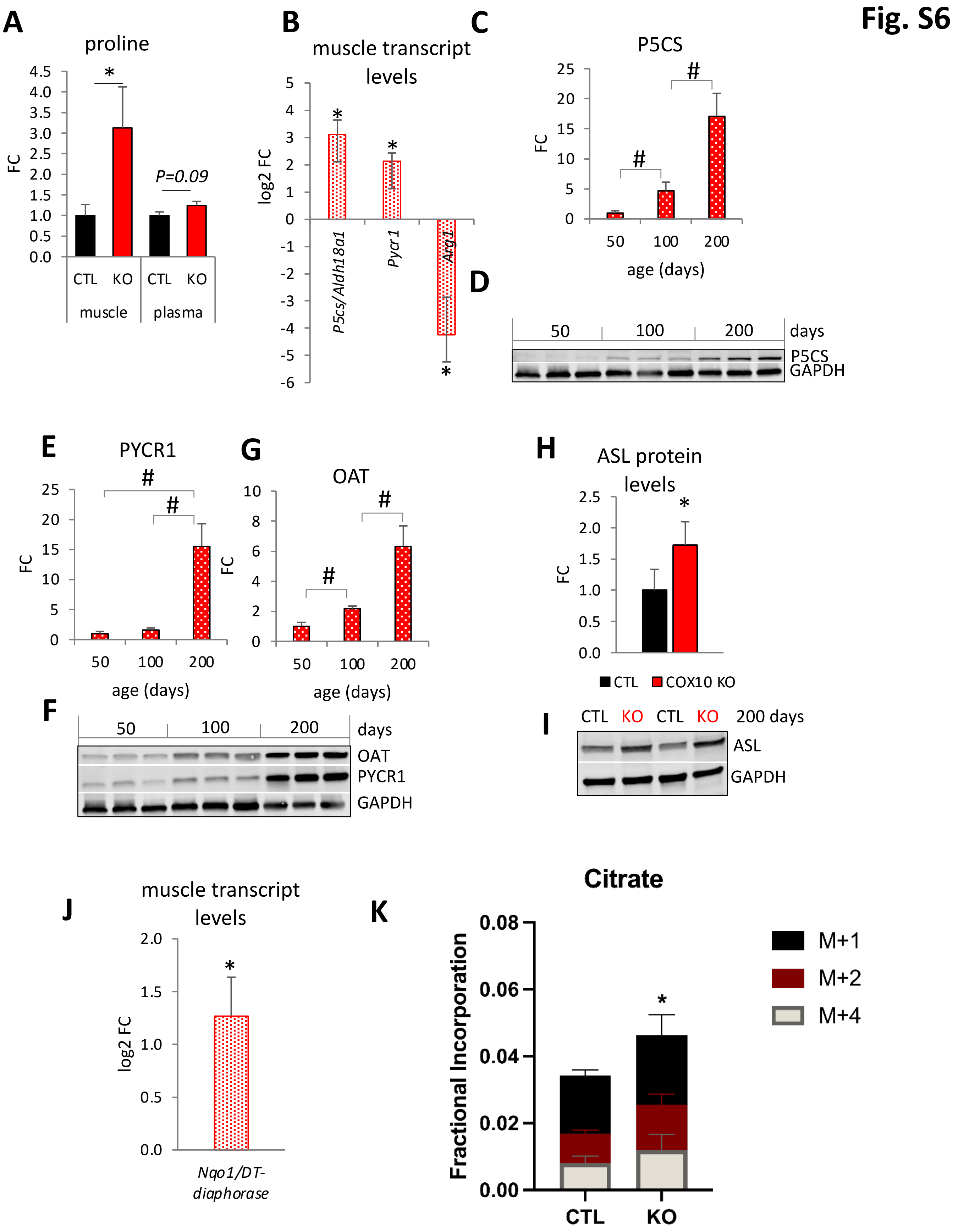
Fig. S6
A
proline
FC
4.5
4.0
3.5
3.0
2.5
2.0
1.5
1.0
0.5
0.0
*
P=0.09
CTL
KO
CTL
KO
muscle
plasma
B
muscle transcript levels
log2 FC
5
4
3
2
1
0
-1
-2
-3
-4
-5
-6
*
*
*
P5cs/Aldh18a1
Pycr1
Arg1
C
P5CS
FC
25
20
15
10
5
0
#
#
50
100
200
age (days)
D
50
100
200
days
P5CS
GAPDH
E
PYCR1
FC
25
20
15
10
5
0
#
#
50
100
200
age (days)
G
OAT
FC
10
8
6
4
2
0
#
#
50
100
200
age (days)
H
ASL protein levels
FC
2.5
2.0
1.5
1.0
0.5
0.0
*
CTL
COX10 KO
F
50
100
200
days
OAT
PYCR1
GAPDH
I
CTL
KO
CTL
KO
200 days
ASL
GAPDH
J
muscle transcript levels
log2 FC
2.0
1.5
1.0
0.5
0.0
*
Nqo1/DT-diaphorase
K
Citrate
Fractional Incorporation
0.08
0.06
0.04
0.02
0.00
*
CTL
KO
M+1
M+2
M+4

Appendix Figure S6: Non-anaplerotic pathways of glutamate utilization are upregulated in COX10 KO muscle.

(**A**) Muscle and plasma levels of proline by LC-MS analysis, in 200 days COX10 KO (n=6) expressed relative to CTL (n=6) set at 1. Data are presented as Mean ± SEM. * p<0.05 COX10 KO vs. CTL. **(B)** Muscle transcript levels of *P5cs, Pycr1*, and *Arg1* genes in 200 days old COX10 KO (n=6) expressed as log2 fold change relative to CTL (n=6). Data are presented as Mean ± SE. *, p<0.05 COX10 KO vs. CTL. Age-dependent COX10 KO muscle protein levels of P5CS **(C)**, PYCR1 **(E)**, and OAT **(G)**, estimated by band densitometry normalized by GAPDH expressed relative to 50 days COX10 KO set at 1. n=3 per age group. Data are presented as Mean ± SD. #, p<0.05 COX10 KO vs. COX10 KO at different age. Western blots of muscle lysates from 50, 100, and 200 days old COX10 KO mice, separated by denaturing SDS–PAGE and probed for P5CS and GAPDH **(D)** and for OAT, PYCR1, and GAPDH **(F)**. **(H)** Muscle protein levels of ASL in 200 days old COX10 KO (n=4) expressed relative to same age CTL (n=4) set at 1. Data are presented as Mean ± SD. *, p<0.05 COX10 KO vs. CTL. **(I)** Representative western blot of muscle lysates from 200 days old mice separated by denaturing SDS–PAGE and probed for ASL and GAPDH.

(J) Muscle transcript levels of *Nqo1* gene in 200 days old COX10 KO (n=6) expressed as log2 fold change relative to CTL (n=6). Data are presented as Mean ± SEM. *, p<0.05 COX10 KO vs. CTL

(K) Mass isotopologue analysis of citrate showing M+1, M+2, M+4 fractional incorporation in muscle. Data are presented as Mean ± SEM. *, p<0.05 COX10 KO vs. CTL.

Fig. S7

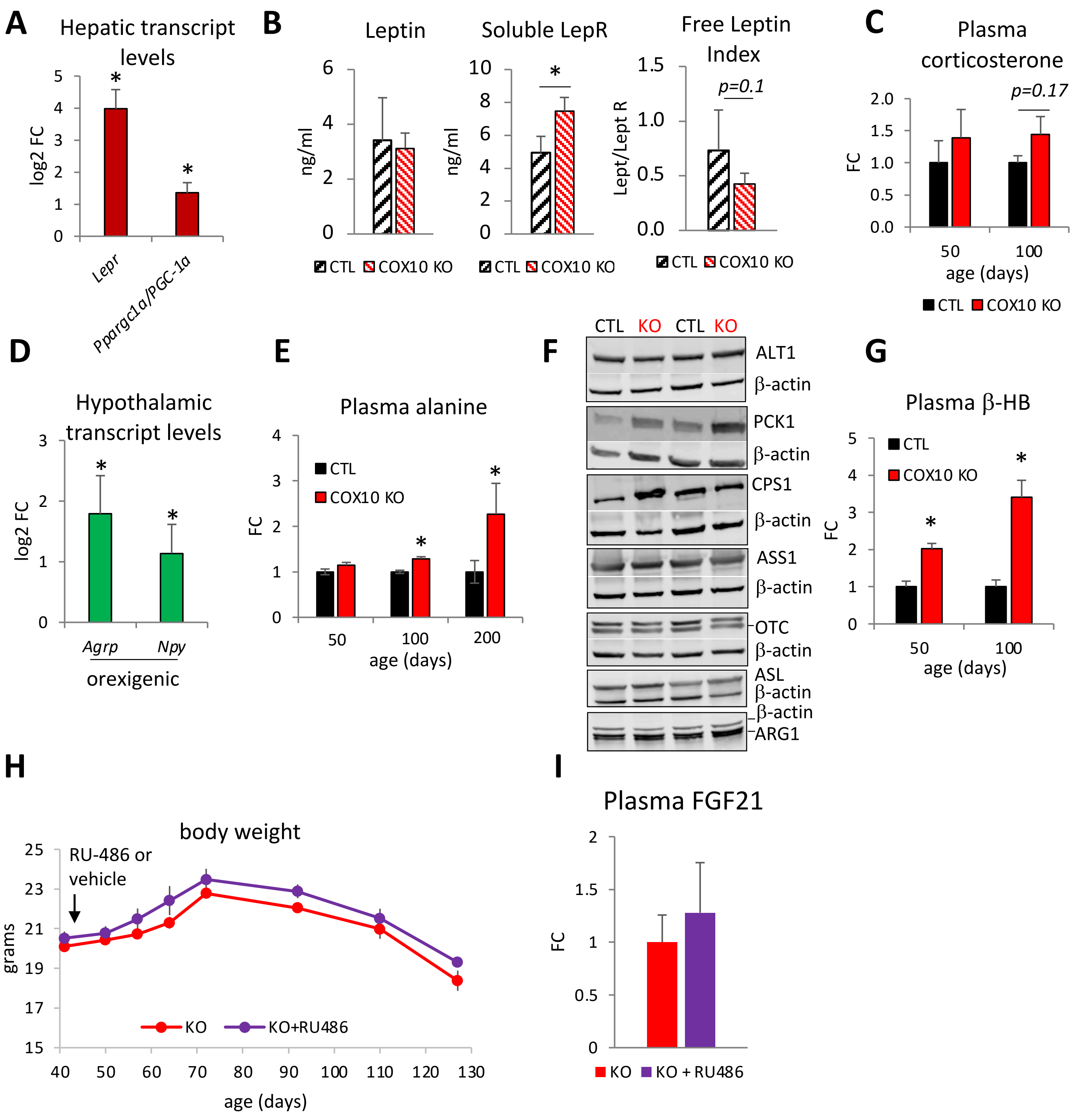

A
Hepatic transcript levels
log2 FC
Lepr
Ppargc1a/PGC-1a
B
Leptin
Soluble LepR
Free Leptin Index
ng/ml
Lept/Lept R
p=0.1
CTL
COX10 KO
C
Plasma corticosterone
p=0.17
FC
age (days)
D
Hypothalamic transcript levels
Agrp
Npy
orexigenic
E
Plasma alanine
F
CTL KO CTL KO
ALT1
β-actin
PCK1
CPS1
ASS1
OTC
ASL
ARG1
G
Plasma β-HB
H
body weight
RU-486 or vehicle
grams
KO
KO+RU486
I
Plasma FGF21
KO + RU486

Appendix Figure S7: Altered Leptin signaling and increase in plasma alanine and β-hydroxybutyrate occur at early disease stage in COX10 KO mice.

(A) Hepatic transcript levels of *Lepr, and PGC-1α* genes in 200 days old COX10 KO (n=6) expressed as log2 fold change relative to CTL (n=6). Data are presented as Mean ± SE. *, p<0.05 COX10 KO vs. CTL. **(B)** Plasma levels of Leptin, soluble Leptin Receptor (LepR), and Free Leptin Index by ELISA, in 100 days old CTL (n=4) and COX10 KO mice (n=4). Data are presented as Mean ± SD. *, p<0.05 COX10 KO vs. CTL. **(C)** Plasma levels of corticosterone by LC-MS/MS analysis in 50- and 100 days old COX10 KO (n=3 duplicate measurements per age group) and CTL (n=3 duplicate measurements per age group) expressed relative to same age CTL value set at 1. Data are presented as Mean ± SEM. **(D)** Hypothalamic transcript levels of *Agrp* and *Npy* genes in 200 days old COX10 KO (n=6) expressed as log2 fold change relative to CTL (n=6). Data are presented as Mean ± SE. *, p<0.05 COX10 KO vs. CTL. **(E)** Age-dependent plasma levels of alanine by LC-MS (n=6 per genotype) expressed relative to same age CTL value set at 1. Data are presented as Mean ± SEM. * p<0.05 COX10 KO vs. same age CTL. **(F)** Representative western blots of 200 days liver lysates separated by denaturing SDS–PAGE and probed for ALT1, PCK1, CPS1, ASS1, OTC, ASL, ARG1 and β-actin. **(G)** Age-dependent plasma levels of β-hydroxybutyrate (β-HB) by LC-MS (n=6 per genotype) expressed relative to same age CTL value set at 1. Data are presented as Mean ± SEM. * p<0.05 COX10 KO vs. same age CTL. **(H)** Age-dependent body weight of COX10 KO mice treated with RU-486 (KO+RU486, n=6) and vehicle (KO, n=4) mice. Data are presented as Mean ± SEM. The black arrow indicates the beginning of the treatment at 43 days. **(I)** Plasma levels of FGF21 in 100 days old COX10 KO mice treated with RU-486 (KO + RU486, n=6) and vehicle (KO, n=6) mice. Data are presented as Mean ± SEM.
