# Supplementary material for: A coordinated multiorgan metabolic response contributes to human mitochondrial myopathy
Source: EMBO Mol Med. 2023 May 24;15(7):e16951. doi: 10.15252/emmm.202216951 (PMC10331581; doi:10.15252/emmm.202216951)
Supplement: Supplementary file 3 — Source Data for Figure 1 [file EMMM-15-e16951-s006.zip › Figure 1/1H-I/a-actin.pdf]

Image ID: 0003398_01
Acquire Time: Dec 12, 2015 1:04:52 PM

Acquisition Information

#	Image ID	Acquire Time	Channels	Resolution	Intensities	Quality	Analysis	Image Name
1	0003398_01	Dec 12, 2015 1:04:52 PM	700 800	169um	Auto Auto	high	Manual	0003398_01

Image Display Values

Channel	Color	Minimum	Maximum	K
800	Gray Scale (Black on White)	0.650	119	0

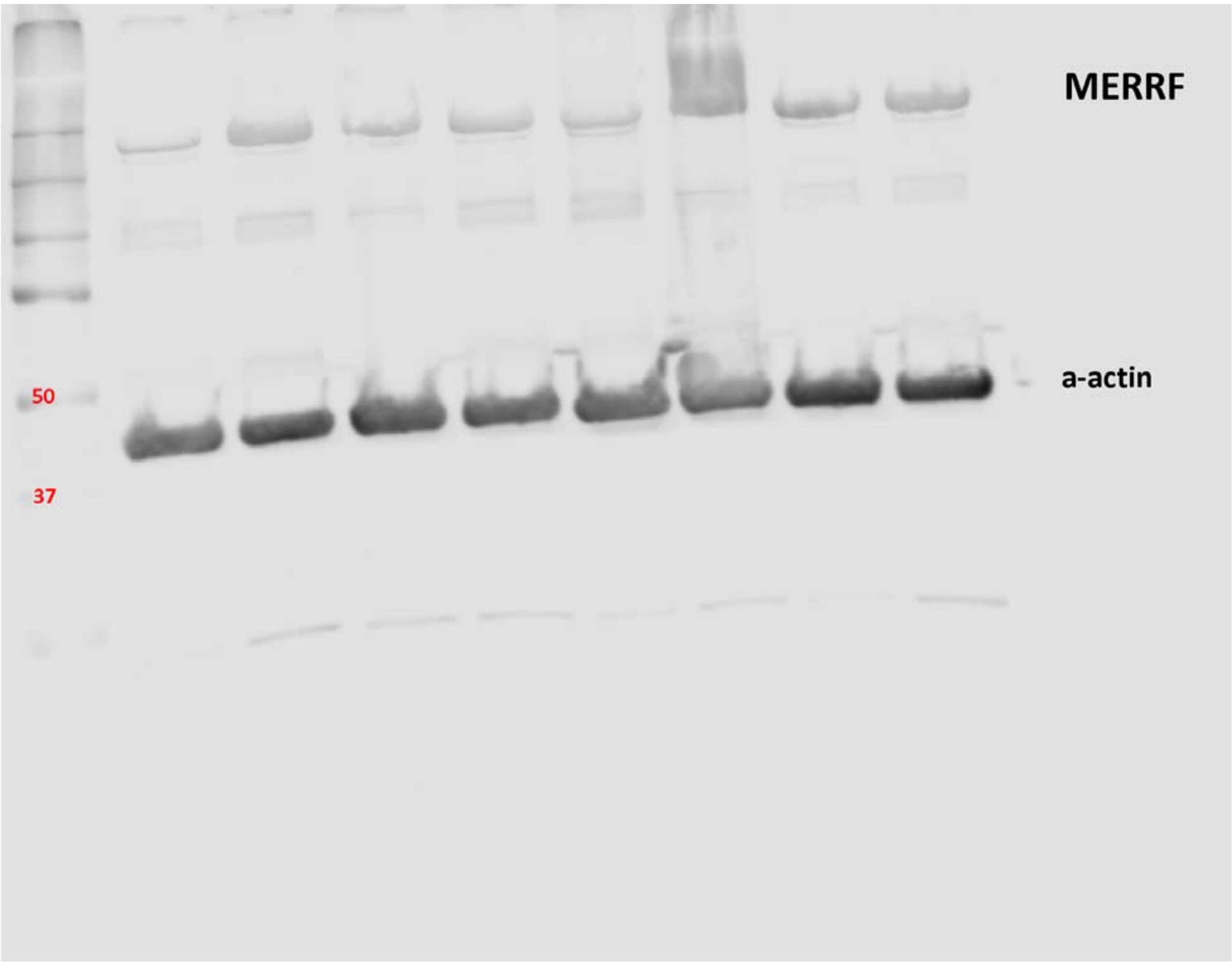

Acquisition Information (continued)

#	Comment	Image Modifications	Experiment
1	alfa-actine_1:2000_RG_WB12/04/15(3)		MERRF a-actin
