# Supplementary material for: A coordinated multiorgan metabolic response contributes to human mitochondrial myopathy
Source: EMBO Mol Med. 2023 May 24;15(7):e16951. doi: 10.15252/emmm.202216951 (PMC10331581; doi:10.15252/emmm.202216951)
Supplement: Supplementary file 3 — Source Data for Figure 1 [file EMMM-15-e16951-s006.zip › Figure 1/1H-I/ALT2.pdf]

Image ID: 0003378_01
Acquire Time: Dec 8, 2015 2:25:12 PM

Acquisition Information

#	Image ID	Acquire Time	Channels	Resolution	Intensities	Quality	Analysis	Image Name
1	0003378_01	Dec 8, 2015 2:25:12 PM	700 800	169um	Auto Auto	high	Manual	0003378_01

Image Display Values

Channel	Color	Minimum	Maximum	K
700	Gray Scale (Black on White)	1.34	5.38	0

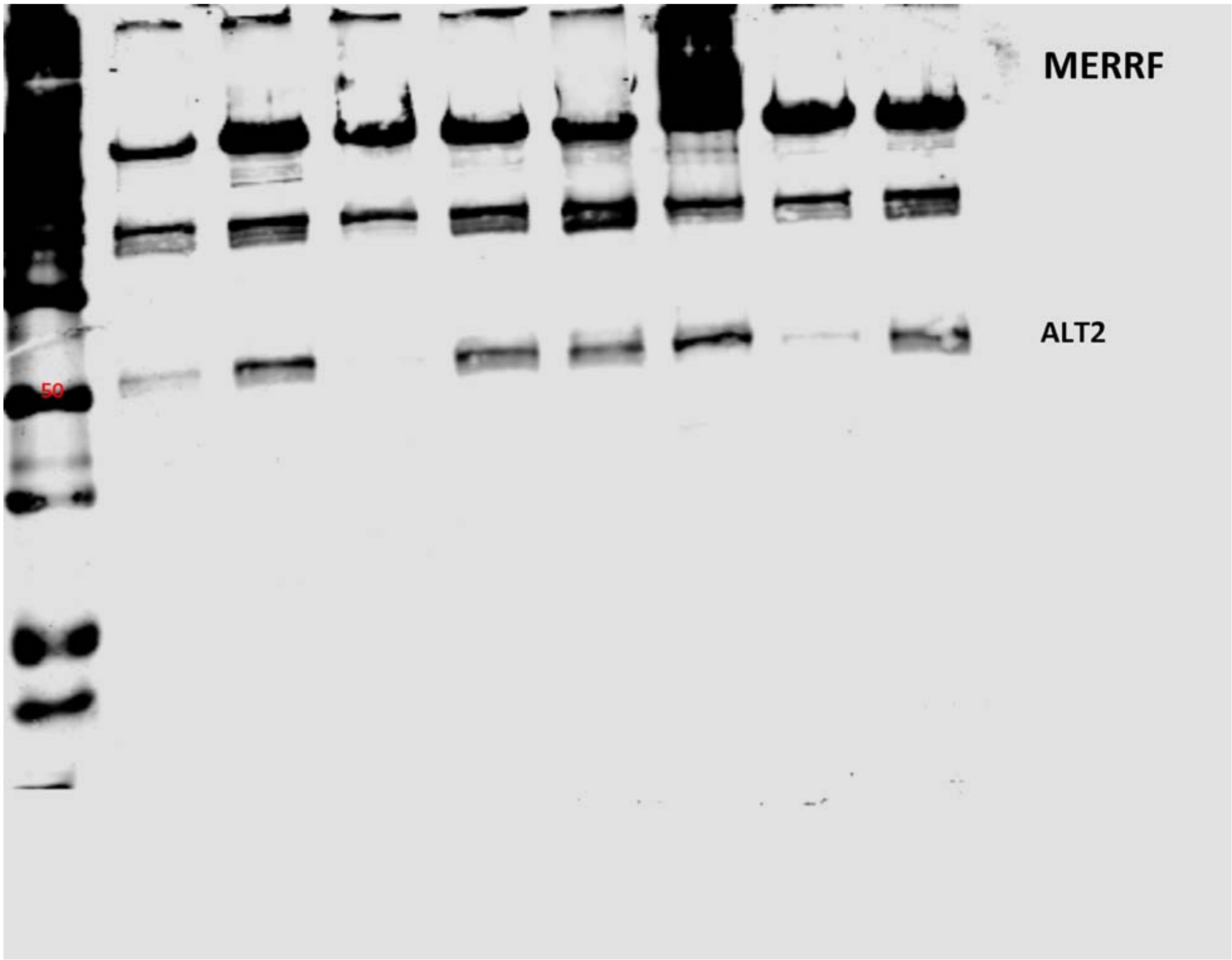

Acquisition Information (continued)

#	Comment	Image Modifications	Experiment
1	ALT2_1:250_RR_WB04/12/15(3)		
