# Supplementary figures and images for: A coordinated multiorgan metabolic response contributes to human mitochondrial myopathy
Source: EMBO Mol Med. 2023 May 24;15(7):e16951. doi: 10.15252/emmm.202216951 (PMC10331581; doi:10.15252/emmm.202216951)

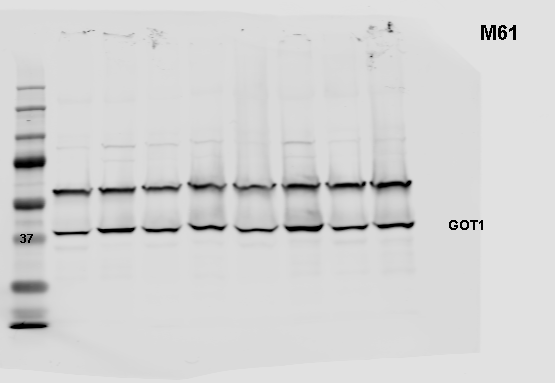

Supplement: Supplementary file 3 — Source Data for Figure 1 [file EMMM-15-e16951-s006.zip › Figure 1/1H-I/GOT1.pptx.tif]

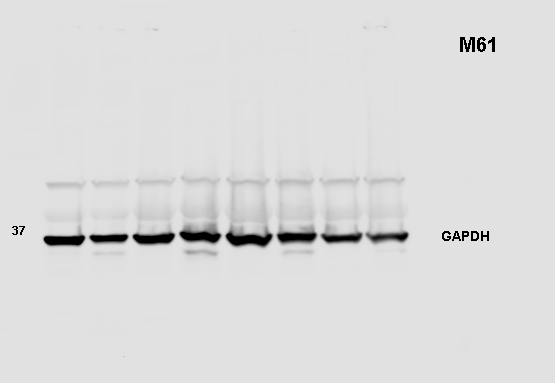

Supplement: Supplementary file 3 — Source Data for Figure 1 [file EMMM-15-e16951-s006.zip › Figure 1/1H-I/GAPDH -GOT1.pptx.tif]

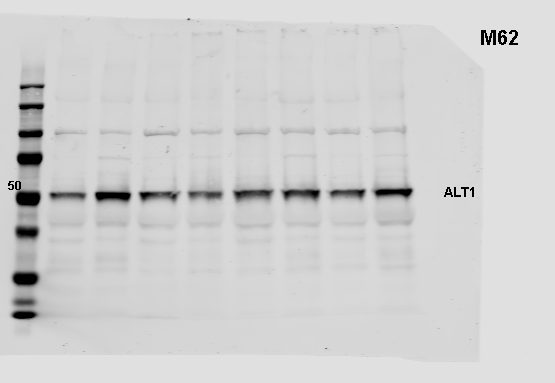

Supplement: Supplementary file 3 — Source Data for Figure 1 [file EMMM-15-e16951-s006.zip › Figure 1/1H-I/ALT1.pptx.tif]

Image Display Parameters

| Channel | Color                       | Minimum | Maximum | K |
|---------|-----------------------------|---------|---------|---|
| 700     | Gray Scale (Black on White) | 0.0159  | 38.1    | 0 |

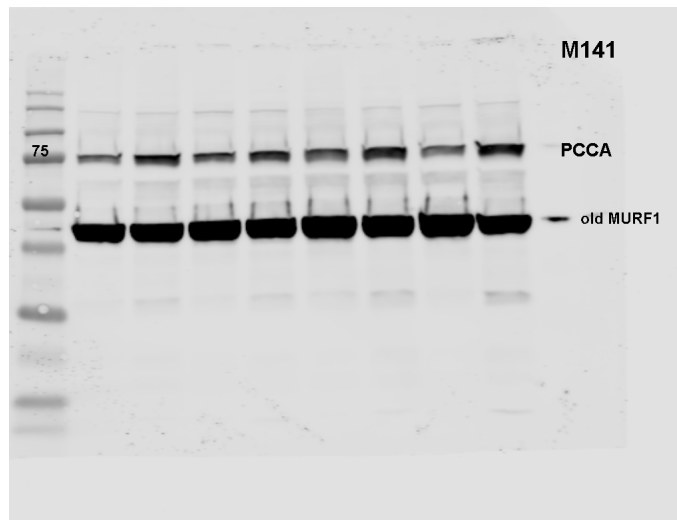

Supplement: Supplementary file 4 — Source Data for Figure 2 [file EMMM-15-e16951-s002.zip › Figure 2/2S-T/PCCA_2019-05-01.pdf]

Image Display Parameters

| Channel | Color                       | Minimum  | Maximum | K |
|---------|-----------------------------|----------|---------|---|
| 700     | Gray Scale (Black on White) | 0.000216 | 2.96    | 0 |

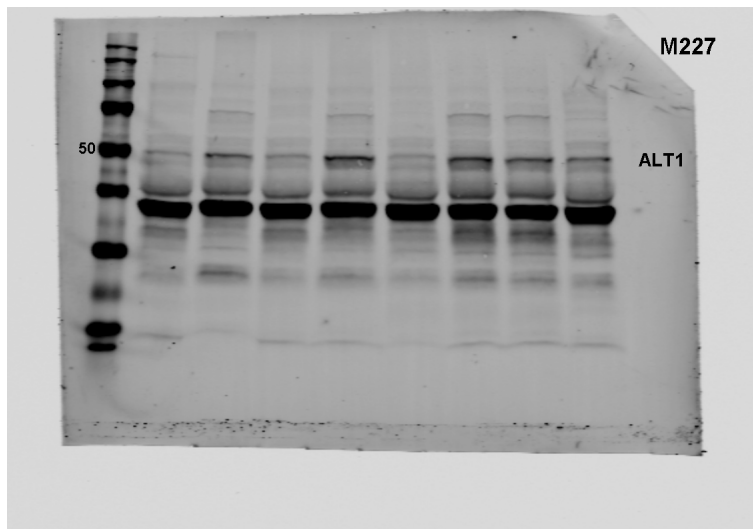

Supplement: Supplementary file 4 — Source Data for Figure 2 [file EMMM-15-e16951-s002.zip › Figure 2/2I-J/ALT1_2018-12-14.pdf]

Image Display Parameters

| Channel | Color                       | Minimum | Maximum | K |
|---------|-----------------------------|---------|---------|---|
| 800     | Gray Scale (Black on White) | 1.56    | 33.2    | 0 |

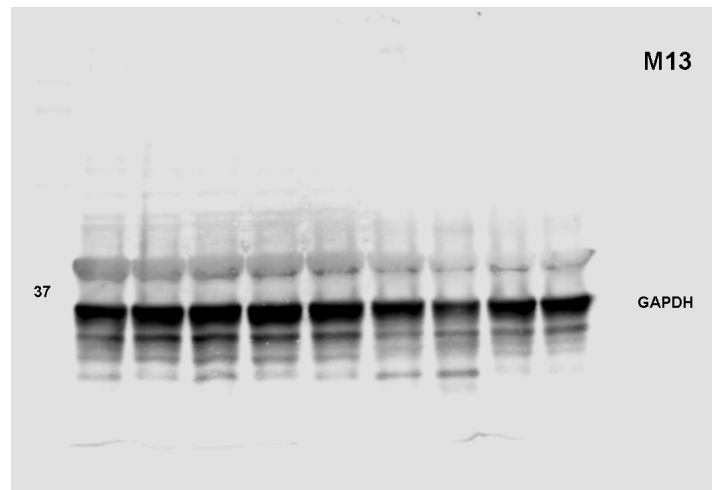

Supplement: Supplementary file 4 — Source Data for Figure 2 [file EMMM-15-e16951-s002.zip › Figure 2/2C-D/GAPDH_2019-04-30.pdf]

Image Display Parameters

| Channel | Color                       | Minimum | Maximum | K |
|---------|-----------------------------|---------|---------|---|
| 700     | Gray Scale (Black on White) | 4.34    | 17.4    | 0 |

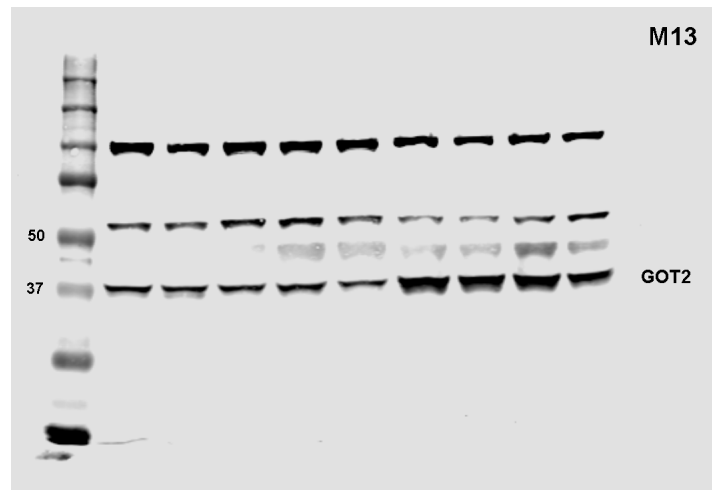

Supplement: Supplementary file 4 — Source Data for Figure 2 [file EMMM-15-e16951-s002.zip › Figure 2/2C-D/GOT2_2019-04-30.pdf]

Image Display Parameters

| Channel | Color                       | Minimum | Maximum | K |
|---------|-----------------------------|---------|---------|---|
| 700     | Gray Scale (Black on White) | 0.654   | 35.6    | 0 |

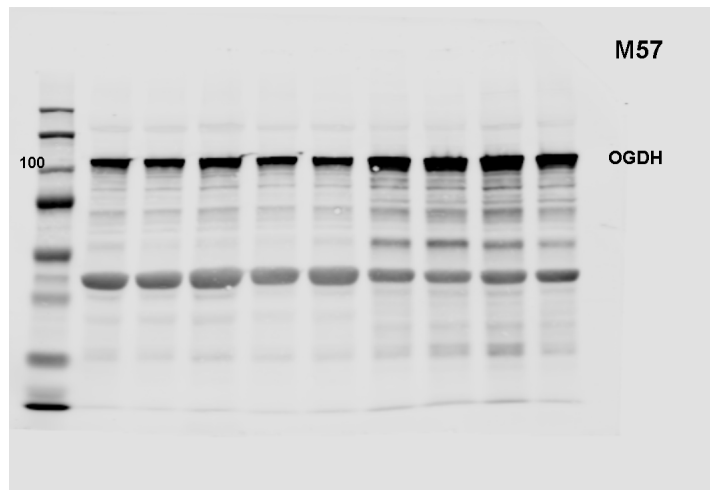

Supplement: Supplementary file 4 — Source Data for Figure 2 [file EMMM-15-e16951-s002.zip › Figure 2/2E-F/OGDH_2019-04-30.pdf]

Image Display Parameters

| Channel | Color                       | Minimum | Maximum | K |
|---------|-----------------------------|---------|---------|---|
| 700     | Gray Scale (Black on White) | 0.108   | 80.4    | 0 |

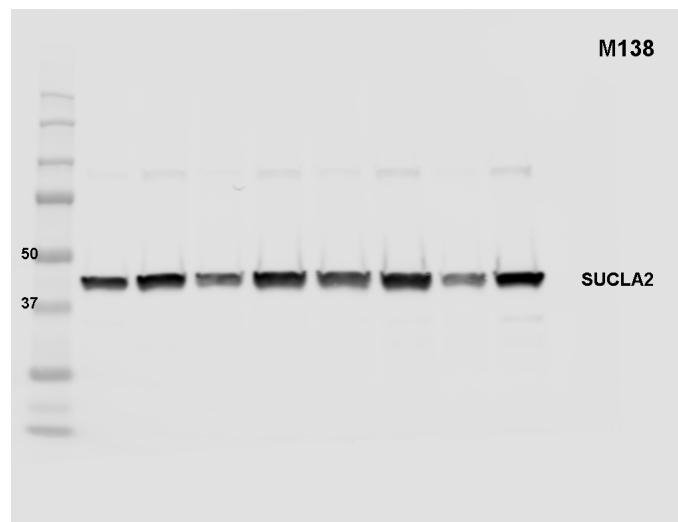

Supplement: Supplementary file 4 — Source Data for Figure 2 [file EMMM-15-e16951-s002.zip › Figure 2/2M-N/SUCLA2_2019-05-01.pdf]

Image Display Parameters

| Channel | Color                       | Minimum | Maximum | K |
|---------|-----------------------------|---------|---------|---|
| 700     | Gray Scale (Black on White) | 0.132   | 3.34    | 0 |

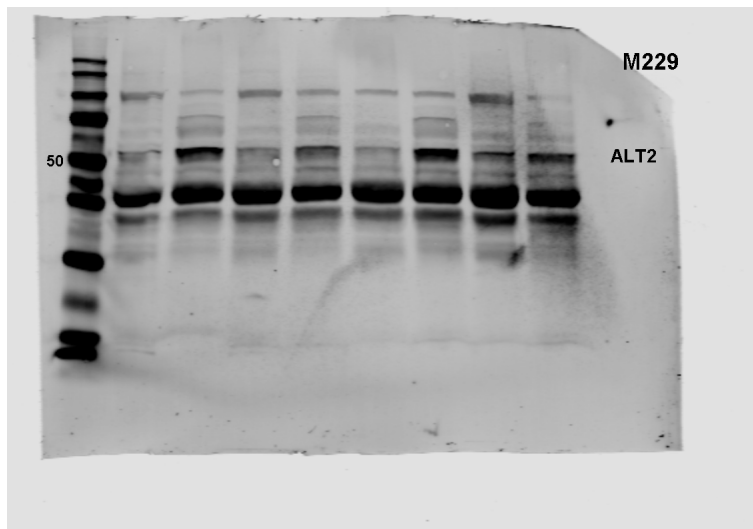

Supplement: Supplementary file 4 — Source Data for Figure 2 [file EMMM-15-e16951-s002.zip › Figure 2/2A-B/ALT2_2018-12-18.pdf]

Image Display Parameters

| Channel | Color                       | Minimum | Maximum | K |
|---------|-----------------------------|---------|---------|---|
| 800     | Gray Scale (Black on White) | 0.157   | 55.8    | 0 |

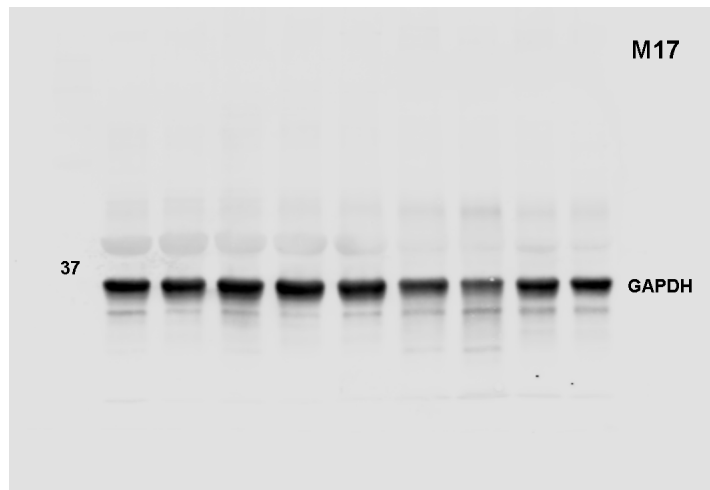

Supplement: Supplementary file 4 — Source Data for Figure 2 [file EMMM-15-e16951-s002.zip › Figure 2/2G-H/GAPDH_2019-04-30.pdf]

Image Display Parameters

| Channel | Color                       | Minimum | Maximum | K |
|---------|-----------------------------|---------|---------|---|
| 700     | Gray Scale (Black on White) | 0.0408  | 3.94    | 0 |

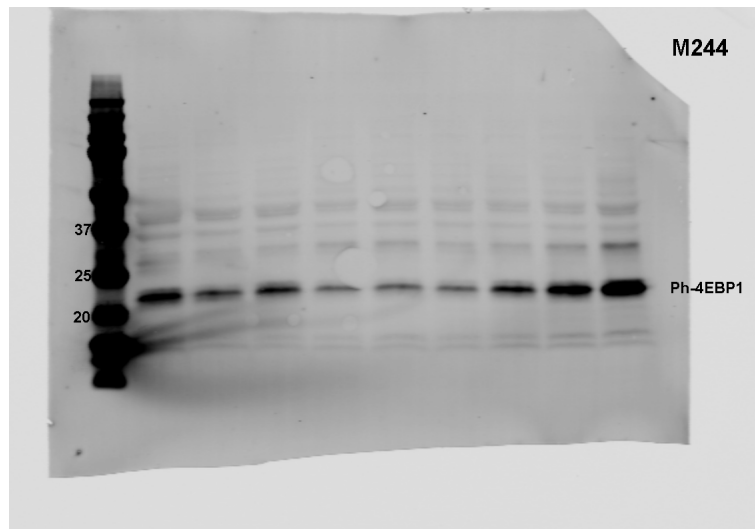

Supplement: Supplementary file 5 — Source Data for Figure 3 [file EMMM-15-e16951-s009.zip › Figure 3/3D-E/Ph-4EBP1_2019-04-23.pdf]

Image Display Parameters

| Channel | Color                       | Minimum | Maximum | K |
|---------|-----------------------------|---------|---------|---|
| 700     | Gray Scale (Black on White) | 0.101   | 5.63    | 0 |

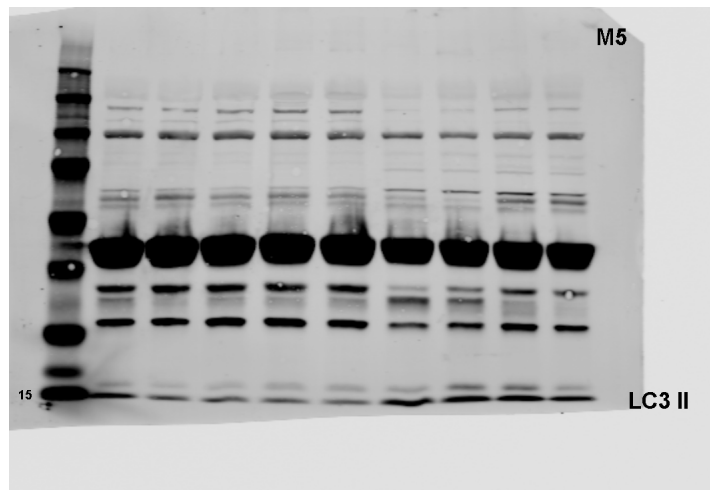

Supplement: Supplementary file 5 — Source Data for Figure 3 [file EMMM-15-e16951-s009.zip › Figure 3/3J-K/LC3 II.pdf]

Image Display Parameters

| Channel | Color                       | Minimum | Maximum | K |
|---------|-----------------------------|---------|---------|---|
| 800     | Gray Scale (Black on White) | 0.211   | 43.5    | 0 |

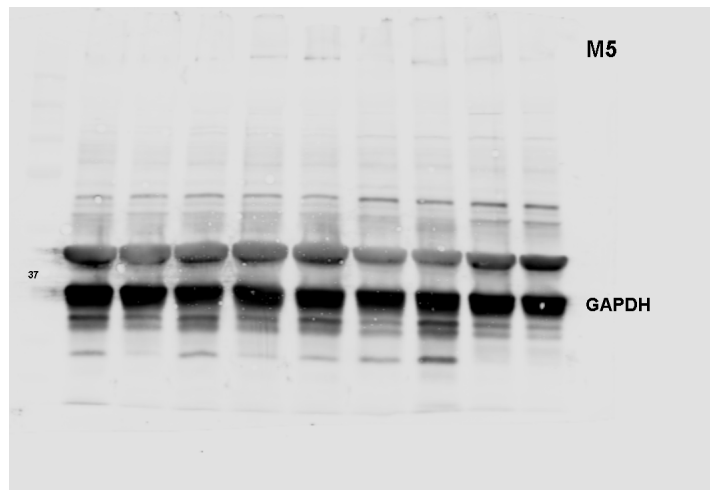

Supplement: Supplementary file 5 — Source Data for Figure 3 [file EMMM-15-e16951-s009.zip › Figure 3/3J-K/GAPDH.pdf]

Image Display Parameters

| Channel | Color                       | Minimum  | Maximum | K |
|---------|-----------------------------|----------|---------|---|
| 700     | Gray Scale (Black on White) | 0.000120 | 2.85    | 0 |

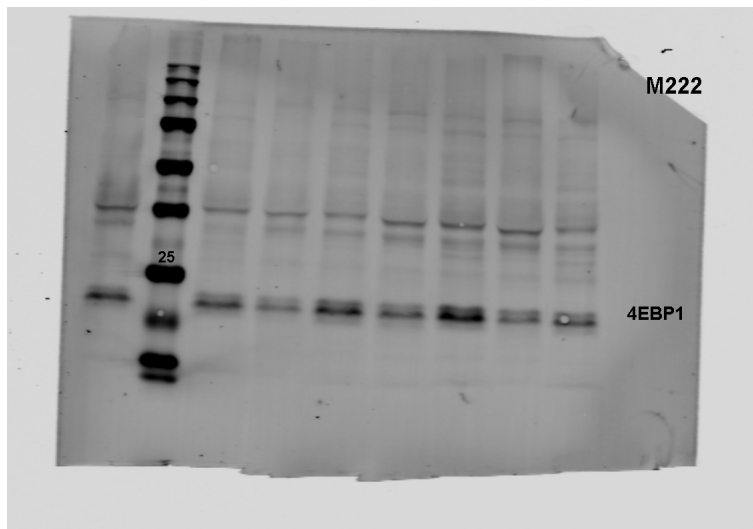

Supplement: Supplementary file 5 — Source Data for Figure 3 [file EMMM-15-e16951-s009.zip › Figure 3/3B-C/4E-BP1_2018-12-14.pdf]

Image Display Parameters

| Channel | Color                       | Minimum | Maximum | K |
|---------|-----------------------------|---------|---------|---|
| 700     | Gray Scale (Black on White) | 0.0232  | 2.73    | 0 |

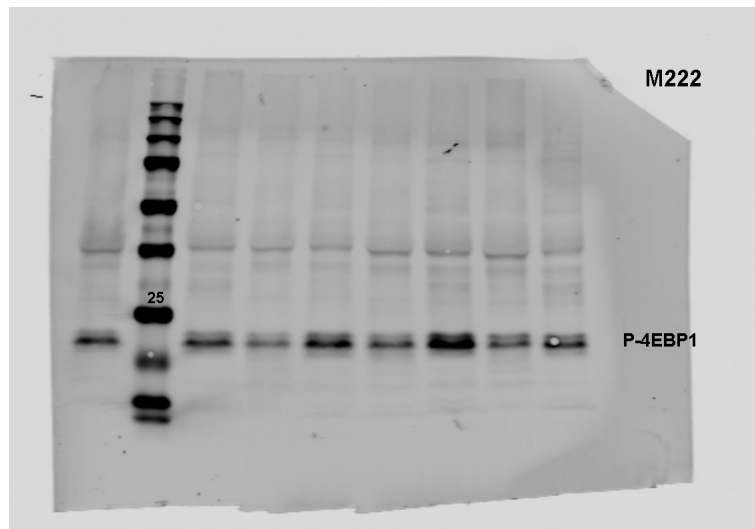

Supplement: Supplementary file 5 — Source Data for Figure 3 [file EMMM-15-e16951-s009.zip › Figure 3/3B-C/Ph-4E-BP1_2018-12-14.pdf]

Image Display Parameters

| Channel | Color                       | Minimum | Maximum | K |
|---------|-----------------------------|---------|---------|---|
| 700     | Gray Scale (Black on White) | 0.00116 | 21.5    | 0 |

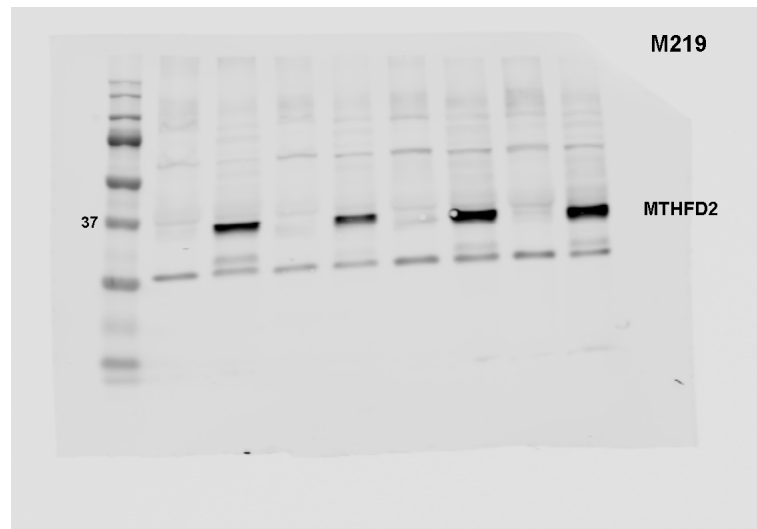

Supplement: Supplementary file 6 — Source Data for Figure 4 [file EMMM-15-e16951-s001.zip › Figure 4/4D-E/MTHFD2_2018-12-14.pdf]

Image Display Parameters

| Channel | Color                       | Minimum  | Maximum | K |
|---------|-----------------------------|----------|---------|---|
| 800     | Gray Scale (Black on White) | 0.000319 | 0.592   | 0 |

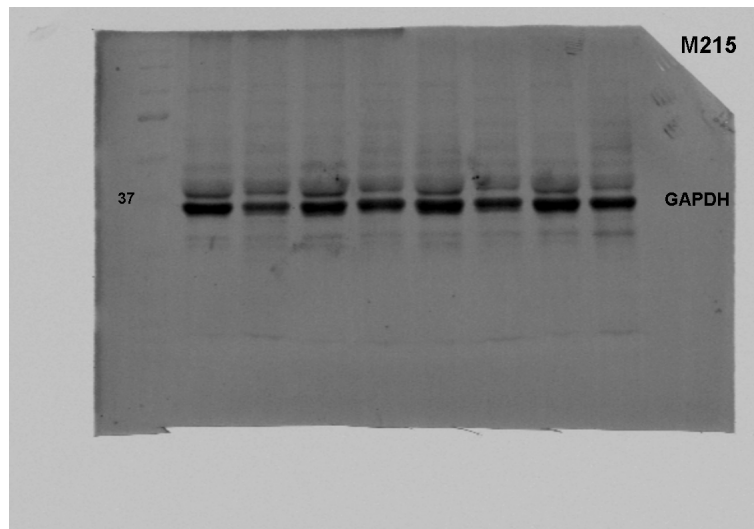

Supplement: Supplementary file 6 — Source Data for Figure 4 [file EMMM-15-e16951-s001.zip › Figure 4/4H-I/GAPDH_2018-12-14.pdf]

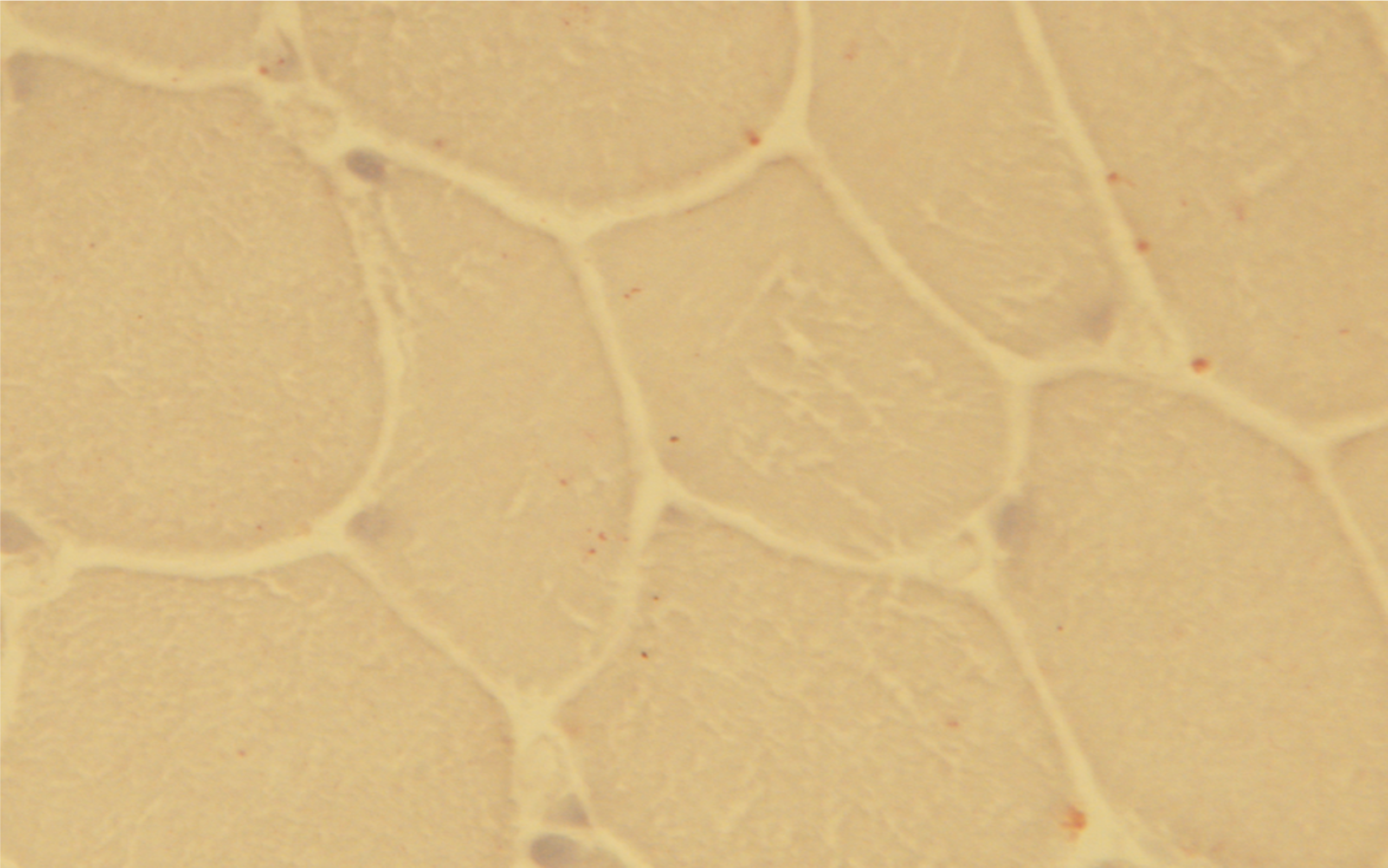

Supplement: Supplementary file 7 — Source Data for Figure 5 [file EMMM-15-e16951-s007.zip › Figure 5 4/5M/CTL.png]

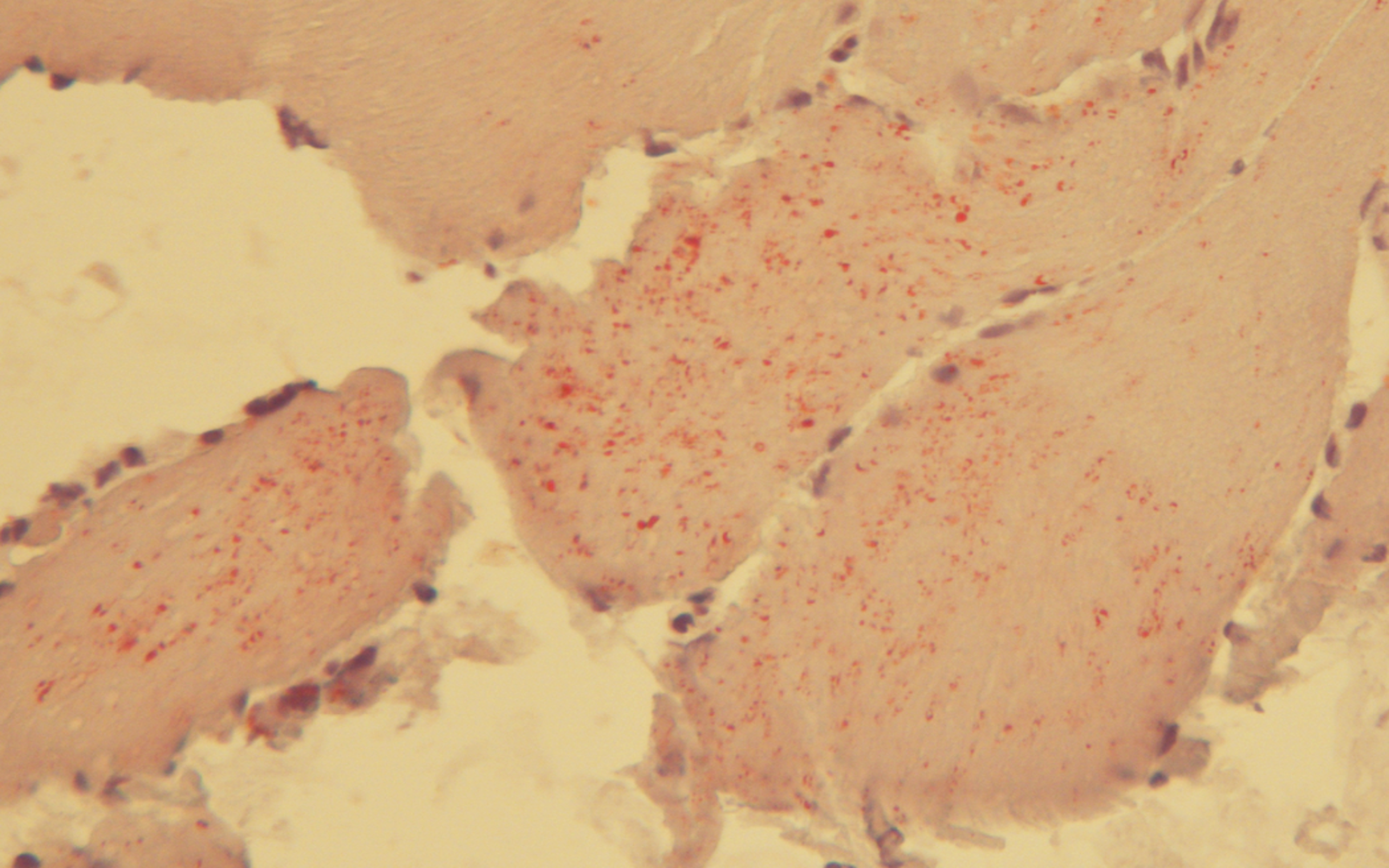

Supplement: Supplementary file 7 — Source Data for Figure 5 [file EMMM-15-e16951-s007.zip › Figure 5 4/5M/MERRF_1.png]

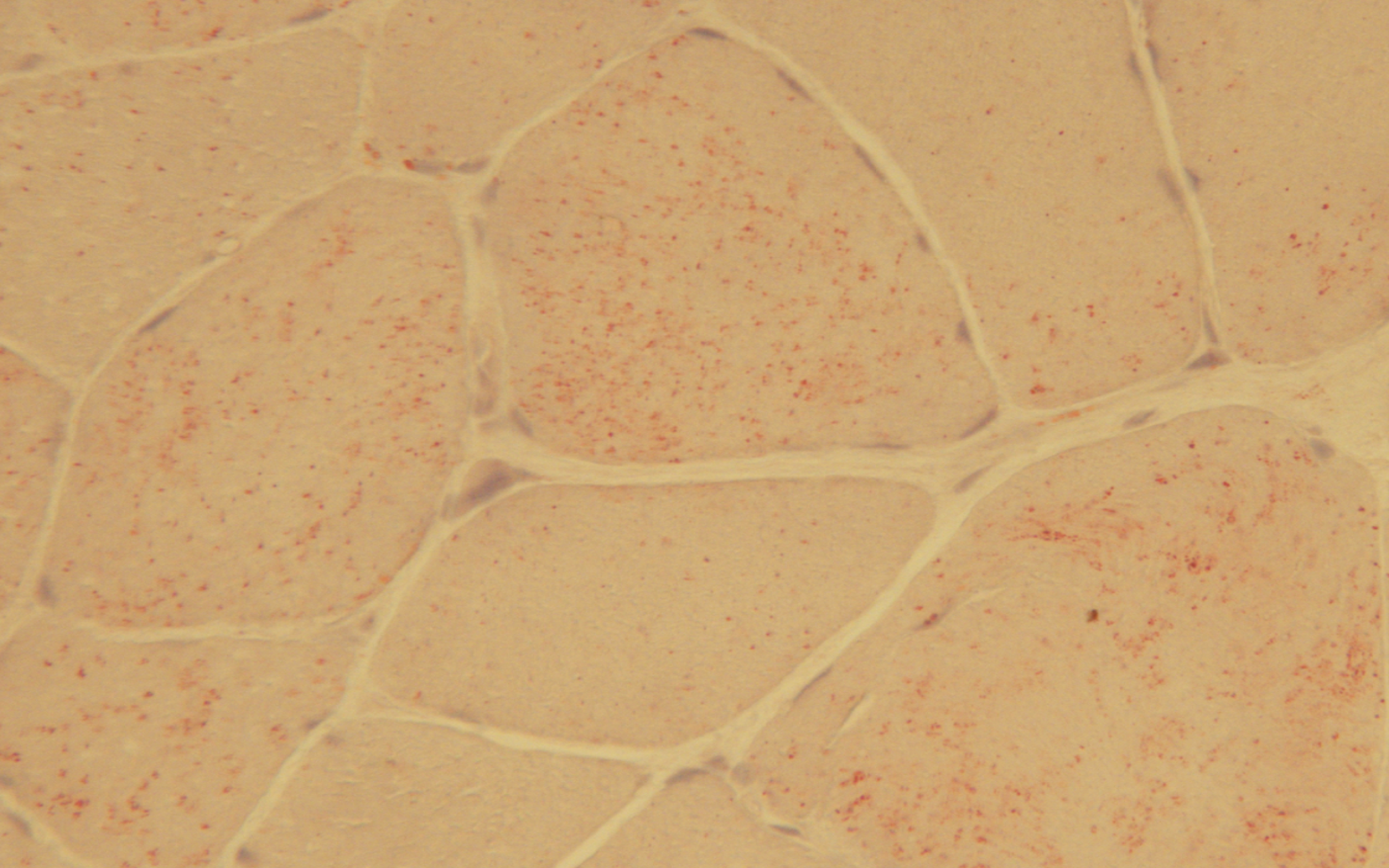

Supplement: Supplementary file 7 — Source Data for Figure 5 [file EMMM-15-e16951-s007.zip › Figure 5 4/5M/MERRF_2.png]

Image Display Parameters

| Channel | Color                       | Minimum | Maximum | K |
|---------|-----------------------------|---------|---------|---|
| 700     | Gray Scale (Black on White) | 0.00197 | 67.9    | 0 |

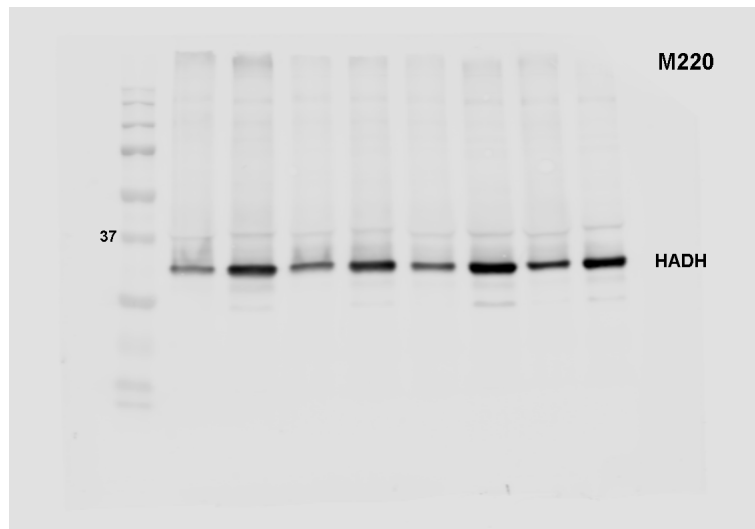

Supplement: Supplementary file 7 — Source Data for Figure 5 [file EMMM-15-e16951-s007.zip › Figure 5 4/5D-E/HADH_2018-12-14.pdf]

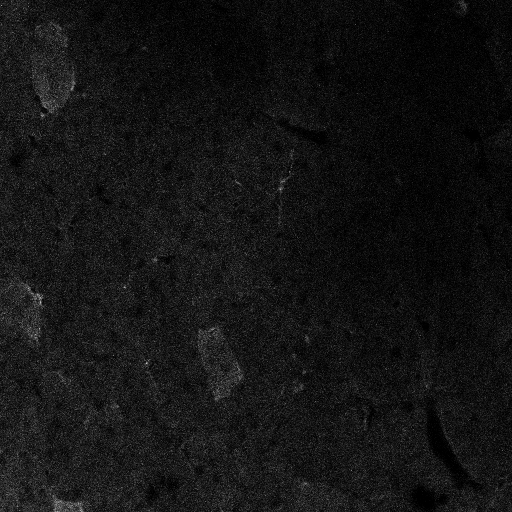

Supplement: Supplementary file 7 — Source Data for Figure 5 [file EMMM-15-e16951-s007.zip › Figure 5 4/5J/COX10 CTL 200d Muscle 20x.tif]

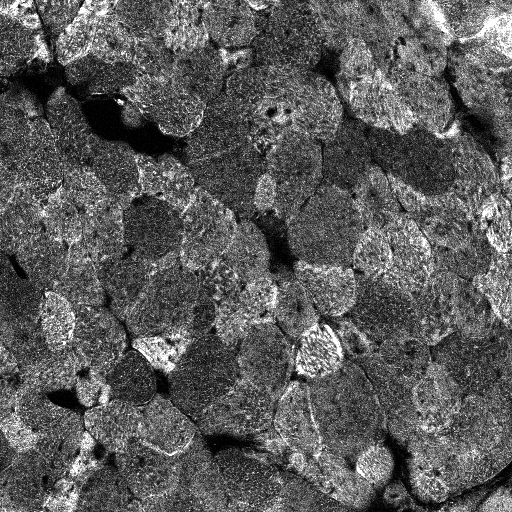

Supplement: Supplementary file 7 — Source Data for Figure 5 [file EMMM-15-e16951-s007.zip › Figure 5 4/5J/COX10 KO 200d Muscle 20X.tif]

Image Display Parameters

| Channel | Color                       | Minimum | Maximum | K |
|---------|-----------------------------|---------|---------|---|
| 700     | Gray Scale (Black on White) | 0.0892  | 2.98    | 0 |

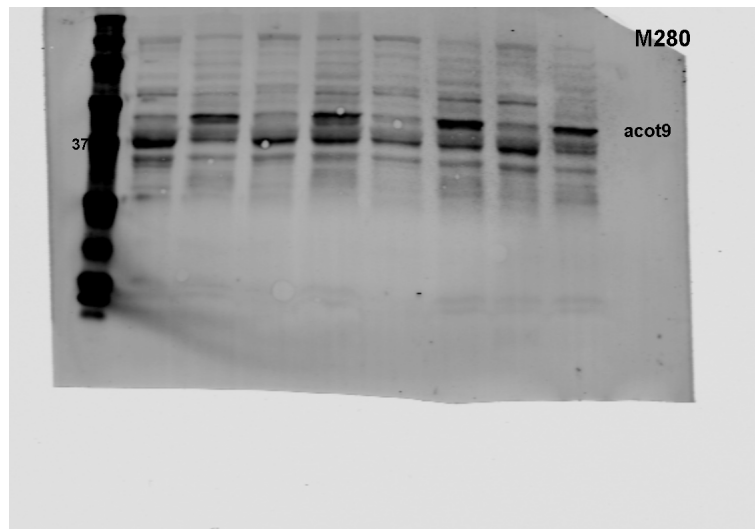

Supplement: Supplementary file 7 — Source Data for Figure 5 [file EMMM-15-e16951-s007.zip › Figure 5 4/5H-I/acot9.pdf]

Image Display Parameters

| Channel | Color                       | Minimum | Maximum | K |
|---------|-----------------------------|---------|---------|---|
| 800     | Gray Scale (Black on White) | 0.00347 | 47.6    | 0 |

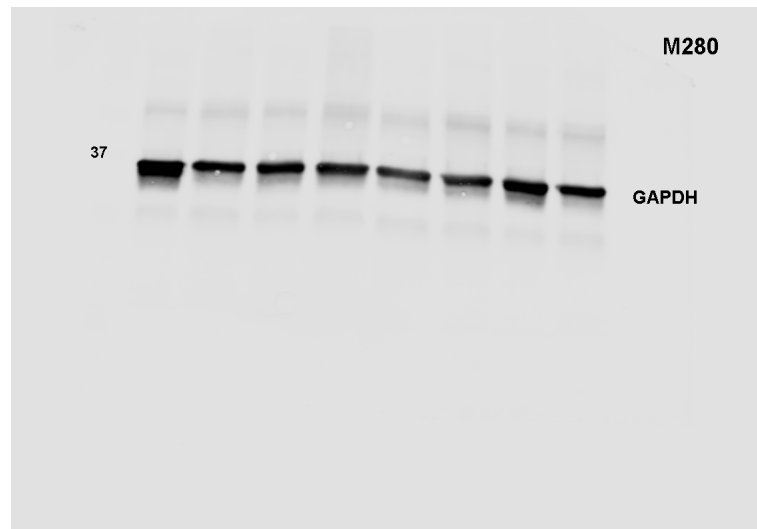

Supplement: Supplementary file 7 — Source Data for Figure 5 [file EMMM-15-e16951-s007.zip › Figure 5 4/5H-I/GAPDH.pdf]

Image Display Parameters

| Channel | Color                       | Minimum  | Maximum | K |
|---------|-----------------------------|----------|---------|---|
| 700     | Gray Scale (Black on White) | 0.000383 | 5.11    | 0 |

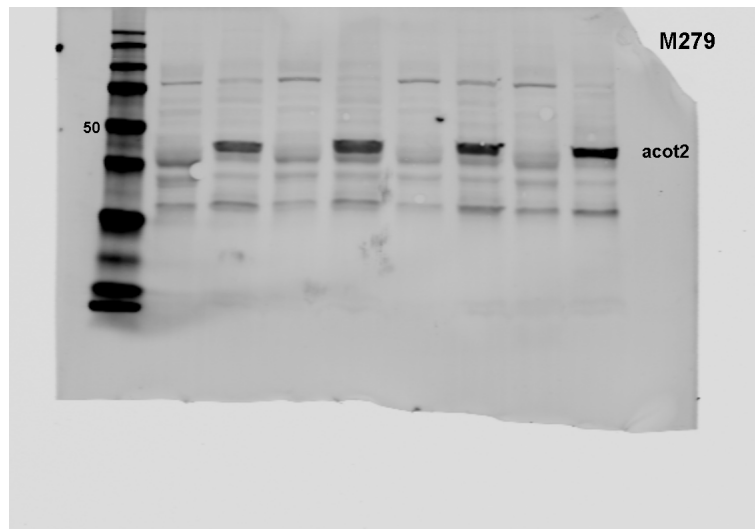

Supplement: Supplementary file 7 — Source Data for Figure 5 [file EMMM-15-e16951-s007.zip › Figure 5 4/5F-G/acot2.pdf]

Image Display Parameters

| Channel | Color                       | Minimum | Maximum | K |
|---------|-----------------------------|---------|---------|---|
| 800     | Gray Scale (Black on White) | 0.147   | 43.4    | 0 |

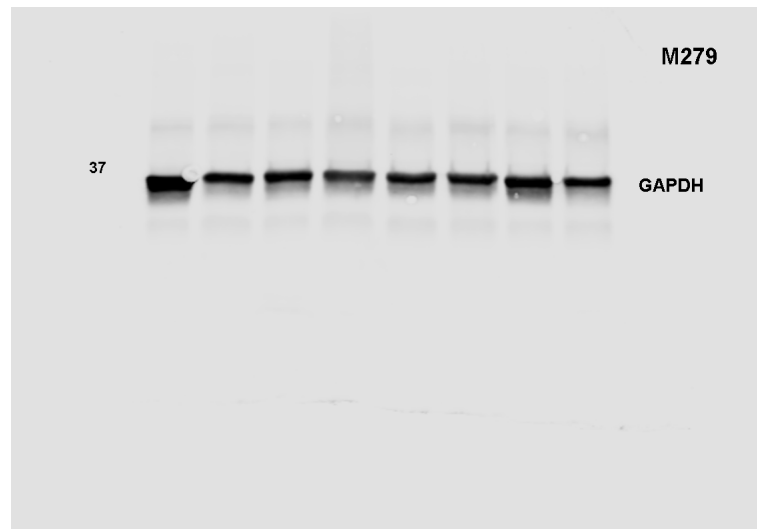

Supplement: Supplementary file 7 — Source Data for Figure 5 [file EMMM-15-e16951-s007.zip › Figure 5 4/5F-G/GAPDH.pdf]

Image Display Parameters

| Channel | Color                       | Minimum | Maximum | K |
|---------|-----------------------------|---------|---------|---|
| 700     | Gray Scale (Black on White) | 0.227   | 16.4    | 0 |

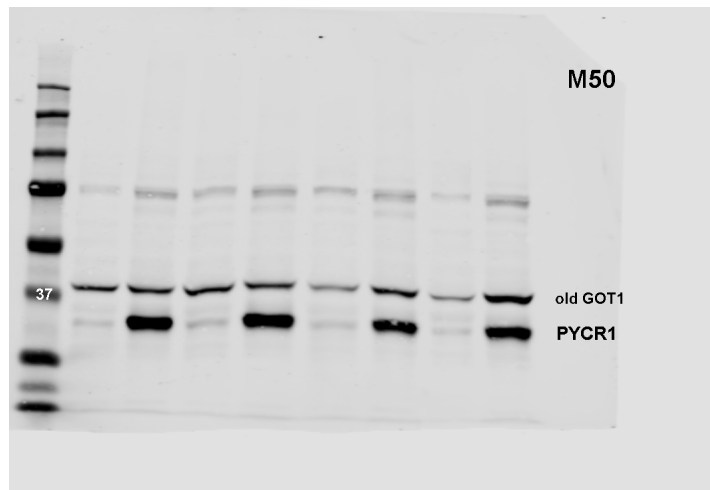

Supplement: Supplementary file 8 — Source Data for Figure 6 [file EMMM-15-e16951-s008.zip › Figure 6/Fig. 6D-E/PYCR1_2018-12-19.pdf]

Image Display Parameters

| Channel | Color                       | Minimum | Maximum | K |
|---------|-----------------------------|---------|---------|---|
| 700     | Gray Scale (Black on White) | 0.897   | 9.47    | 0 |

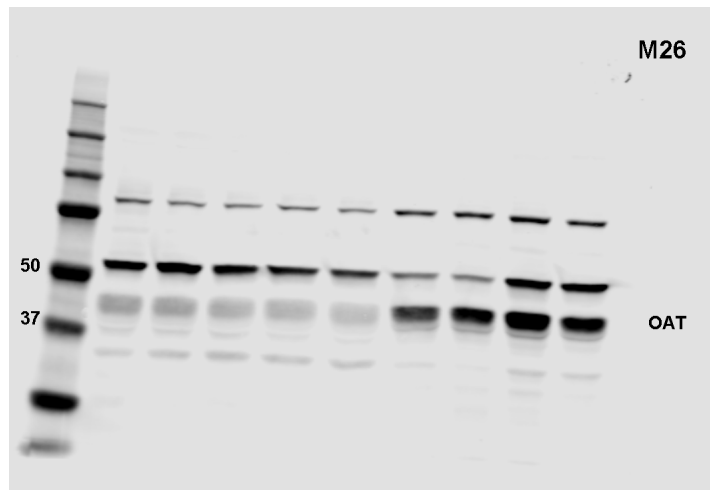

Supplement: Supplementary file 8 — Source Data for Figure 6 [file EMMM-15-e16951-s008.zip › Figure 6/Fig. 6H-I/OAT_2019-04-29.pdf]

Image Display Parameters

| Channel | Color                       | Minimum | Maximum | K |
|---------|-----------------------------|---------|---------|---|
| 700     | Gray Scale (Black on White) | 0.00616 | 3.62    | 0 |

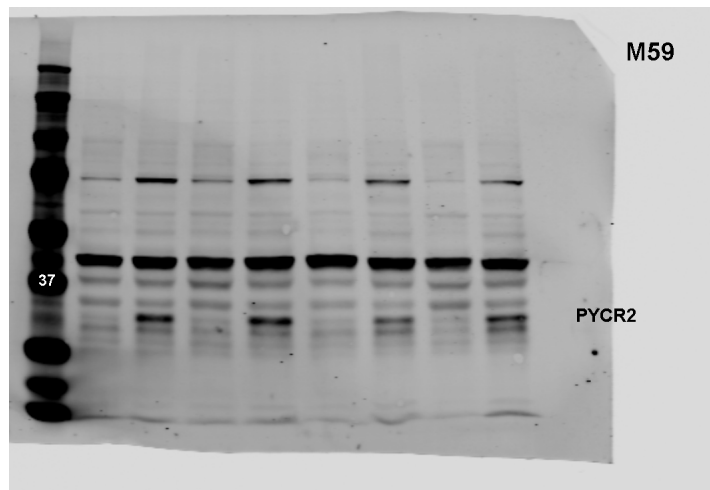

Supplement: Supplementary file 8 — Source Data for Figure 6 [file EMMM-15-e16951-s008.zip › Figure 6/Fig. 6F-G/PYCR2_2019-04-29.pdf]

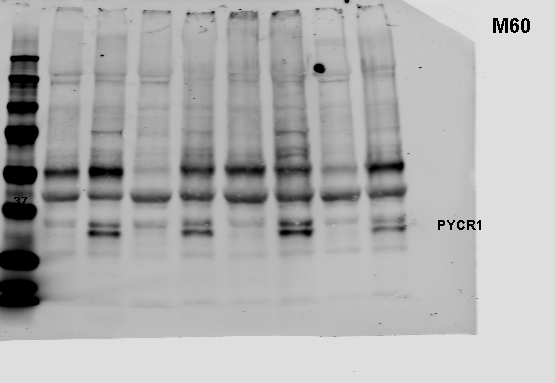

Supplement: Supplementary file 8 — Source Data for Figure 6 [file EMMM-15-e16951-s008.zip › Figure 6/Fig. 6N-O/PYCR1.pptx.tif]

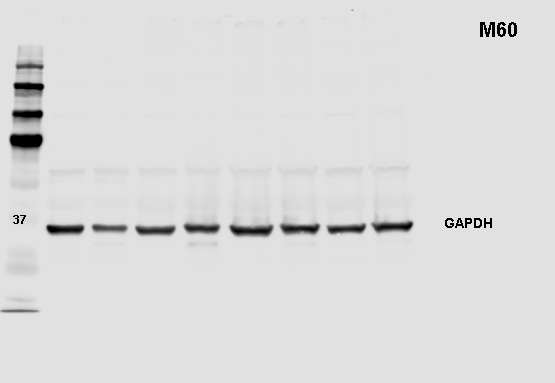

Supplement: Supplementary file 8 — Source Data for Figure 6 [file EMMM-15-e16951-s008.zip › Figure 6/Fig. 6N-O/GAPDH.pptx.tif]
