# Supplementary material for: A coordinated multiorgan metabolic response contributes to human mitochondrial myopathy
Source: EMBO Mol Med. 2023 May 24;15(7):e16951. doi: 10.15252/emmm.202216951 (PMC10331581; doi:10.15252/emmm.202216951)

Image ID 0006514_01
May 1, 2019



Image Display Parameters

| Channel | Color | Minimum | Maximum | K |
|---|---|---|---|---|
| 700 | Gray Scale (Black on White) | 0.00981 | 22.9 | 0 |

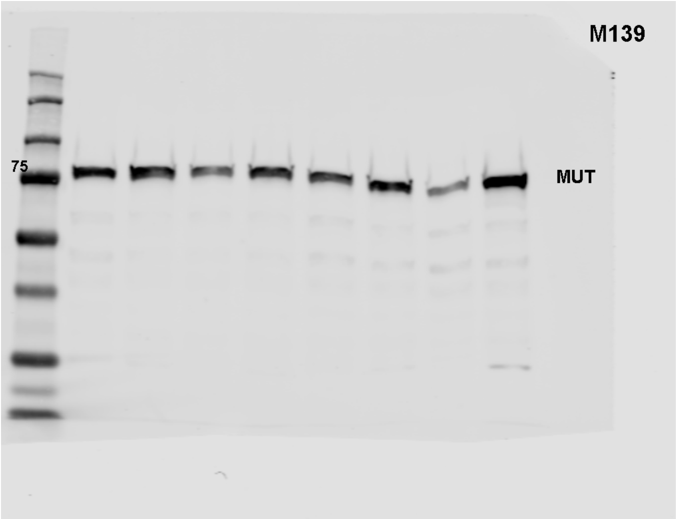

Supplement: Supplementary file 4 — Source Data for Figure 2 [file EMMM-15-e16951-s002.zip › Figure 2/2U-V/MUT_2019-05-01.pdf]
