# Supplementary material for: A coordinated multiorgan metabolic response contributes to human mitochondrial myopathy
Source: EMBO Mol Med. 2023 May 24;15(7):e16951. doi: 10.15252/emmm.202216951 (PMC10331581; doi:10.15252/emmm.202216951)
Supplement: Supplementary file 4 — Source Data for Figure 2 [file EMMM-15-e16951-s002.zip › Figure 2/2Q-R/GAPDH.pdf]

Image ID: 0008311_01
Acquire Time: Nov 16, 2021 3:50:23 PM

Acquisition Information

#	Image ID	Acquire Time	Channels	Resolution	Intensities	Quality	Analysis	Image Name
1	0008311_01	Nov 16, 2021 3:50:23 PM	700 800	169um	Auto Auto	medium	Manual	0008311_01

Image Display Values

Channel	Color	Minimum	Maximum	K
800	Gray Scale (Black on White)	0.153	10.6	0

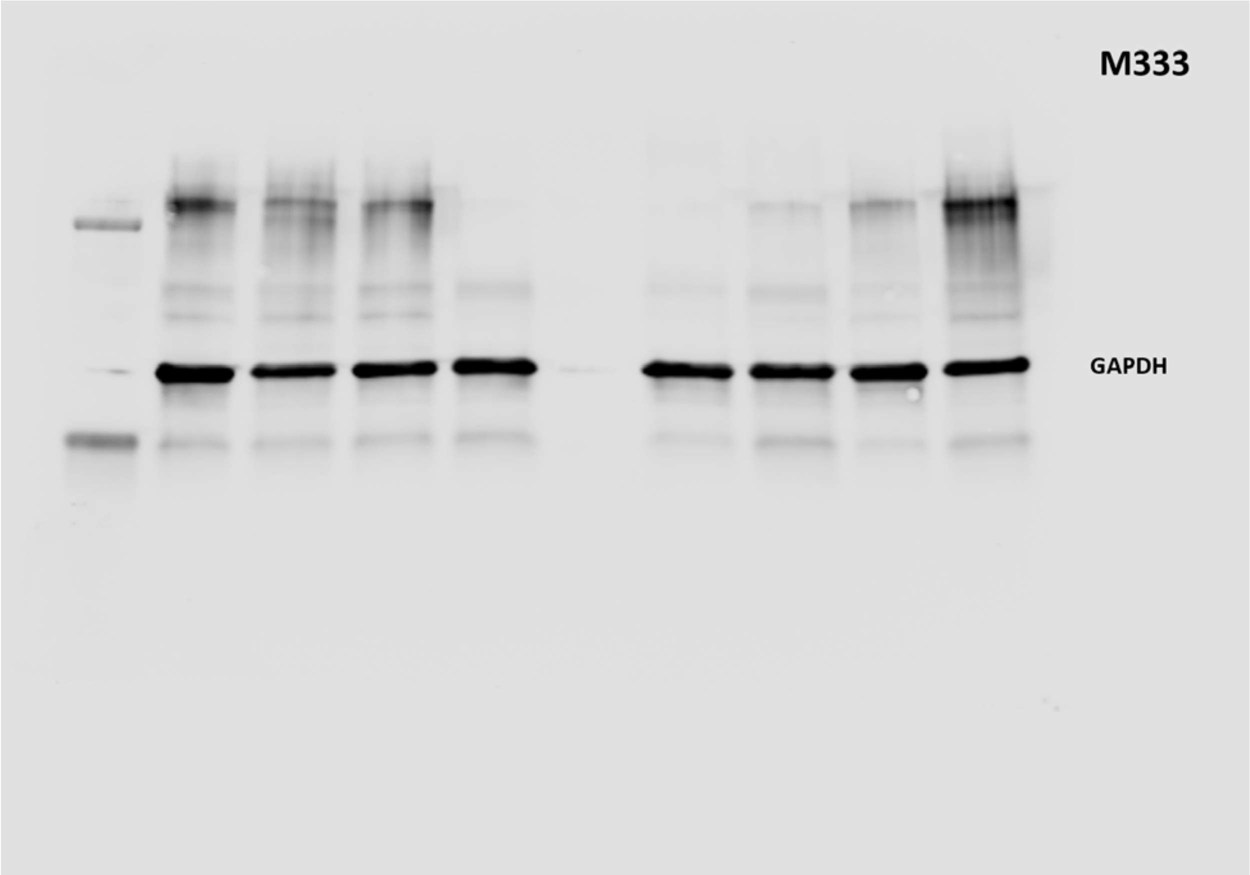

Acquisition Information (continued)

#	Comment	Image Modifications	Experiment
1	M333 COX10 KO 200days Sk mus GAPDH M		
