# Supplementary material for: A coordinated multiorgan metabolic response contributes to human mitochondrial myopathy
Source: EMBO Mol Med. 2023 May 24;15(7):e16951. doi: 10.15252/emmm.202216951 (PMC10331581; doi:10.15252/emmm.202216951)

Image ID 0006524_01
April 29, 2019

Image Display Parameters

Channel	Color	Minimum	Maximum	K
800	Gray Scale (Black on White)	0.0226	51.8	0

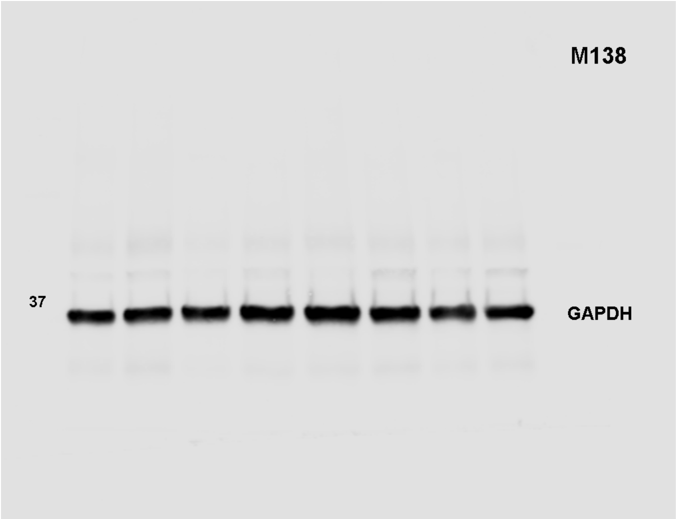

Supplement: Supplementary file 4 — Source Data for Figure 2 [file EMMM-15-e16951-s002.zip › Figure 2/2M-N/GAPDH_2019-04-29.pdf]
