# Supplementary material for: A coordinated multiorgan metabolic response contributes to human mitochondrial myopathy
Source: EMBO Mol Med. 2023 May 24;15(7):e16951. doi: 10.15252/emmm.202216951 (PMC10331581; doi:10.15252/emmm.202216951)

Image ID 0007487_01
April 23, 2019

Image Display Parameters

Channel	Color	Minimum	Maximum	K
800	Gray Scale (Black on White)	0.0611	38.1	0

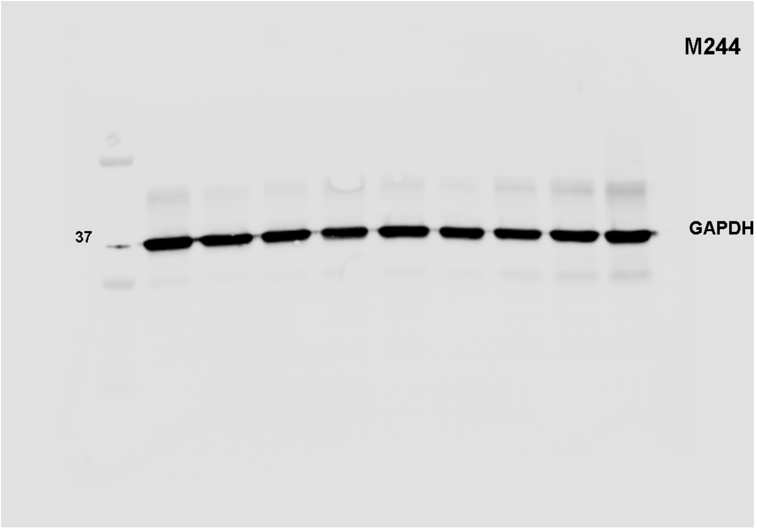

Supplement: Supplementary file 5 — Source Data for Figure 3 [file EMMM-15-e16951-s009.zip › Figure 3/3D-E/GAPDH_2019-04-23.pdf]
