# Supplementary material for: A coordinated multiorgan metabolic response contributes to human mitochondrial myopathy
Source: EMBO Mol Med. 2023 May 24;15(7):e16951. doi: 10.15252/emmm.202216951 (PMC10331581; doi:10.15252/emmm.202216951)
Supplement: Supplementary file 5 — Source Data for Figure 3 [file EMMM-15-e16951-s009.zip › Figure 3/3N-O/Atrogin.pdf]

Image ID: 0007301_01
Acquire Time: Dec 5, 2018 12:46:23 PM

Acquisition Information

#	Image ID	Acquire Time	Channels	Resolution	Intensities	Quality	Analysis	Image Name	Comment
1	0007301_01	Dec 5, 2018 12:46:23 PM	700 800	169um	Auto Auto	medium	Manual	0007301_01	M227 FBX-32 G

Image Display Values

Channel	Color	Minimum	Maximum	K
800	Gray Scale (Black on White)	0.247	22.0	0

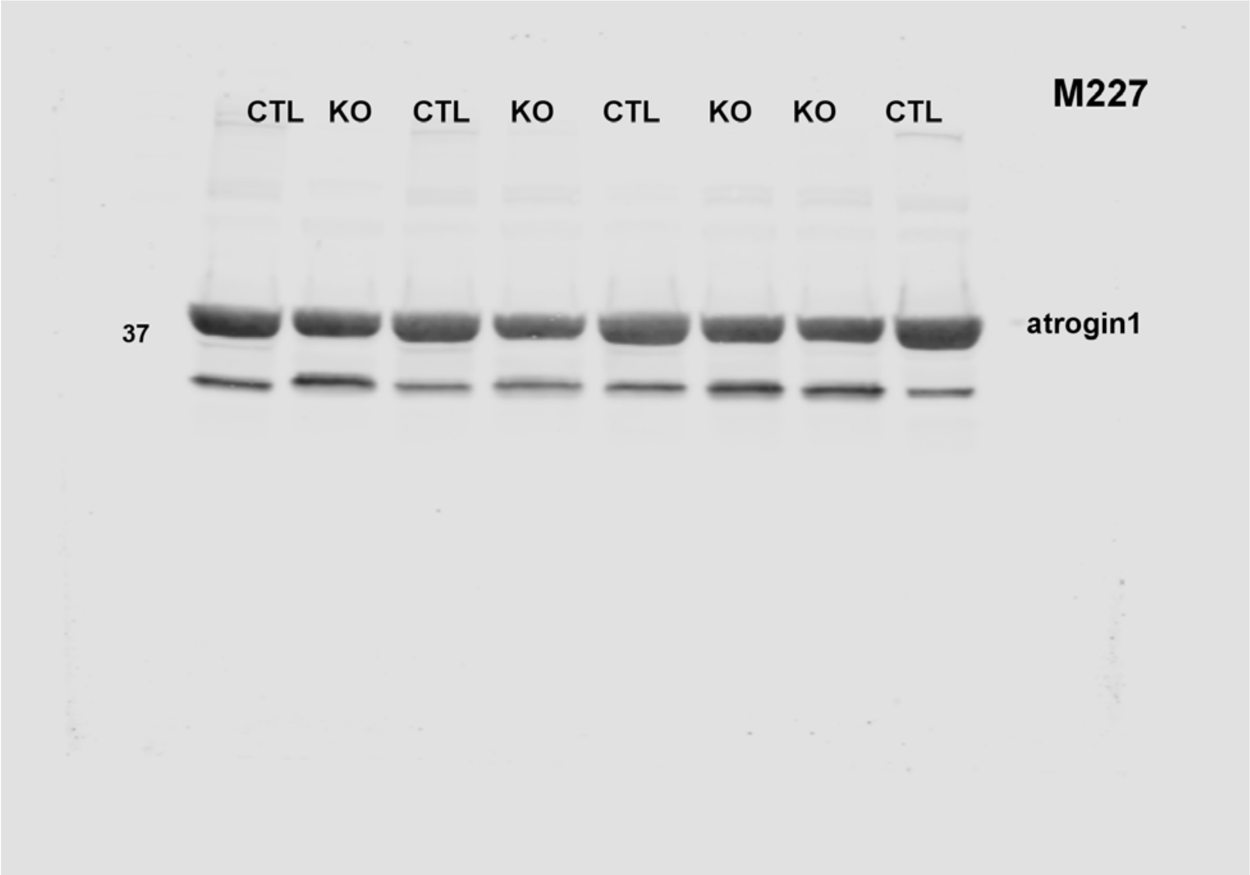

Acquisition Information (continued)

#	Image Modifications	Experiment
1		
