# Supplementary material for: A coordinated multiorgan metabolic response contributes to human mitochondrial myopathy
Source: EMBO Mol Med. 2023 May 24;15(7):e16951. doi: 10.15252/emmm.202216951 (PMC10331581; doi:10.15252/emmm.202216951)
Supplement: Supplementary file 5 — Source Data for Figure 3 [file EMMM-15-e16951-s009.zip › Figure 3/3P-Q/SESTRIN 2.pdf]

LI-COR®

Image ID: 0008983_01
Acquire Time: Mar 8, 2023 1:36:15 PM

Acquisition Information

#	Image ID	Acquire Time	Channels	Resolution	Intensities	Quality	Analysis	Image Name
1	0008983_01	Mar 8, 2023 1:36:15 PM	700 800	169um	Auto Auto	medium	Manual	0008983_01

Image Display Values

Channel	Color	Minimum	Maximum	K
700	Gray Scale (Black on White)	0.00468	5.31	0

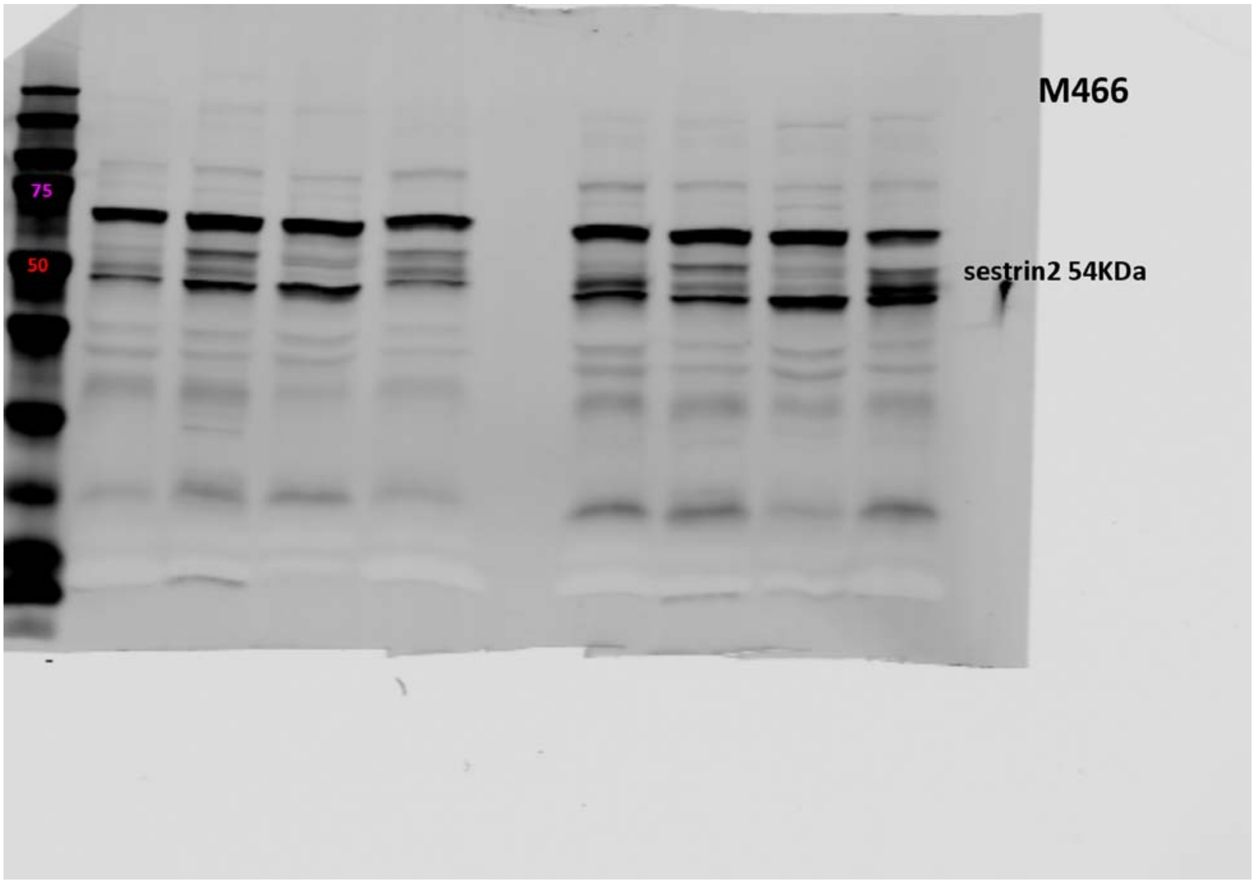

Acquisition Information (continued)

#	Comment	Image Modifications	Experiment
1	m466 Human Muscle Samples Sestrin 2 R (1:1000)		
