# Supplementary material for: A coordinated multiorgan metabolic response contributes to human mitochondrial myopathy
Source: EMBO Mol Med. 2023 May 24;15(7):e16951. doi: 10.15252/emmm.202216951 (PMC10331581; doi:10.15252/emmm.202216951)
Supplement: Supplementary file 5 — Source Data for Figure 3 [file EMMM-15-e16951-s009.zip › Figure 3/3P-Q/a-actin.pdf]

**Image ID: 0008994_01**
**Acquire Time: Mar 10, 2023 2:30:48 PM**

Acquisition Information

| # | Image ID | Acquire Time | Channels | Resolution | Intensities | Quality | Analysis | Image Name |
|---|---|---|---|---|---|---|---|---|
| 1 | 0008994_01 | Mar 10, 2023 2:30:48 PM | 700 800 | 169um | Auto Auto | medium | Manual | 0008994_01 |

Image Display Values

| Channel | Color | Minimum | Maximum | K |
|---|---|---|---|---|
| 800 | Gray Scale (Black on White) | 0.0156 | 18.0 | 0 |

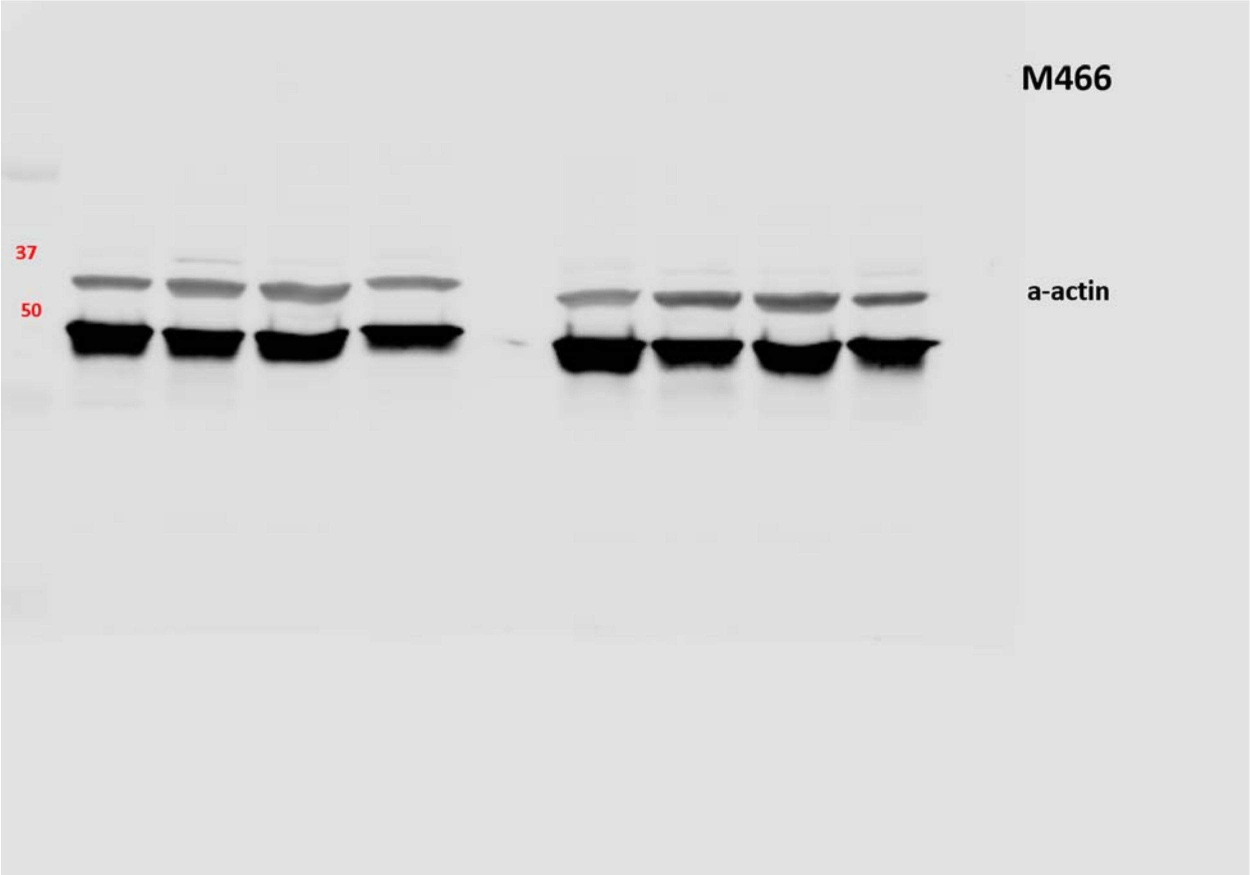

Acquisition Information (continued)

| # | Comment | Image Modifications | Experiment |
|---|---|---|---|
| 1 | M466 Human Muscle Samples A-Actin M (1:10000) | | |
