# Supplementary material for: A coordinated multiorgan metabolic response contributes to human mitochondrial myopathy
Source: EMBO Mol Med. 2023 May 24;15(7):e16951. doi: 10.15252/emmm.202216951 (PMC10331581; doi:10.15252/emmm.202216951)
Supplement: Supplementary file 6 — Source Data for Figure 4 [file EMMM-15-e16951-s001.zip › Figure 4/4F-G/gapdh.pdf]

Image ID: 0008463_01
Acquire Time: Apr 14, 2022 12:52:21 PM

Acquisition Information

#	Image ID	Acquire Time	Channels	Resolution	Intensities	Quality	Analysis	Image Name
1	0008463_01	Apr 14, 2022 12:52:21 PM	700 800	169um	Auto Auto	medium	Manual	0008463_01

Image Display Values

Channel	Color	Minimum	Maximum	K
800	Gray Scale (Black on White)	0.0781	20.7	0

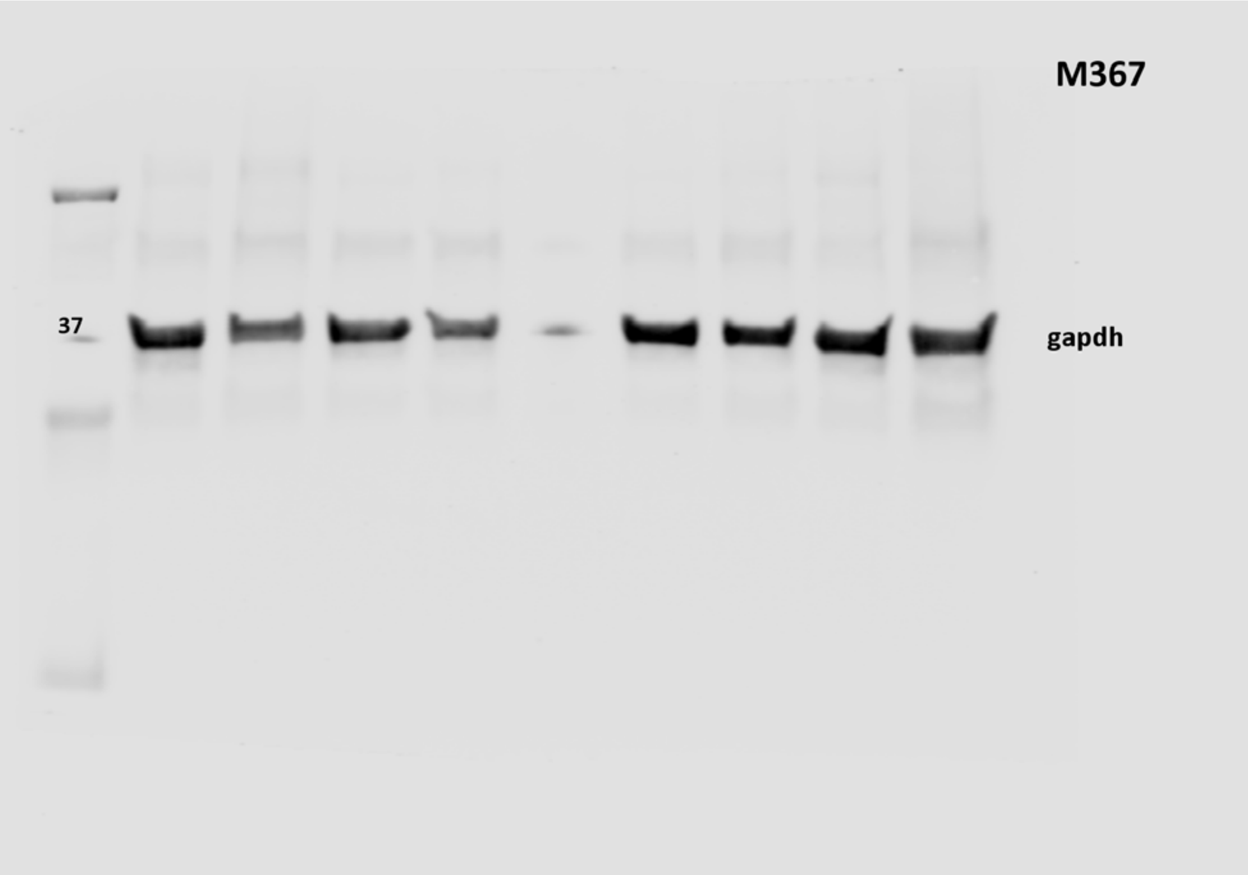

Acquisition Information (continued)

#	Comment	Image Modifications	Experiment
1	m367 COX10 200d Sk Mus GAPDH M (1:10000)		
