# Supplementary material for: A coordinated multiorgan metabolic response contributes to human mitochondrial myopathy
Source: EMBO Mol Med. 2023 May 24;15(7):e16951. doi: 10.15252/emmm.202216951 (PMC10331581; doi:10.15252/emmm.202216951)

Image ID 0007263_01
December 14, 2018

Image Display Parameters

Channel	Color	Minimum	Maximum	K
700	Gray Scale (Black on White)	3.63	10.2	0
800	Gray Scale (Black on White)	0.000800	13.2	0

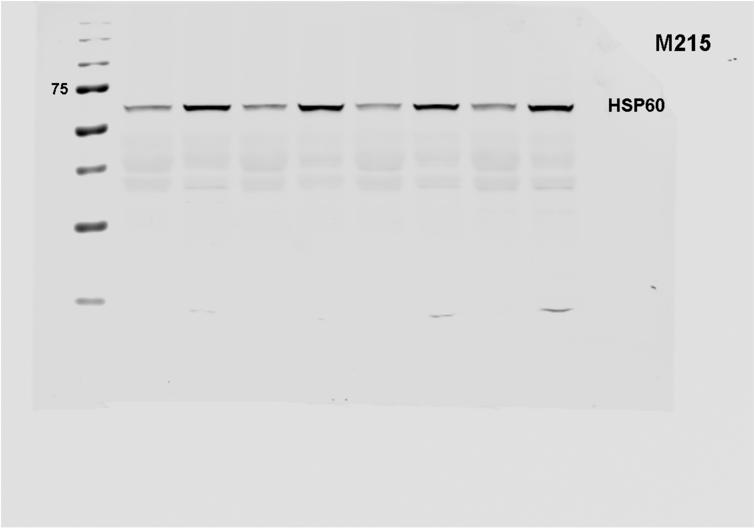

Supplement: Supplementary file 6 — Source Data for Figure 4 [file EMMM-15-e16951-s001.zip › Figure 4/4H-I/HSP60_2018-12-14.pdf]
