# Supplementary material for: A coordinated multiorgan metabolic response contributes to human mitochondrial myopathy
Source: EMBO Mol Med. 2023 May 24;15(7):e16951. doi: 10.15252/emmm.202216951 (PMC10331581; doi:10.15252/emmm.202216951)

Image ID 0005555_01
December 19, 2018

Image Display Parameters

Channel	Color	Minimum	Maximum	K
800	Gray Scale (Black on White)	0.135	55.0	0

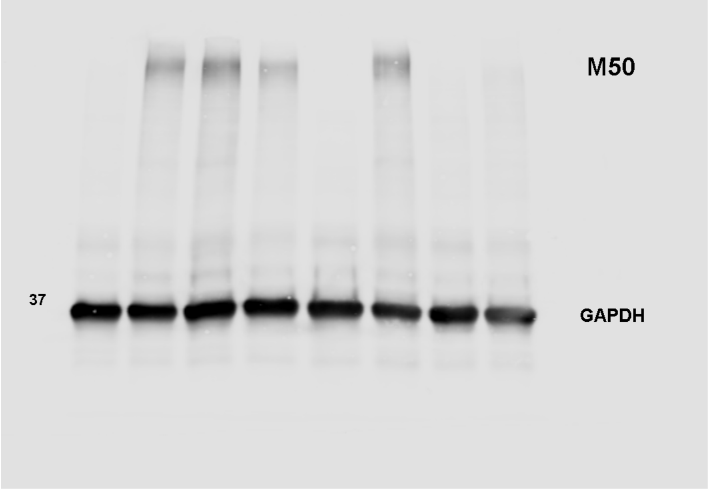

Supplement: Supplementary file 8 — Source Data for Figure 6 [file EMMM-15-e16951-s008.zip › Figure 6/Fig. 6D-E/GAPDH_2018-12-19.pdf]
