# Supplementary material for: A coordinated multiorgan metabolic response contributes to human mitochondrial myopathy
Source: EMBO Mol Med. 2023 May 24;15(7):e16951. doi: 10.15252/emmm.202216951 (PMC10331581; doi:10.15252/emmm.202216951)
Supplement: Supplementary file 8 — Source Data for Figure 6 [file EMMM-15-e16951-s008.zip › Figure 6/Fig. 6K-L/GAPDH.pdf]

LI-COR

Image ID: 0008190_01
Acquire Time: Feb 23, 2021 12:47:38 PM

Acquisition Information

#	Image ID	Acquire Time	Channels	Resolution	Intensities	Quality	Analysis	Image Name
1	0008190_01	Feb 23, 2021 12:47:38 PM	700 800	169um	Auto Auto	medium	Manual	0008190_01

Image Display Values

Channel	Color	Minimum	Maximum	K
700	Gray Scale (Black on White)	0.215	8.51	0

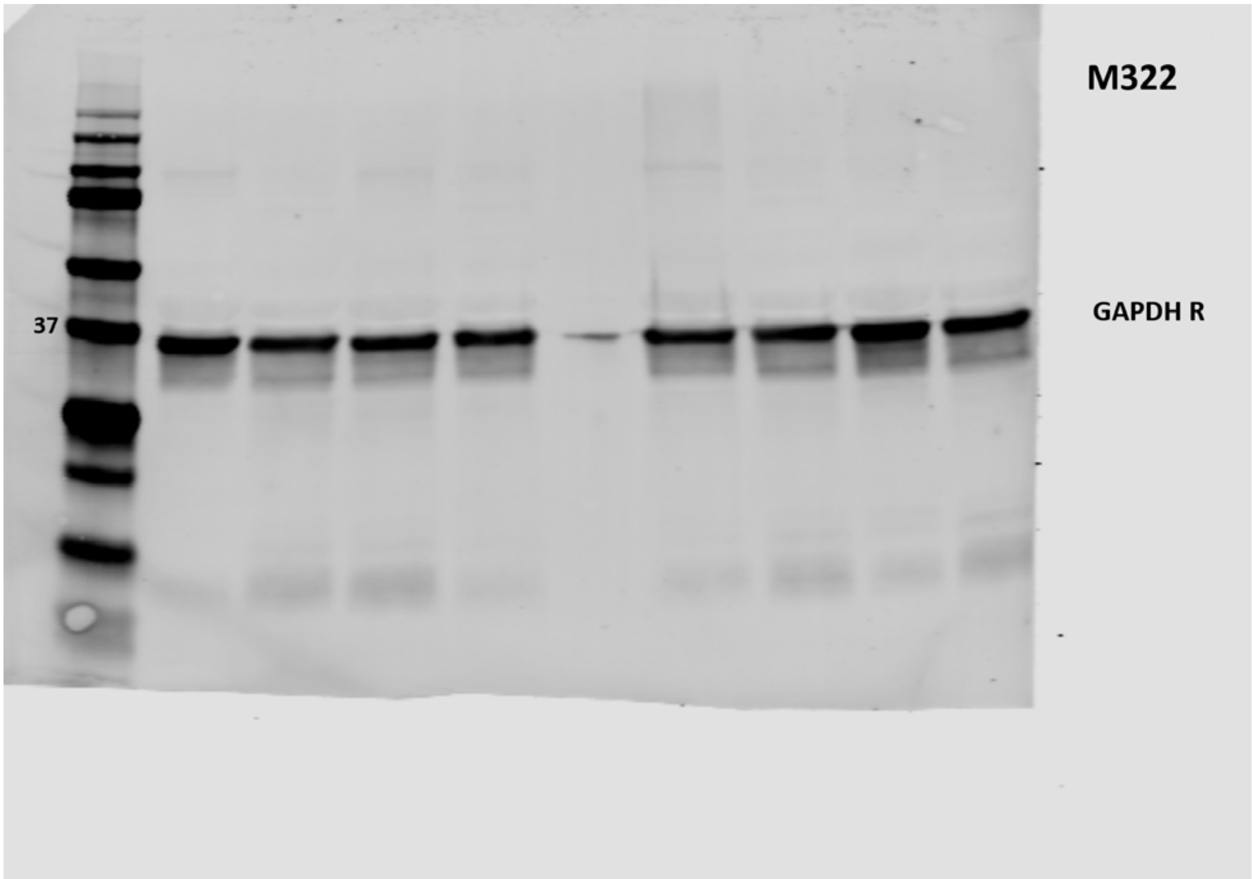

Acquisition Information (continued)

#	Comment	Image Modifications	Experiment
1	m322 COX10 200d Cohort Sk Mus Male GAPDH R (1:10000)		
