# Supplementary material for: A coordinated multiorgan metabolic response contributes to human mitochondrial myopathy
Source: EMBO Mol Med. 2023 May 24;15(7):e16951. doi: 10.15252/emmm.202216951 (PMC10331581; doi:10.15252/emmm.202216951)
Supplement: Supplementary file 10 [file EMMM-15-e16951-s010.pdf]

EMBO Molecular Medicine

A coordinated multiorgan metabolic response contributes to human mitochondrial myopathy

Nneka Southwell, Guido Primiano, Viraj Nadkarni, Nabeel Attarwala, Emelie Beattie, Dawson Miller, Sumaitaah Alam, Irene Liparulo, Yevgeniya Shurubor, Maria Lucia Valentino, Valerio Carelli, Serenella Servidei, Steven Gross, Giovanni Manfredi, Marilena D'Aurelio, and Qiuying Chen
DOI: 10.15252/emmm.202216951

Corresponding author(s): Marilena D'Aurelio (mad2003@med.cornell.edu) , Qiuying Chen (qic2005@med.cornell.edu)

Review Timeline:

Submission Date:	29th Sep 22
Editorial Decision:	2nd Nov 22
Revision Received:	13th Apr 23
Editorial Decision:	3rd May 23
Revision Received:	10th May 23
Accepted:	12th May 23

Editor: Zeljko Durdevic

Transaction Report:

(Note: With the exception of the correction of typographical or spelling errors that could be a source of ambiguity, letters and reports are not edited. Depending on transfer agreements, referee reports obtained elsewhere may or may not be included in this compilation. Referee reports are anonymous unless the Referee chooses to sign their reports.)

2nd Nov 2022

Dear Dr. D'Aurelio,

Thank you for the submission of your manuscript to EMBO Molecular Medicine. We have now received feedback from the three reviewers who agreed to evaluate your manuscript. As you will see from the reports, while the referee #3 is overall supporting the publication, referees #1 and #2 recognize potential interest of the study but also raise important concerns that should be addressed in a major revision. The experiment with aminooxy acetate suggested by the referee #1 is an informative experiment in our opinion and would strengthen the manuscript considerably, however, we understand that it will require quite some time to be performed. Taking this in consideration we think six months would be more appropriate to provide the complete revision and we are also willing to extend the revision time if necessary. Please do not hesitate to contact me if you wish to discuss this point further.

Further consideration of a revision that addresses reviewers' concerns in full will entail a second round of review. EMBO Molecular Medicine encourages a single round of revision only and therefore, acceptance or rejection of the manuscript will depend on the completeness of your responses included in the next, final version of the manuscript. For this reason, and to save you from any frustrations in the end, I would strongly advise against returning an incomplete revision.

We would welcome the submission of a revised version within six months for further consideration. Please let us know if you require longer to complete the revision.

I look forward to receiving your revised manuscript.

Yours sincerely,

Zeljko Durdevic

Zeljko Durdevic
Editor
EMBO Molecular Medicine

*****

When submitting your revised manuscript, please carefully review the instructions that follow below. We perform an initial quality control of all revised manuscripts before re-review; failure to include requested items will delay the evaluation of your revision.

We require:

1) A .docx formatted version of the manuscript text (including legends for main figures, EV figures and tables). Please make sure that the changes are highlighted to be clearly visible.

2) Individual production quality figure files as .eps, .tif, .jpg (one file per figure). For guidance, download the 'Figure Guide PDF': (https://www.embopress.org/page/journal/17574684/authorguide#figureformat).

3) A .docx formatted letter INCLUDING the reviewers' reports and your detailed point-by-point responses to their comments. As part of the EMBO Press transparent editorial process, the point-by-point response is part of the Review Process File (RPF), which will be published alongside your paper.

4) A complete author checklist, which you can download from our author guidelines (https://www.embopress.org/page/journal/17574684/authorguide#submissionofrevisions). Please insert information in the checklist that is also reflected in the manuscript. The completed author checklist will also be part of the RPF.

5) Please note that all corresponding authors are required to supply an ORCID ID for their name upon submission of a revised

manuscript.

6) It is mandatory to include a 'Data Availability' section after the Materials and Methods. Before submitting your revision, primary datasets produced in this study need to be deposited in an appropriate public database, and the accession numbers and database listed under 'Data Availability'. Please remember to provide a reviewer password if the datasets are not yet public (see https://www.embopress.org/page/journal/17574684/authorguide#dataavailability).

In case you have no data that requires deposition in a public database, please state so in this section. Note that the Data Availability Section is restricted to new primary data that are part of this study.

7) For data quantification: please specify the name of the statistical test used to generate error bars and P values, the number (n) of independent experiments (specify technical or biological replicates) underlying each data point and the test used to calculate p-values in each figure legend. The figure legends should contain a basic description of n, P and the test applied. Graphs must include a description of the bars and the error bars (s.d., s.e.m.). See also 'Figure Legend' guidelines: https://www.embopress.org/page/journal/17574684/authorguide#figureformat

8) At EMBO Press we ask authors to provide source data for the main manuscript figures. Our source data coordinator will contact you to discuss which figure panels we would need source data for and will also provide you with helpful tips on how to upload and organize the files.

9) Our journal encourages inclusion of *data citations in the reference list* to directly cite datasets that were re-used and obtained from public databases. Data citations in the article text are distinct from normal bibliographical citations and should directly link to the database records from which the data can be accessed. In the main text, data citations are formatted as follows: "Data ref: Smith et al, 2001" or "Data ref: NCBI Sequence Read Archive PRJNA342805, 2017". In the Reference list, data citations must be labeled with "[DATASET]". A data reference must provide the database name, accession number/identifiers and a resolvable link to the landing page from which the data can be accessed at the end of the reference. Further instructions are available at .

10) We replaced Supplementary Information with Expanded View (EV) Figures and Tables that are collapsible/expandable online. A maximum of 5 EV Figures can be typeset. EV Figures should be cited as 'Figure EV1, Figure EV2" etc... in the text and their respective legends should be included in the main text after the legends of regular figures.

- For the figures that you do NOT wish to display as Expanded View figures, they should be bundled together with their legends in a single PDF file called *Appendix*, which should start with a short Table of Content. Appendix figures should be referred to in the main text as: "Appendix Figure S1, Appendix Figure S2" etc.

- Additional Tables/Datasets should be labeled and referred to as Table EV1, Dataset EV1, etc. Legends have to be provided in a separate tab in case of .xls files. Alternatively, the legend can be supplied as a separate text file (README) and zipped together with the Table/Dataset file.

See detailed instructions here:

.

11) The paper explained: EMBO Molecular Medicine articles are accompanied by a summary of the articles to emphasize the major findings in the paper and their medical implications for the non-specialist reader. Please provide a draft summary of your article highlighting

- the medical issue you are addressing,

- the results obtained and

- their clinical impact.

This may be edited to ensure that readers understand the significance and context of the research. Please refer to any of our published articles for an example.

12) For more information: There is space at the end of each article to list relevant web links for further consultation by our readers. Could you identify some relevant ones and provide such information as well? Some examples are patient associations, relevant databases, OMIM/proteins/genes links, author's websites, etc...

13) Author contributions: You will be asked to provide CRediT (Contributor Role Taxonomy) terms in the submission system. These replace a narrative author contribution section in the manuscript.

14) A Conflict of Interest statement should be provided in the main text.

15) Every published paper now includes a 'Synopsis' to further enhance discoverability. Synopses are displayed on the journal webpage and are freely accessible to all readers. They include a short stand first (maximum of 300 characters, including space) as well as 2-5 one-sentences bullet points that summarizes the paper. Please write the bullet points to summarize the key NEW findings. They should be designed to be complementary to the abstract - i.e. not repeat the same text. We encourage inclusion of key acronyms and quantitative information (maximum of 30 words / bullet point). Please use the passive voice. Please attach these in a separate file or send them by email, we will incorporate them accordingly.

Please also suggest a striking image or visual abstract to illustrate your article as a PNG file 550 px wide x 300-600 px high.

EMBO Molecular Medicine has a "scooping protection" policy, whereby similar findings that are published by others during review or revision are not a criterion for rejection. Should you decide to submit a revised version, I do ask that you get in touch after three months if you have not completed it, to update us on the status.

Please note: When submitting your revision you will be prompted to enter your funding and payment information. This will allow Wiley to send you a quote for the article processing charge (APC) in case of acceptance. This quote takes into account any reduction or fee waivers that you may be eligible for. Authors do not need to pay any fees before their manuscript is accepted and transferred to the publisher.

EMBO Press participates in many Publish and Read agreements that allow authors to publish Open Access with reduced/no publication charges. Check your eligibility: https://authorservices.wiley.com/author-resources/Journal-Authors/open-access/affiliation-policies-payments/index.html

***** Reviewer's comments *****

Referee #1 (Comments on Novelty/Model System for Author):

nice combination of human and mouse
Metabolomics is well done

Referee #1 (Remarks for Author):

This is an interesting paper that demonstrates elevation of oxidation of amino acids in mitochondrial disease patients and mouse model of mitochondrial myopathy (COX10 KO). The paper is largely well executed however there are two key points that need to be addressed.

(1) It is not clear what is the causal role of amino acid catabolism via aminotransferases into TCA cycle. Please test whether this is adaptive or maladaptive by administering aminooxy acetate (AOA) to mice. AOA has been administered to mice. AOA will inhibit aminotransferases and prevent glutamate conversion to aKG. What happens to the mice grip strength or some other functional output as well as glutamate tracing in vivo with AOA.
see AOA in mice reference:PMID: 23153536

(2) The OGDH reaction is NAD+ dependent and succinate to fumarate uses SDH. COX10 KO will not allow complex I to regenerate NAD+ and SDH to function. I am struggling how oxidative TCA cycle is functioning in COX10 KO. They report a decreased NAD+/NADH ratio in COX10 KO vs. CTL muscle. Is there any reductive carboxylation going on (citrate m+5 from glutamate)? I guess proline synthesis can generate some NAD+ to keep oxidative cycle going but how does SDH functions in this setting?

Referee #2 (Comments on Novelty/Model System for Author):

The authors used elegant moltiomic approaches to investiagte the metabolic and molecular changes in skeletal muscles from MERRF patients and Cox10 knockout mice. However, several of the described changes were reported before, and the novelty of the findings is npt always clear.
The medical impact is limited at this stage as this is a fundamental study whose impact on the clinical management of the patients and on the development of new therapies is unlcear.

Referee #2 (Remarks for Author):

The work by Southwell and colleagues uses an elegant multi-omic approach to investigate the metabolic, signalling and molecular changes associated with impaired oxidative phosphorylation in skeletal muscle samples from patients with MERRF syndrome and from a muscle-specific Cox10 knockout mouse. In keeping with previously published results, the authors found a general rewiring of energy metabolism, leading to increased glutamate oxidation, activation of the mitochondrial integrated stress response, inhibition of translation via reduced activity of mTORC1, activation of autophagy and reduced fatty acids oxidation. In addition, glutamate non-anaplerotic pathways were also increased, possibly to sustain NAD+ levels. Probably, the most original finding is the identification of a novel crosstalk pathway between defective skeletal muscles and other organs, in particular liver and white adipose tissue. This phenomenon is possibly mediated by mitokynes, such as FGF21 and GDF15, and leads to increased release of fatty acids from WAT, contributing to the increased levels in muscle and blood, and upregulation of gluconeogenesis, ureagenesis and ketogenesis in the liver. Additional findings include the dysregulation of leptin signalling Cox10 KO mice, and the presence of increased levels of glucocorticoids in mouse and patients' blood, which may contribute to the increased proteostasis found in the skeletal muscle of mice and patients.

The paper is interesting, and the experiments are elegantly performed. However, I have some concerns that the authors should address.

Major comments/concerns

The authors used skeletal muscle from both MERRF patients and muscle-specific Cox10 KO mice. However, the rationale for this comparison should be made clearer. MERRF is a complex, multisystem disease with reduced activity of several respiratory chain complexes due to impaired translation of mtDNA-encoded genes, while Cox10 is a nuclear gene encoding an enzyme involved in the biosynthesis of hemeA and its disruption in mouse skeletal muscle causes severe deficiency in cytochrome c oxidase. In addition, a great amount of work was carried out to describe changes in fat metabolism in Cox10 mice, but its relevance for human disease is unclear. I also suggest the authors to better identify the potential of their findings for the clinical management of the patients and/or for the development of new therapies.
Some of the described changes are not entirely new, including those related to mtISR, 1C metabolism, trans-sulfuration, etc... I recommend the authors highlight the novelties compared to previously published data.
I am not convinced by the interpretation of some results. For instance, the authors interpret the increased levels of asparagine, glutamate, and their acetylated derivatives (N-acetyl asparagine and N-acetyl glutamate as an increase in amino acid utilization. However, in my opinion, this could be seen as the result of reduced utilization leading to accumulaiton.
In addition, I find incorrect the authors' interpretation of the data on autophagy. The authors say that "the levels of the autophagy biomarker proteins LC3II and p62 were significantly elevated only at the late stage of myopathy", but this is most like reflecting a block in autophagy, not an activation. However, this can be proven only by using colchicine as a blocker of muscle autophagic flux. As this would be a very time-consuming experiment, I am only suggesting the authors consider and eventually discuss this possibility.

Specific/minor comments

Page 9: the sentence "the variable upregulation among the markers...suggests the operation of complex regulatory processes" sounds too vague.
Page 12: the authors dissect mTORC1 signalling in Cox10 muscles. Can the authors speculate on why mTORC1 signalling is downregulated in this model? Is this the case also in the MERRF patients' muscles?

Referee #3 (Comments on Novelty/Model System for Author):

Novelty: The paper from Southwell et al. aims to study the muscle metabolic remodeling mechanisms involved in severe mitochondrial myopathy conditions and understands the downstream consequences on other tissue such as the liver, WAT. The current paper highlight that the muscle metabolic adaptations are part of inter-organ cross talk. It also identifies potential new targets for metabolic intervention.

Technical qualities and adequacy of the model: The metabolic responses to OXPHOS defect was first characterised in human muscle from m.8344A>G MERRF patients, and confirmed in murine model of mitochondrial myopathy.
The murine model where COX10 is excised in skeletal muscle specifically has been characterised in depth (eg Expression level of Cox1 and 4 at different time point via Western blot analysis, muscle activity tested via treadmill and grip test, visceral fat assessed, and body weight overtime reported)
Different mitochondrial markers tested (involved in protein. Transport or mitochondrial metabolism), metabolites involved in different metabolic pathways assessed as well.
Several Omic analysis have been used in the current paper, including transcriptome, metabolome.

Referee #3 (Remarks for Author):

The authors have convincingly show that OXPHOS-defective muscle adapts metabolically by implementing alternative pathways for rewiring of redox and metabolic homeostasis.

The figures are very well organised and full of information: including in all figures a graphical abstract of the metabolic pathway being investigated is very useful. All western blots are neat, as well as the histology.
The paper is reach in data and extremely complete. The discussion is very complete.

Minor comment:
Page 5 Typo: "reveled" instead of "Revealed"
Also the data generated via the different omics analysis should be deposited in a public database (I could not find any GEO number or any thing similar in the text).
In the methods, the authors should detail a bit more the omic analysis (eg the statistical approach used, etc)

Point by point response to reviewers' comments

First, we want to thank the reviewers for their thorough analysis of the manuscript and their insightful comments. They provided important suggestions for improving the manuscript. In the revision, we have addressed their comments and concerns as follows.

Referee #1

This is an interesting paper that demonstrates elevation of oxidation of amino acids in mitochondrial disease patients and mouse model of mitochondrial myopathy (COX10 KO). The paper is largely well executed however there are two key points that need to be addressed.

1) It is not clear what is the causal role of amino acid catabolism via aminotransferases into TCA cycle. Please test whether this is adaptive or maladaptive by administering aminooxy acetate (AOA) to mice. AOA has been administered to mice. AOA will inhibit aminotransferases and prevent glutamate conversion to aKG. What happens to the mice grip strength or some other functional output as well as glutamate tracing in vivo with AOA. see AOA in mice reference: PMID: 23153536

We thank the reviewer for this valuable suggestion. *In vivo* treatments with Aminooxy acetate (AOA), an inhibitor of PLP-dependent transaminases (e.g., GOT, ALT), have been effective in reducing cancer growth through the inhibition of the glutamine-glutamate utilization pathway (Korangath et al, 2015; Qing et al, 2012). Therefore, we tested the effect of AOA administration in COX10 KO mice. The *in vivo* AOA treatment was performed following the experimental condition reported by Qing G. et al. (Qing et al, 2012) as recommended by the reviewer. Briefly, 70 days old COX10 KO and CTL mice were intraperitoneally injected daily with 10 mg/Kg of AOA (treated) or PBS (untreated). After 7 weeks of treatment, exercise endurance (measured by treadmill) was decreased in AOA treated COX10 KO vs. untreated COX10 KO mice (new Fig. S2Ee). No effect was observed in CTL mice (treated vs. untreated, new Fig.S2Ff). AOA had no effect on COX10 KO and CTL mice body weight (new Fig. S2Gg), suggesting that COX10 KO weight gain arrest occurring at 100 days (Fig. S2J) is independent of glutamate oxidation.

In vivo tracing studies with [13C5, 15N]-glutamate performed at the end of the AOA treatment (130 days of age) showed decreased incorporation of the M+3 (13C) and M+1 (15N) forms of alanine in muscle and plasma of mice treated with AOA (new Fig. S2Hh-S2Ii) and decreased muscle and plasma alanine (incorporated and non-incorporated) in COX10 KO AOA vs COX10 KO PBS (although not reaching statistical significance in plasma, new Fig. S2Hh), which confirmed inhibition of glutamate to alanine conversion (by ALT) and decreased glutamate oxidation in muscle.

In summary, the worsening of exercise endurance on the treadmill by AOA suggests that upregulation of the energy-generating glutamate flux through the TCA cycle is an adaptive response in COX10 KO muscle.

Of note, because of the non-selective inhibition of AOA (e.g., AOA is also inhibitor of cystathionine β synthase, CBS; cystathionine γ lyase, CSE; GABA aminotransferase, GABA-T; ornithine aminotransferase, OAT) we cannot exclude that suppression of additional pathways (trans-sulfuration, GABA, arginine metabolism) could contribute to the worsening of COX10 KO exercise endurance. Further investigation would be required to dissect specific pathway contribution and fully address this point. The results, interpretation, and limitations of this new AOA study are presented in Results (pages 12) and Discussion (page 27).

2) The OGDH reaction is NAD+ dependent and succinate to fumarate uses SDH. COX10 KO will not allow complex I to regenerate NAD+ and SDH to function. I am struggling how oxidative TCA cycle is functioning in COX10 KO. They report a decreased NAD+/NADH ratio in COX10 KO vs. CTL muscle. Is there any reductive carboxylation going on (citrate m+5 from glutamate)? I guess proline synthesis can generate some NAD+ to keep oxidative cycle going but how does SDH functions in this setting?

We agree with the reviewer that the sources of NAD^+ supply for the α-ketoglutarate dehydrogenase complex (OGDHC) activity in COX10 KO muscle need to be better addressed. In fact, in the presence of defective respiratory chain electron transfer, NADH is expected to accumulate and limit OGDHC activity, essential for the provision of succinyl-CoA to succinyl-CoA ligase (SUCLA). It is common knowledge that NADH generated by the TCA cycle is oxidized by complex I, which re-supply NAD^+ to the TCA enzymes. However, it was shown that during respiratory inhibition and anoxic conditions (functionally comparable to COX deficiency), mitochondrial diaphorases can contribute up to 80% to the NAD^+ matrix pool and support OGDHC-mediated substrate-level phosphorylation (Kiss et al, 2014). Diaphorases, also known as DT-diaphoresis or NAD(P)H:quinone oxidoreductase (NQO), are flavoenzymes catalyzing the oxidation of reduced pyridine nucleotides (both NADH and NADPH) by endogenous electron acceptors. Among the several diaphorases isoforms, NQO1 has been found to localize not only in the cytosol but also in mitochondria from several tissues (Dong et al, 2013). Interestingly, transcript levels of *Nqo1* are upregulated in COX10 KO vs. CTL (now shown in the new Fig. S6J) suggesting that mitochondrial DT-diaphorases could contribute to the matrix NAD^+ pool regeneration in COX10 KO muscle. Interestingly, the re-oxidation of the reducible substrates of diaphorases is mediated by respiratory chain Complex III (Kiss et al, 2014) which would also allow SDH activity. However, the electron acceptor of reduced cytochrome c under conditions of oxygen deprivation and/or dysfunctional complex IV remains to be determined. Further identification and understanding of this electron acceptor (metabolite/protein) of the intermembrane space could provide viable therapeutic approaches for potentiating mitochondrial redox and energy state during OXPHOS impairment.

As accurately suggested by the reviewer, another potential source of matrix NAD^+ could derive from the reverse operation of isocitrate dehydrogenase (IDH2 and IDH3 isoforms). Reductive carboxylation of glutamate-derived αKG would predictably generate M+5 forms of citrate, (i.e., with the TCA cycle running in a counter-clockwise direction). This possibility was tested by tracing muscle incorporation of ^{13}C into the TCA cycle intermediates 30 minutes after IP injections of labeled glutamate. Our data show that muscle citrate displays an overall greater incorporation (combination of M+1, M+2, and M+4, new Fig. S6K) demonstrating increased oxidative flux of glutamate (i.e., with the TCA cycle running in a clockwise direction) in COX10 KO vs CTL muscle. The M+5 forms of citrate were not detected, indicating that during the tracer exposure reductive carboxylation does not play a major role in NAD^+ regeneration in COX10 KO muscle, at least in the time frame of the experiment.

Nevertheless, accumulation of glutamate and 3OH-FA/3OH-acylcarnitines, suggests that despite the utilization of alternative pathways of NAD^+ regeneration, such as DT-diaphorases and proline synthesis (Fig.6D-G), the NAD^+ matrix pool remains inadequate to support glutamate and FA oxidation in COX10 KO and MERRF muscle. New figures (Fig. S6J and Fig S6K) and all these considerations have been added to manuscript Results (page 21) and Discussion (page 31)

Referee #2

The work by Southwell and colleagues uses an elegant multi-omic approach to investigate the metabolic, signaling and molecular changes associated with impaired oxidative phosphorylation in skeletal muscle samples from patients with MERRF syndrome and from a muscle-specific Cox10 knockout mouse. In keeping with previously published results, the authors found a general rewiring of energy metabolism, leading to increased glutamate oxidation, activation of the mitochondrial integrated stress response, inhibition of translation via reduced activity of mTORC1, activation of autophagy and reduced fatty acids oxidation. In addition, glutamate non-anaplerotic pathways were also increased, possibly to sustain NAD+ levels. Probably, the most original finding is the identification of a novel crosstalk pathway between defective skeletal muscles and other organs, in particular liver and white adipose tissue. This phenomenon is possibly mediated by mitokynes, such as FGF21 and GDF15, and leads to increased release of fatty acids from WAT, contributing to the increased levels in muscle and blood, and upregulation of gluconeogenesis, ureagenesis and ketogenesis in the liver. Additional

findings include the dysregulation of leptin signaling Cox10 KO mice, and the presence of increased levels of glucocorticoids in mouse and patients' blood, which may contribute to the increased proteostasis found in the skeletal muscle of mice and patients.
The paper is interesting, and the experiments are elegantly performed. However, I have some concerns that the authors should address.

1) The authors used skeletal muscle from both MERRF patients and muscle-specific Cox10 KO mice. However, the rationale for this comparison should be made clearer. MERRF is a complex, multisystem disease with reduced activity of several respiratory chain complexes due to impaired translation of mtDNA-encoded genes, while Cox10 is a nuclear gene encoding an enzyme involved in the biosynthesis of heme A and its disruption in mouse skeletal muscle causes severe deficiency in cytochrome c oxidase.

We thank the reviewer for this comment, which give us the opportunity to justify the comparison between a specific clinical-genetic subgroup of mitochondrial diseases (MERRF) and the muscle-specific COX10 KO mouse. Our working hypothesis is that in patients with and in a mouse model of mitochondrial myopathy, a common metabolic response to OXPHOS defect in muscle and a conserved systemic signaling pathway contributes to mitochondrial myopathy. The rationale behind the MERRF/COX10 KO comparison resides in the muscle bioenergetic, phenotypic, and metabolic similarities which we highlight below.
The most common MERRF mutation is the m.8344A>G in the MT-TK gene encoding for transfer RNA^{Lysine}, which affects the translation of mtDNA-encoded proteins. Although this mutation can potentially affect complex I, III, IV, and V assembly, complex IV (cytochrome c oxidase, COX) remains the most affected complex in MERRF tissues and cells as clearly shown by abundance of COX-negative fibers by COX staining (new Fig. S1E) and impaired COX activity by biochemical testing (Antonicka et al, 1999; Catteruccia et al, 2015). Interestingly, the m.3243A>G mutation in the MT-TL1 gene encoding the mitochondrial $tRNA^{Leucine}$ is often associated with complex I dysfunction (Sharma et al, 2021) and results in mitochondrial encephalomyopathy with lactic acidosis and stroke-like episodes (MELAS) suggesting that mutations in mitochondrial tRNAs can impair some respiratory chain complexes more severely than others. This can explain why MERRF and COX10 KO muscle share severe COX deficiency despite their genetic differences.
We agree with the reviewer that MERRF syndrome is a complex disease, with possible multisystem involvement. However, unlike other canonical mitochondrial syndromes (such as chronic progressive external ophthalmoplegia, CPEO, and MELAS) the m.8344A>G MERRF phenotype is poorly represented by its acronym (myoclonus epilepsy with ragged-red fibers), because it is mostly characterized by myopathy (Catteruccia et al, 2015; Mancuso et al, 2013). Indeed, MERRF phenotype is among the mitochondrial syndromes belonging to the nosological group of Primary mitochondrial myopathies (PMM) (de Barcelos et al, 2019) a genetically defined group of disorders leading to defects of oxidative phosphorylation affecting predominantly skeletal muscle (Mancuso et al, 2017). Therefore, mitochondrial myopathy is a common feature of MERRF patients and COX10 KO mice. Lastly, similarities in amino acid and lipid profiles in muscle and plasma, clearly reflecting a common metabolic rewiring, suggest conserved metabolic responses to OXPHOS dysfunction in MERRF and COX10 KO muscle.
We have emphasized these clinical-biochemical-metabolic aspects in the Discussion (pages 26-27) and by adding a new Fig. S1E (COX staining in MERRF muscle).

2) In addition, a great amount of work was carried out to describe changes in fat metabolism in Cox10 mice, but its relevance for human disease is unclear. I also suggest the authors to better identify the potential of their findings for the clinical management of the patients and/or for the development of new therapies.

We thank the reviewer for this comment which give us the opportunity to better clarify the relevance of lipid metabolism in mitochondrial diseases. Dysregulation of lipid metabolism has been recently documented in m.3243A>G MELAS patients by the Mootha's group (Sharma et al, 2021). In that study, the authors found that acylcarnitines, β-hydroxy acylcarnitines (BOHCAs), and β-hydroxy fatty acids (BOHFAs) are new validated plasma markers of MELAS that strongly correlate with disease severity. These metabolites have also been identified in our study, in both muscle and plasma. Similar to our interpretation, they suggested that the fatty acid (FA) derivatives increase results from β-oxidation inhibition due to reduced NAD^+/NADH. In support of this hypothesis, BOHCAs were elevated in a mouse model of Ndufs4 deficiency (Leong et al, 2012) and accumulation of BOHFAs found in isolated mitochondria treated with respiratory chain inhibitors (Jin et al, 1992), further linking OXPHOS defect and lipid dysmetabolism. Therefore, FA derivatives in muscle and plasma could become valuable disease biomarkers for clinical management and testing of novel therapies for mitochondrial disorders. In addition, these findings justify therapeutic approaches targeting the NAD^+/NADH imbalance (Russell et al, 2020).

Increased FA levels in plasma (from WAT lipolysis) and impaired FA oxidation in muscle result in accumulation of lipids droplets in both COX10 and MERRF muscle. The ectopic accumulation of lipids in non-adipose tissue is known to be cytotoxic. Specifically, a mouse model of muscle lipid overload, the muscle-specific hLPL-mouse (Levak-Frank et al, 1995), shows skeletal muscle mass reduction, ultrastructural damage, impairment in regeneration, and severe myopathy (Tamilarasan et al, 2012). Similarly, human Lipid Storage Disorders (LSDs) lead to intramuscular lipid accumulation and impaired mitochondrial bioenergetics and progressive myopathy (Debashree et al, 2018). Moreover, lipid accumulation has been described in skeletal muscle of MERRF patients (Munoz-Malaga et al, 2000).

On the other hand, recent meta-analysis of clinical data from multiple cohorts of mitochondrial patients revealed that increased energy expenditure (hypermetabolism) is the most common disease feature which prevents accumulation of body fat and contributes to shortened patient's lifespan (Sturm et al, 2023). According to this study OXPHOS-induced ISR activation with increased extracellular secretion of metabokines produces a negative energy balance which reduces adiposity. Therefore, investigating the ISR-mediated starvation like/hypermetabolic response leading to COX10 KO muscle and WAT wasting, can shed some light on the systemic muscle-WAT regulation and help in the development of treatments that preserve adipose store.

We have included these considerations in the manuscript Discussion (pages 29-30)

3) Some of the described changes are not entirely new, including those related to mtISR, 1C metabolism, trans-sulfuration, etc... I recommend the authors highlight the novelties compared to previously published data.

We have highlighted the novelties as suggested from the reviewer. Briefly, we have emphasized the identification of a cross talk between defective skeletal muscle and other organs, such as liver and WAT. This inter-organ crosstalk mediated by myokines, leptin, and glucocorticoids signaling leads to increased WAT lipolysis and fatty acids (FA) release, contributing to FA accumulation in muscle and circulation (see also point 2), and upregulation of gluconeogenesis, ureagenesis and ketogenesis in the liver. In this revised manuscript, the reader can better evaluate how this study extends beyond previous work.

4) I am not convinced by the interpretation of some results. For instance, the authors interpret the increased levels of asparagine, glutamate, and their acetylated derivatives (N-acetyl asparagine and N-acetyl glutamate) as an increase in amino acid utilization. However, in my opinion, this could be seen as the result of reduced utilization leading to accumulation.

We agree with the reviewer that increased levels of glutamate and its acetylated derivative (N-acetyl glutamate) could reflect the muscle inability to fully oxidize glutamate. In fact, despite increased

glutamate flux through the TCA cycle (as shown by increased alanine, Fig.1C and upregulated transaminases and TCA enzymes, Fig.1I-H), glutamate oxidation by OGDHC (rate-limiting step) is still limited by NAD^+ matrix pool. This important point has been fully addressed in response to point 2 of reviewer 1.

Asparagine is synthetized from glutamate-derived aspartate by Asparagine synthetase (ASNS, Fig. 1A). Accumulation of asparagine/acetyl-asparagine most likely results from increased glutamate oxidation, ASNS upregulation (8.5-fold increase, target of ATF4, new Fig. S4G-H)), and reduced incorporation of asparagine in muscle proteins, due to reduced protein translation. Moreover, we speculate that the rapid conversion of aspartate to asparagine (by ATP-dependent ASNS) facilitates the glutamate to αKG oxidative influx in the TCA cycle by preventing accumulation of OAA, a potent inhibitor of succinate dehydrogenase. We have better clarified these points in manuscript Results (pages 6-7) and Discussion (page 31).

5) In addition, I find incorrect the authors' interpretation of the data on autophagy. The authors say that "the levels of the autophagy biomarker proteins LC3II and p62 were significantly elevated only at the late stage of myopathy", but this is most like reflecting a block in autophagy, not an activation. However, this can be proven only by using colchicine as a blocker of muscle autophagic flux. As this would be a very time-consuming experiment, I am only suggesting the authors consider and eventually discuss this possibility.

We agree with the reviewer that upregulation of LC3II and p62 at 200 days of age could reflect a block in autophagy which could at least in part be explained by the re-activation of mTORC1 as shown by recovery of Ph-4EBP1 levels (Fig. 3B). In addition, the autophagy machinery may become defective at later disease stages contributing to the increase of LC3II and p62 at 200. This interpretation has been added to the Discussion (pages 28-29).

<u>Specific/minor comments</u>

6) Page 9: the sentence "the variable upregulation among the markers...suggests the operation of complex regulatory processes" sounds too vague.

This sentence has been modified to "the variability in the upregulation of mitochondrial proteins (from 30% to 180%) at 200 days, suggests that some pathways are more upregulated than others (e.g., TCA cycle and mitochondrial import), precluding their use as mitochondrial markers" (page 9).

7) Page 12: the authors dissect mTORC1 signaling in Cox10 muscles. Can the authors speculate on why mTORC1 signaling is downregulated in this model? Is this the case also in the MERRF patients' muscles?

ATF4 induces upregulation of REDD1 and Sestrin2 implicated in mTORC1 inhibition (Xu et al, 2020) (Jang et al, 2021). Our data suggests that in COX10 KO muscle mTORC1 suppression at 100 days (shown by decreased Ph-4EBP1/4EBP1) is due to ATF4-mediated upregulation of REDD1 and Sestrin2 (Fig). At 200 days, intra-lysosomal amino acids generated by increased autophagy could reactivate mTORC1 (normal Ph-4EBP1/4EBP1). Therefore, we propose that a balance between conflicting ATF4 and mTORC1 signaling regulates COX10 KO muscle catabolic/anabolic state (added to Discussion page 28-29).

To investigate mTORC1 signaling in MERRF muscle we performed protein quantifications of mTORC1 targets and regulators (by WB). Phosphorylation levels of 4EBP1 (Ph-4EBP1/4EBP1 ratio, new Fig 3P-Q.) are similar in MERRF and CTL muscle, suggesting normal mTORC1 activity. However, ATF4, SESTRIN 2, protein levels are highly upregulated (3-, 7-, and fold change, respectively, new Fig S4G-H and 3P-Q). We propose that, similar to COX10 KO muscle, MERRF muscle proteostasis is under the regulation of a conflicting ATF4/ mTORC1 signaling. New MERRF results (Fig. 3P-Q, presented in page

14 and Fig. S4G-H, presented in page 16) have been added to the manuscript and discussed (pages 28-29).

Referee #3

The authors have convincingly show that OXPHOS-defective muscle adapts metabolically by implementing alternative pathways for rewiring of redox and metabolic homeostasis. The figures are very well organized and full of information: including in all figures a graphical abstract of the metabolic pathway being investigated is very useful. All western blots are neat, as well as the histology. The paper is reach in data and extremely complete. The discussion is very complete.

We would like to thank the reviewer for the positive feedback on the manuscript and for appreciating the quality of the experiments as well as the extent of the work performed. In this revision, we have addressed all referee 3 minor comments as follows.

Minor comment:

1) Page 5 Typo: "reveled" instead of "Revealed"

We thank the reviewer for catching this typo: "reveled" was replaced by "revealed"

2) Also the data generated via the different omics analysis should be deposited in a public database (I could not find any GEO number or anything similar in the text).

We have provided the GEO numbers for transcriptomics data in Data Availability (page 44).

3) In the methods, the authors should detail a bit more the omic analysis (eg the statistical approach used, etc)

Metabolomics data processing, as described in metabolite profiling section of the methods, was performed using commercially available Agilent metabolomics software. We have added more details for downstream processing to the statistical analyses section (Page 43). We also add statistical details to each figure legend.

References

Antonicka H, Floryk D, Klement P, Stratilova L, Hermanska J, Houstkova H, Kalous M, Drahota Z, Zeman J, Houstek J (1999) Defective kinetics of cytochrome c oxidase and alteration of mitochondrial membrane potential in fibroblasts and cytoplasmic hybrid cells with the mutation for myoclonus epilepsy with ragged-red fibres ('MERRF') at position 8344 nt. Biochem J 342 Pt 3**:** 537-544

Catteruccia M, Sauchelli D, Della Marca G, Primiano G, Cuccagna C, Bernardo D, Leo M, Camporeale A, Sanna T, Cianfoni A et al (2015) "Myo-cardiomyopathy" is commonly associated with the A8344G "MERRF" mutation. J Neurol 262**:** 701-710

de Barcelos IP, Emmanuele V, Hirano M (2019) Advances in primary mitochondrial myopathies. Curr Opin Neurol 32**:** 715-721

Debashree B, Kumar M, Keshava Prasad TS, Natarajan A, Christopher R, Nalini A, Bindu PS, Gayathri N, Srinivas Bharath MM (2018) Mitochondrial dysfunction in human skeletal muscle biopsies of lipid storage disorder. J Neurochem 145**:** 323-341

Dong H, Shertzer HG, Genter MB, Gonzalez FJ, Vasiliou V, Jefcoate C, Nebert DW (2013) Mitochondrial targeting of mouse NQO1 and CYP1B1 proteins. Biochem Biophys Res Commun 435: 727-732

Jang SK, Hong SE, Lee DH, Kim JY, Kim JY, Ye SK, Hong J, Park IC, Jin HO (2021) Inhibition of mTORC1 through ATF4-induced REDD1 and Sestrin2 expression by Metformin. BMC Cancer 21: 803

Jin SJ, Hoppel CL, Tserng KY (1992) Incomplete fatty acid oxidation. The production and epimerization of 3-hydroxy fatty acids. J Biol Chem 267: 119-125

Kiss G, Konrad C, Pour-Ghaz I, Mansour JJ, Nemeth B, Starkov AA, Adam-Vizi V, Chinopoulos C (2014) Mitochondrial diaphorases as NAD(+) donors to segments of the citric acid cycle that support substrate-level phosphorylation yielding ATP during respiratory inhibition. FASEB J 28: 1682-1697

Korangath P, Teo WW, Sadik H, Han L, Mori N, Huijts CM, Wildes F, Bharti S, Zhang Z, Santa-Maria CA et al (2015) Targeting Glutamine Metabolism in Breast Cancer with Aminooxyacetate. Clin Cancer Res 21: 3263-3273

Leong DW, Komen JC, Hewitt CA, Arnaud E, McKenzie M, Phipson B, Bahlo M, Laskowski A, Kinkel SA, Davey GM et al (2012) Proteomic and metabolomic analyses of mitochondrial complex I-deficient mouse model generated by spontaneous B2 short interspersed nuclear element (SINE) insertion into NADH dehydrogenase (ubiquinone) Fe-S protein 4 (Ndufs4) gene. J Biol Chem 287: 20652-20663

Levak-Frank S, Radner H, Walsh A, Stollberger R, Knipping G, Hoefler G, Sattler W, Weinstock PH, Breslow JL, Zechner R (1995) Muscle-specific overexpression of lipoprotein lipase causes a severe myopathy characterized by proliferation of mitochondria and peroxisomes in transgenic mice. J Clin Invest 96: 976-986

Mancuso M, McFarland R, Klopstock T, Hirano M, consortium on Trial Readiness in Mitochondrial M (2017) International Workshop:: Outcome measures and clinical trial readiness in primary mitochondrial myopathies in children and adults. Consensus recommendations. 16-18 November 2016, Rome, Italy. Neuromuscul Disord 27: 1126-1137

Mancuso M, Orsucci D, Angelini C, Bertini E, Carelli V, Comi GP, Minetti C, Moggio M, Mongini T, Servidei S et al (2013) Phenotypic heterogeneity of the 8344A>G mtDNA "MERRF" mutation. Neurology 80: 2049-2054

Munoz-Malaga A, Bautista J, Salazar JA, Aguilera I, Garcia R, Chinchon I, Segura MD, Campos Y, Arenas J (2000) Lipomatosis, proximal myopathy, and the mitochondrial 8344 mutation. A lipid storage myopathy? Muscle Nerve 23: 538-542

Qing G, Li B, Vu A, Skuli N, Walton ZE, Liu X, Mayes PA, Wise DR, Thompson CB, Maris JM et al (2012) ATF4 regulates MYC-mediated neuroblastoma cell death upon glutamine deprivation. Cancer Cell 22: 631-644

Russell OM, Gorman GS, Lightowlers RN, Turnbull DM (2020) Mitochondrial Diseases: Hope for the Future. Cell 181: 168-188

Sharma R, Reinstadler B, Engelstad K, Skinner OS, Stackowitz E, Haller RG, Clish CB, Pierce K, Walker MA, Fryer R et al (2021) Circulating markers of NADH-reductive stress correlate with mitochondrial disease severity. J Clin Invest 131

Sturm G, Karan KR, Monzel AS, Santhanam B, Taivassalo T, Bris C, Ware SA, Cross M, Towheed A, Higgins-Chen A et al (2023) OxPhos defects cause hypermetabolism and reduce lifespan in cells and in patients with mitochondrial diseases. Commun Biol 6**:** 22

Tamilarasan KP, Temmel H, Das SK, Al Zoughbi W, Schauer S, Vesely PW, Hoefler G (2012) Skeletal muscle damage and impaired regeneration due to LPL-mediated lipotoxicity. Cell Death Dis 3**:** e354

Xu D, Dai W, Kutzler L, Lacko HA, Jefferson LS, Dennis MD, Kimball SR (2020) ATF4-Mediated Upregulation of REDD1 and Sestrin2 Suppresses mTORC1 Activity during Prolonged Leucine Deprivation. J Nutr 150**:** 1022-1030

3rd May 2023

Dear Dr. D'Aurelio,

Thank you for the submission of your revised manuscript to EMBO Molecular Medicine. I am pleased to inform you that we will be able to accept your manuscript pending the following final amendments:

1) In the main manuscript file, please do the following:
- In M&M, please include statement that the informed consent was obtained from all human subjects and that the experiments conformed to the principles set out in the WMA Declaration of Helsinki and the Department of Health and Human Services Belmont Report.
- Data availability: Please be aware that all deposited datasets should be made freely available upon acceptance, without restriction. Use the following format to report the accession number of your data:

The datasets produced in this study are available in the following databases:
[data type]: [full name of the resource] [accession number/identifier] ([doi or URL or identifiers.org/DATABASE:ACCESSION])

Please check "Author Guidelines" for more information.
https://www.embopress.org/page/journal/17574684/authorguide#availabilityofpublishedmaterial
2) Author Checklist: Please check the list carefully and enter all required information. Currently information about human participants are missing.
3) Funding: Please place funding information under "Acknowledgment".
4) Synopsis:
- Synopsis text: Please provide a short standfirst (maximum of 300 characters, including space) as well as 2-5 one sentence bullet points that summarise the paper as a .doc file. Please write the bullet points to summarise the key NEW findings. They should be designed to be complementary to the abstract - i.e. not repeat the same text. We encourage inclusion of key acronyms and quantitative information (maximum of 30 words / bullet point). Please use the passive voice.
- Please check your synopsis text and image before submission with your revised manuscript. Please be aware that in the proof stage minor corrections only are allowed (e.g., typos).
5) For more information: Remove information about Appendix. This space should be used to list relevant web links for further consultation by our readers. Could you identify some relevant ones and provide such information as well? Some examples are patient associations, relevant databases, OMIM/proteins/genes links, author's websites, etc...
6) As part of the EMBO Publications transparent editorial process initiative (see our Editorial at http://embomolmed.embopress.org/content/2/9/329), EMBO Molecular Medicine will publish online a Review Process File (RPF) to accompany accepted manuscripts. This file will be published in conjunction with your paper and will include the anonymous referee reports, your point-by-point response and all pertinent correspondence relating to the manuscript. Let us know whether you agree with the publication of the RPF and as here, if you want to remove or not any figures from it prior to publication. Please note that the Authors checklist will be published at the end of the RPF.
7) Please provide a point-by-point letter INCLUDING my comments as well as the reviewer's reports and your detailed responses (as Word file).

I look forward to reading a new revised version of your manuscript as soon as possible.

Yours sincerely,

Zeljko Durdevic

Zeljko Durdevic
Editor
EMBO Molecular Medicine

*** Instructions to submit your revised manuscript ***

*** PLEASE NOTE *** As part of the EMBO Publications transparent editorial process initiative (see our Editorial at https://www.embopress.org/doi/pdf/10.1002/emmm.201000094), EMBO Molecular Medicine will publish online a Review Process File to accompany accepted manuscripts.

In the event of acceptance, this file will be published in conjunction with your paper and will include the anonymous referee reports, your point-by-point response and all pertinent correspondence relating to the manuscript. If you do NOT want this file to be published, please inform the editorial office at contact@embomolmed.org.

When submitting your revised manuscript, please include:

1) a .docx formatted version of the manuscript text (including Figure legends and tables)

2) Separate figure files*

3) supplemental information as Expanded View and/or Appendix. Please carefully check the authors guidelines for formatting Expanded view and Appendix figures and tables at https://www.embopress.org/page/journal/17574684/authorguide#expandedview

4) a letter INCLUDING the reviewer's reports and your detailed responses to their comments (as Word file).

5) The paper explained: EMBO Molecular Medicine articles are accompanied by a summary of the articles to emphasize the major findings in the paper and their medical implications for the non-specialist reader. Please provide a draft summary of your article highlighting
- the medical issue you are addressing,
- the results obtained and
- their clinical impact.
This may be edited to ensure that readers understand the significance and context of the research.
Please refer to any of our published articles for an example.

6) For more information: There is space at the end of each article to list relevant web links for further consultation by our readers. Could you identify some relevant ones and provide such information as well? Some examples are patient associations, relevant databases, OMIM/proteins/genes links, author's websites, etc...

7) Author contributions: the contribution of every author must be detailed in a separate section.

8) EMBO Molecular Medicine now requires a complete author checklist (https://www.embopress.org/page/journal/17574684/authorguide) to be submitted with all revised manuscripts. Please use the checklist as guideline for the sort of information we need WITHIN the manuscript. The checklist should only be filled with page numbers were the information can be found. This is particularly important for animal reporting, antibody dilutions (missing) and exact values and n that should be indicted instead of a range.

9) Every published paper now includes a 'Synopsis' to further enhance discoverability. Synopses are displayed on the journal webpage and are freely accessible to all readers. They include a short stand first (maximum of 300 characters, including space) as well as 2-5 one sentence bullet points that summarise the paper. Please write the bullet points to summarise the key NEW findings. They should be designed to be complementary to the abstract - i.e. not repeat the same text. We encourage inclusion of key acronyms and quantitative information (maximum of 30 words / bullet point). Please use the passive voice. Please attach these in a separate file or send them by email, we will incorporate them accordingly.

You are also welcome to suggest a striking image or visual abstract to illustrate your article. If you do please provide a jpeg file 550 px-wide x 400-px high.

10) A Conflict of Interest statement should be provided in the main text

11) Please note that we now mandate that all corresponding authors list an ORCID digital identifier. This takes <90 seconds to complete. We encourage all authors to supply an ORCID identifier, which will be linked to their name for unambiguous name identification.

Currently, our records indicate that the ORCID for your account is 0000-0001-5247-3238.

Please click the link below to modify this ORCID:
Link Not Available

12) The system will prompt you to fill in your funding and payment information. This will allow Wiley to send you a quote for the article processing charge (APC) in case of acceptance. This quote takes into account any reduction or fee waivers that you may be eligible for. Authors do not need to pay any fees before their manuscript is accepted and transferred to our publisher.

*Additional important information regarding Figures

Each figure should be given in a separate file and should have the following resolution:
Graphs 800-1,200 DPI
Photos 400-800 DPI
Colour (only CMYK) 300-400 DPI"

Figures are not edited by the production team. All lettering should be the same size and style; figure panels should be indicated by capital letters (A, B, C etc). Gridlines are not allowed except for log plots. Figures should be numbered in the order of their appearance in the text with Arabic numerals. Each Figure must have a separate legend and a caption is needed for each panel.

*Additional important information regarding figures and illustrations can be found at https://bit.ly/EMBOPressFigurePreparationGuideline. See also figure legend preparation guidelines: https://www.embopress.org/page/journal/17574684/authorguide#figureformat

The system will prompt you to fill in your funding and payment information. This will allow Wiley to send you a quote for the article processing charge (APC) in case of acceptance. This quote takes into account any reduction or fee waivers that you may be eligible for. Authors do not need to pay any fees before their manuscript is accepted and transferred to our publisher.

***** Reviewer's comments *****

Referee #1 (Remarks for Author):

I am satisfied

Referee #2 (Comments on Novelty/Model System for Author):

This is a very interesting study carried out with cutting-edge technologies to investigate the metabolic changes in mitochondrial myopathies. The direct medical impact is currently unclear, but it may have potential in the future. The models used are adequate and well-justified.

Referee #2 (Remarks for Author):

The authors made an important effort to clarify their findings and to address my concerns.

1st Revision-Editorial Decision, 3rd May 2023

3rd May 2023

Dear Dr. D'Aurelio,

Thank you for the submission of your revised manuscript to EMBO Molecular Medicine. I am pleased to inform you that we will be able to accept your manuscript pending the following final amendments:

Editor
1) In the main manuscript file, please do the following:
In M&M, please include statement that the informed consent was obtained from all human subjects and that the experiments conformed to the principles set out in the WMA Declaration of Helsinki and the Department of Health and Human Services Belmont Report.

The statement is now included in the Materials and Methods section.

Data availability: Please be aware that all deposited datasets should be made freely available upon acceptance, without restriction. Use the following format to report the accession number of your data:

The datasets produced in this study are available in the following databases:
[data type]: [full name of the resource] [accession number/identifier] ([doi or URL or identifiers.org/DATABASE:ACCESSION])

Information regarding the dataset is available without restriction.

2) Author Checklist: Please check the list carefully and enter all required information. Currently information about human participants are missing.

Human participant information has been added to Materials and Methods and Table S1.

3) Funding: Please place funding information under "Acknowledgment".

The funding information is now underneath the “Acknowledgment” section.

4) Synopsis:
Synopsis text: Please provide a short standfirst (maximum of 300 characters, including space) as well as 2-5 one sentence bullet points that summarise the paper as a .doc file. Please write the bullet points to summarise the key NEW findings. They should be designed to be complementary to the abstract - i.e. not repeat the same text. We encourage inclusion of key acronyms and quantitative information (maximum of 30 words / bullet point). Please use the passive voice.
- Please check your synopsis text and image before submission with your revised manuscript. Please be aware that in the proof stage minor corrections only are allowed (e.g., typos).

5) For more information: Remove information about Appendix. This space should be used to list relevant web links for further consultation by our readers. Could you identify some relevant ones and provide such information as well? Some examples are patient associations, relevant databases, OMIM/proteins/genes links, author's websites, etc...

The appendix was removed from "For more information."

6) As part of the EMBO Publications transparent editorial process initiative (see our Editorial at http://embomolmed.embopress.org/content/2/9/329), EMBO Molecular Medicine will publish online a Review Process File (RPF) to accompany accepted manuscripts. This file will be published in conjunction with your paper and will include the anonymous referee reports, your point-by-point response and all pertinent correspondence relating to the manuscript. Let us know whether you agree with the publication of the RPF and as here, if you want to remove or not any figures from it prior to publication. Please note that the Authors checklist will be published at the end of the RPF.

We agree with the publication of our anonymous referee reports, point-by-point response, and correspondence regarding the manuscript.

I look forward to reading a new revised version of your manuscript as soon as possible.

Yours sincerely,

Zeljko Durdevic

Zeljko Durdevic
Editor
EMBO Molecular Medicine

12th May 2023

Dear Dr. D'Aurelio,

We are pleased to inform you that your manuscript is accepted for publication and is now being sent to our publisher to be included in the next available issue of EMBO Molecular Medicine.

Please read below for additional IMPORTANT information regarding your article, its publication and the production process.

Congratulations on your interesting work,

Zeljko Durdevic

Zeljko Durdevic
Editor
EMBO Molecular Medicine

Follow us on Twitter @EmboMolMed
Sign up for eTOCs at embopress.org/alertsfeeds

*** *** *** IMPORTANT INFORMATION *** *** ***

SPEED OF PUBLICATION
The journal aims for rapid publication of papers, using using the advance online publication "Early View" to expedite the process: A properly copy-edited and formatted version will be published as "Early View" after the proofs have been corrected. Please help the Editors and publisher avoid delays by providing e-mail address(es), telephone and fax numbers at which author(s) can be contacted.

Should you be planning a Press Release on your article, please get in contact with embomolmed@wiley.com as early as possible, in order to coordinate publication and release dates.

LICENSE AND PAYMENT:

All articles published in EMBO Molecular Medicine are fully open access: immediately and freely available to read, download and share.

EMBO Molecular Medicine charges an article processing charge (APC) to cover the publication costs. You, as the corresponding author for this manuscript, should have already received a quote with the article processing fee separately. Please let us know in case this quote has not been received.

Once your article is at Wiley for editorial production you will receive an email from Wiley's Author Services system, which will ask you to log in and will present you with the publication license form for completion. Within the same system the publication fee can be paid by credit card, an invoice, pro forma invoice or purchase order can be requested.

Payment of the publication charge and the signed Open Access Agreement form must be received before the article can be published online.

PROOFS

You will receive the proofs by e-mail approximately 2 weeks after all relevant files have been sent o our Production Office. Please return them within 48 hours and if there should be any problems, please contact the production office at embopressproduction@wiley.com.

Please inform us if there is likely to be any difficulty in reaching you at the above address at that time. Failure to meet our

deadlines may result in a delay of publication.

All further communications concerning your paper proofs should quote reference number EMM-2022-16951-V3 and be directed to the production office at embopressproduction@wiley.com.

Thank you,

Zeljko Durdevic
Editor
EMBO Molecular Medicine

EMBO Press Author Checklist

Corresponding Author Name: Marilena D'Aurelio
Journal Submitted to: EMBO Molecular Medicine
Manuscript Number: EMM-2022-16951

USEFUL LINKS FOR COMPLETING THIS FORM
The EMBO Journal - Author Guidelines
EMBO Reports - Author Guidelines
Molecular Systems Biology - Author Guidelines
EMBO Molecular Medicine - Author Guidelines

Reporting Checklist for Life Science Articles (updated January

This checklist is adapted from Materials Design Analysis Reporting (MDAR) Checklist for Authors. MDAR establishes a minimum set of requirements in transparent reporting in the life sciences (see Statement of Task: 10.31222/osf.io/9sm4x). Please follow the journal's guidelines in preparing your manuscript.

Please note that a copy of this checklist will be published alongside your article.

Abridged guidelines for figures

1. Data

The data shown in figures should satisfy the following conditions:

- the data were obtained and processed according to the field's best practice and are presented to reflect the results of the experiments in an accurate and unbiased manner.
- ideally, figure panels should include only measurements that are directly comparable to each other and obtained with the same assay.
- plots include clearly labeled error bars for independent experiments and sample sizes. Unless justified, error bars should not be shown for technical
- if n<5, the individual data points from each experiment should be plotted. Any statistical test employed should be justified.
- Source Data should be included to report the data underlying figures according to the guidelines set out in the authorship guidelines on Data

2. Captions

Each figure caption should contain the following information, for each panel where they are relevant:

- a specification of the experimental system investigated (eg cell line, species name).
- the assay(s) and method(s) used to carry out the reported observations and measurements.
- an explicit mention of the biological and chemical entity(ies) that are being measured.
- an explicit mention of the biological and chemical entity(ies) that are altered/varied/perturbed in a controlled manner.
- the exact sample size (n) for each experimental group/condition, given as a number, not a range;
- a description of the sample collection allowing the reader to understand whether the samples represent technical or biological replicates (including how many animals, litters, cultures, etc.).
- a statement of how many times the experiment shown was independently replicated in the laboratory.
- definitions of statistical methods and measures:
  - common tests, such as t-test (please specify whether paired vs. unpaired), simple χ2 tests, Wilcoxon and Mann-Whitney tests, can be unambiguously identified by name only, but more complex techniques should be described in the methods section;
  - are tests one-sided or two-sided?
  - are there adjustments for multiple comparisons?
  - exact statistical test results, e.g., P values = x but not P values < x;
  - definition of 'center values' as median or average;
  - definition of error bars as s.d. or s.e.m.

Please complete ALL of the questions below.
Select "Not Applicable" only when the requested information is not relevant for your study.

Materials

Newly Created Materials	Information included in the manuscript?	In which section is the information available? (Reagents and Tools Table, Materials and Methods, Figures, Data Availability Section)
New materials and reagents need to be available; do any restrictions apply?	Not Applicable	

Antibodies	Information included in the manuscript?	In which section is the information available? (Reagents and Tools Table, Materials and Methods, Figures, Data Availability Section)
For **antibodies** provide the following information:<br>- Commercial antibodies: RRID (if possible) or supplier name, catalogue number and or/clone number<br>- Non-commercial: RRID or citation	Yes	Materials and Methods

DNA and RNA sequences	Information included in the manuscript?	In which section is the information available? (Reagents and Tools Table, Materials and Methods, Figures, Data Availability Section)
**Short novel DNA or RNA including primers, probes**: provide the sequences.	Not Applicable	

Cell materials	Information included in the manuscript?	In which section is the information available? (Reagents and Tools Table, Materials and Methods, Figures, Data Availability Section)
**Cell lines:** Provide species information, strain. Provide accession number in repository **OR** supplier name, catalog number, clone number, and/**OR** RRID.	Not Applicable	
**Primary cultures:** Provide species, strain, sex of origin, genetic modification status.	Not Applicable	
Report if the cell lines were recently **authenticated** (e.g., by STR profiling) and tested for mycoplasma contamination.	Not Applicable	

Experimental animals	Information included in the manuscript?	In which section is the information available? (Reagents and Tools Table, Materials and Methods, Figures, Data Availability Section)
**Laboratory animals or Model organisms:** Provide species, strain, sex, age, genetic modification status. Provide accession number in repository **OR** supplier name, catalog number, clone number, **OR** RRID.	Yes	Materials and Methods
**Animal observed in or captured from the field:** Provide species, sex, and age where possible.	Not Applicable	
Please detail **housing and husbandry conditions**.	Yes	Materials and Methods

Plants and microbes	Information included in the manuscript?	In which section is the information available? (Reagents and Tools Table, Materials and Methods, Figures, Data Availability Section)
**Plants:** provide species and strain, ecotype and cultivar where relevant, unique accession number if available, and source (including location for collected wild specimens).	Not Applicable	
**Microbes:** provide species and strain, unique accession number if available, and source.	Not Applicable	

Human research participants	Information included in the manuscript?	In which section is the information available? (Reagents and Tools Table, Materials and Methods, Figures, Data Availability Section)
If collected and within the bounds of privacy constraints report on age, sex and gender or ethnicity for all study participants.	Yes	Table S1; Materials and Methods

Core facilities	Information included in the manuscript?	In which section is the information available? (Reagents and Tools Table, Materials and Methods, Figures, Data Availability Section)
If your work benefited from core facilities, was their service mentioned in the acknowledgments section?	Yes	Acknowledgments

Design

Study protocol	Information included in the manuscript?	In which section is the information available? (Reagents and Tools Table, Materials and Methods, Figures, Data Availability Section)
If study protocol has been **pre-registered, provide DOI in the manuscript**. For clinical trials, provide the trial registration number **OR** cite DOI.	Not Applicable	
Report the **clinical trial registration number** (at ClinicalTrials.gov or equivalent), where applicable.	Not Applicable	

Laboratory protocol	Information included in the manuscript?	In which section is the information available? (Reagents and Tools Table, Materials and Methods, Figures, Data Availability Section)
Provide DOI OR other citation details if **external detailed step-by-step protocols** are available.	Not Applicable	

Experimental study design and statistics	Information included in the manuscript?	In which section is the information available? (Reagents and Tools Table, Materials and Methods, Figures, Data Availability Section)
Include a statement about **sample size** estimate even if no statistical methods were used.	Not Applicable	
Were any steps taken to minimize the effects of subjective bias when allocating animals/samples to treatment (e.g. **randomization procedure**)? If yes, have they been described?	Not Applicable	
Include a statement about **blinding** even if no blinding was done.	Yes	Materials and Methods
Describe **inclusion/exclusion criteria** if samples or animals were excluded from the analysis. Were the criteria pre-established?<br><br>If sample or data points were omitted from analysis, report if this was due to attrition or intentional exclusion and provide justification.	Not Applicable	
For every figure, are **statistical tests** justified as appropriate? Do the data meet the assumptions of the tests (e.g., normal distribution)? Describe any methods used to assess it. Is there an estimate of variation within each group of data? Is the variance similar between the groups that are being statistically compared?	Yes	

Sample definition and in-laboratory replication	Information included in the manuscript?	In which section is the information available? (Reagents and Tools Table, Materials and Methods, Figures, Data Availability Section)
In the figure legends: state number of times the experiment was **replicated** in laboratory.	Yes	Figures
In the figure legends: define whether data describe **technical or biological replicates**.	Yes	Figures

Ethics

Ethics	Information included in the manuscript?	In which section is the information available? (Reagents and Tools Table, Materials and Methods, Figures, Data Availability Section)
Studies involving **human participants**: State details of **authority granting ethics approval** (IRB or equivalent committee(s), provide reference number for approval.	Yes	Materials and Methods
Studies involving **human participants**: Include a statement confirming that **informed consent** was obtained from all subjects and that the experiments conformed to the principles set out in the WMA Declaration of Helsinki and the Department of Health and Human Services Belmont Report.	Yes	Materials and Methods
Studies involving **human participants:** For publication of **patient photos**, include a statement confirming that consent to publish was obtained.	Yes	Materials and Methods
Studies involving experimental **animals**: State details of **authority granting ethics approva**l (IRB or equivalent committee(s), provide reference number for approval. Include a statement of compliance with ethical regulations.	Yes	Materials and Methods
Studies involving **specimen and field samples:** State if relevant **permits** obtained, provide details of authority approving study; if none were required, explain why.	Not Applicable	

Dual Use Research of Concern (DURC)	Information included in the manuscript?	In which section is the information available? (Reagents and Tools Table, Materials and Methods, Figures, Data Availability Section)
Could your study fall under dual use research restrictions? Please check biosecurity documents and list of **select agents and toxins** (CDC): https://www.selectagents.gov/sat/list.htm	Not Applicable	
If you used a select agent, is the security level of the lab appropriate and reported in the manuscript?	Not Applicable	
If a study is subject to dual use research of concern regulations, is the name of the **authority granting approval and reference number** for the regulatory approval provided in the manuscript?	Not Applicable	

Reporting

The MDAR framework recommends adoption of discipline-specific guidelines, established and endorsed through community initiatives. Journals have their own policy about requiring specific guidelines and recommendations to complement MDAR.

Adherence to community standards	Information included in the manuscript?	In which section is the information available? (Reagents and Tools Table, Materials and Methods, Figures, Data Availability Section)
State if relevant guidelines or checklists (e.g., **ICMJE, MIBBI, ARRIVE, PRISMA**) have been followed or provided.	Not Applicable	
For **tumor marker prognostic studies,** we recommend that you follow the **REMARK** reporting guidelines (see link list at top right). See author guidelines, under 'Reporting Guidelines'. Please confirm you have followed these guidelines.	Not Applicable	
For **phase II and III randomized controlled trials**, please refer to the **CONSORT** flow diagram (see link list at top right) and submit the CONSORT checklist (see link list at top right) with your submission. See author guidelines, under 'Reporting Guidelines'. Please confirm you have submitted this list.	Not Applicable	

Data Availability

Data availability	Information included in the manuscript?	In which section is the information available? (Reagents and Tools Table, Materials and Methods, Figures, Data Availability Section)
Have **primary datasets** been deposited according to the journal's guidelines (see 'Data Deposition' section) and the respective accession numbers provided in the Data Availability Section?	Yes	Data Availability Section
Were **human clinical and genomic datasets** deposited in a public access-controlled repository in accordance to ethical obligations to the patients and to the applicable consent agreement?	Not Applicable	
Are **computational models** that are central and integral to a study available without restrictions in a machine-readable form? Were the relevant accession numbers or links provided?	Not Applicable	
If publicly available data were reused, provide the respective **data citations in the reference list**.	Not Applicable	
