# Supplementary material for: A coordinated multiorgan metabolic response contributes to human mitochondrial myopathy
Source: EMBO Mol Med. 2023 May 24;15(7):e16951. doi: 10.15252/emmm.202216951 (PMC10331581; doi:10.15252/emmm.202216951)
Supplement: Supplementary file 2 — Source Data for Appendix [file EMMM-15-e16951-s005.zip › Fig._S2.zip!/Fig. S2/Fig. S2U-V-Y/GOT1 orig.pdf]

Image ID: 0006216_01
Acquire Time: Oct 31, 2017 1:20:28 PM

Acquisition Information

#	Image ID	Acquire Time	Channels	Resolution	Intensities	Quality	Analysis	Image Name	Comment
1	0006216_01	Oct 31, 2017 1:20:28 PM	700 800	169um	Auto Auto	medium	Manual	0006216_01	M109 GOT1 R

Image Display Values

Channel	Color	Minimum	Maximum	K
700	Gray Scale (Black on White)	0.0287	22.0	0

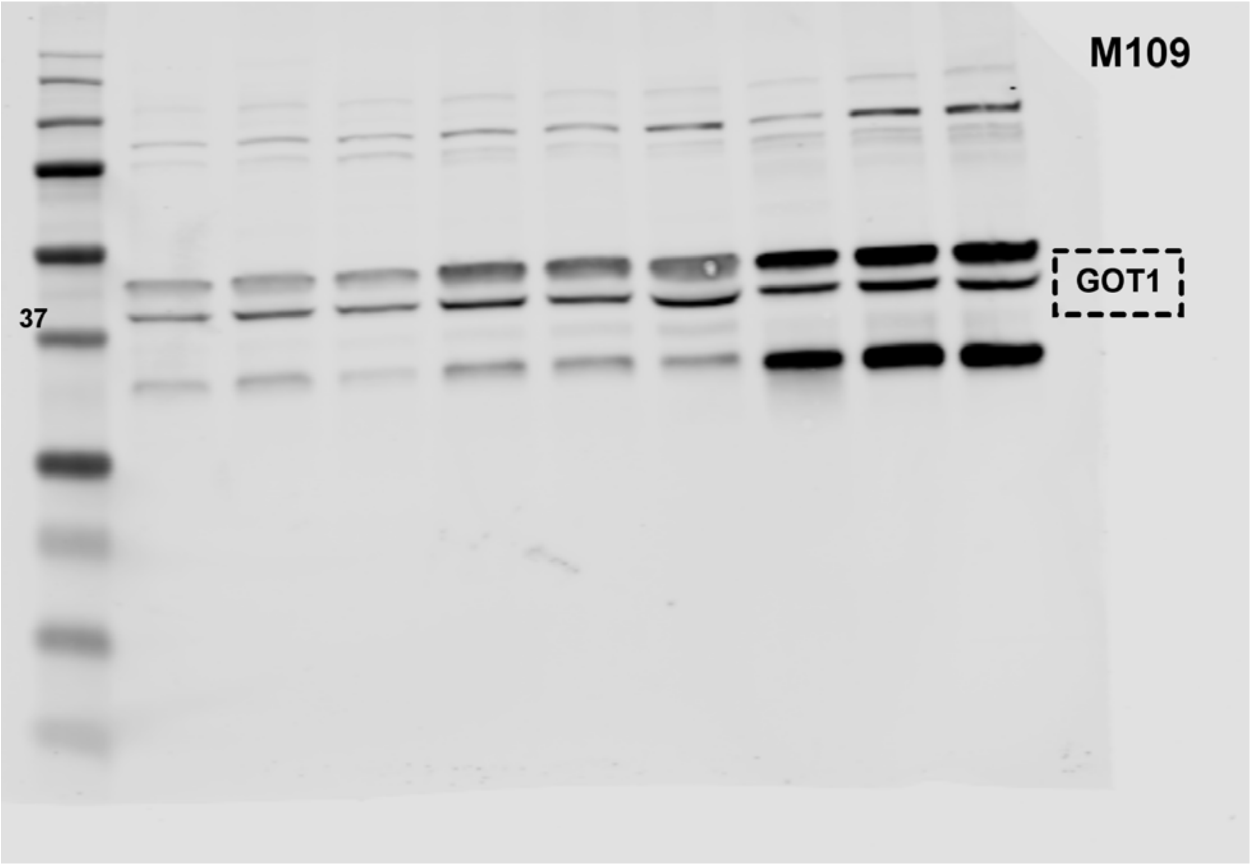

Acquisition Information (continued)

#	Image Modifications	Experiment
1		
