# Supplementary material for: A coordinated multiorgan metabolic response contributes to human mitochondrial myopathy
Source: EMBO Mol Med. 2023 May 24;15(7):e16951. doi: 10.15252/emmm.202216951 (PMC10331581; doi:10.15252/emmm.202216951)

**Image ID 0006528_01**
**April 29, 2019**

Image Display Parameters

| Channel | Color | Minimum | Maximum | K |
|---|---|---|---|---|
| 700 | Gray Scale (Black on White) | 0.00318 | 21.2 | 0 |
| 800 | Gray Scale (Black on White) | 0.00786 | 6.16 | 0 |

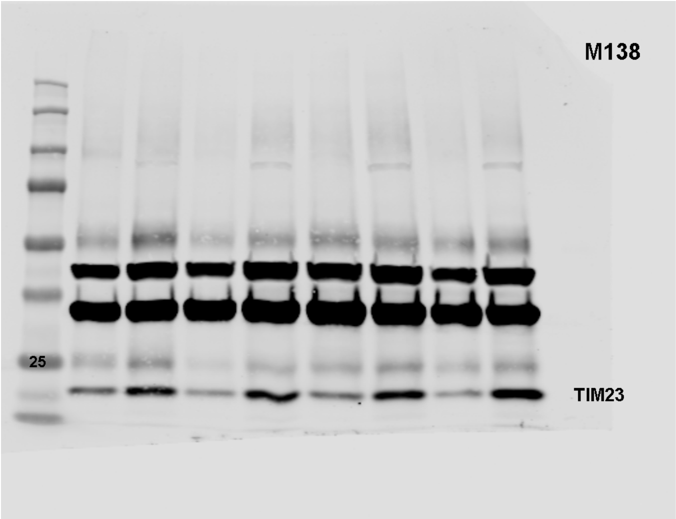

Supplement: Supplementary file 2 — Source Data for Appendix [file EMMM-15-e16951-s005.zip › Fig._S2.zip!/Fig. S2/Fig. S2K-L/TIM23_2019-04-29.pdf]
