# Supplementary material for: A coordinated multiorgan metabolic response contributes to human mitochondrial myopathy
Source: EMBO Mol Med. 2023 May 24;15(7):e16951. doi: 10.15252/emmm.202216951 (PMC10331581; doi:10.15252/emmm.202216951)

Image ID 0006208_01
May 6, 2019

Image Display Parameters

Channel	Color	Minimum	Maximum	K
700	Gray Scale (Black on White)	0.0254	18.5	0

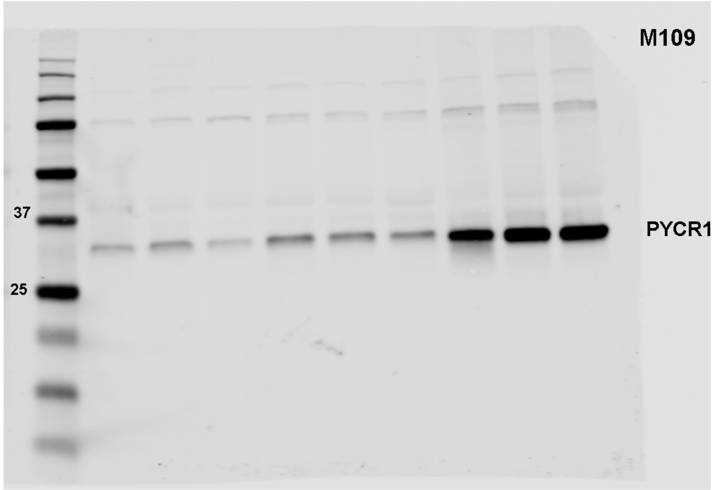

Supplement: Supplementary file 2 — Source Data for Appendix [file EMMM-15-e16951-s005.zip › Fig._S6.zip!/Fig. S6/Fig. S6E-F/PYCR1.pdf]
