# Supplementary material for: A coordinated multiorgan metabolic response contributes to human mitochondrial myopathy
Source: EMBO Mol Med. 2023 May 24;15(7):e16951. doi: 10.15252/emmm.202216951 (PMC10331581; doi:10.15252/emmm.202216951)
Supplement: Supplementary file 2 — Source Data for Appendix [file EMMM-15-e16951-s005.zip › Fig._S4.zip!/Fig. S4/Fig. S4G-H/ATF4.pdf]

Image ID: 0008989_01
Acquire Time: Mar 9, 2023 1:51:17 PM

Acquisition Information

#	Image ID	Acquire Time	Channels	Resolution	Intensities	Quality	Analysis	Image Name
1	0008989_01	Mar 9, 2023 1:51:17 PM	700 800	169um	Auto Auto	medium	Manual	0008989_01

Image Display Values

Channel	Color	Minimum	Maximum	K
800	Gray Scale (Black on White)	0.00476	1.56	0

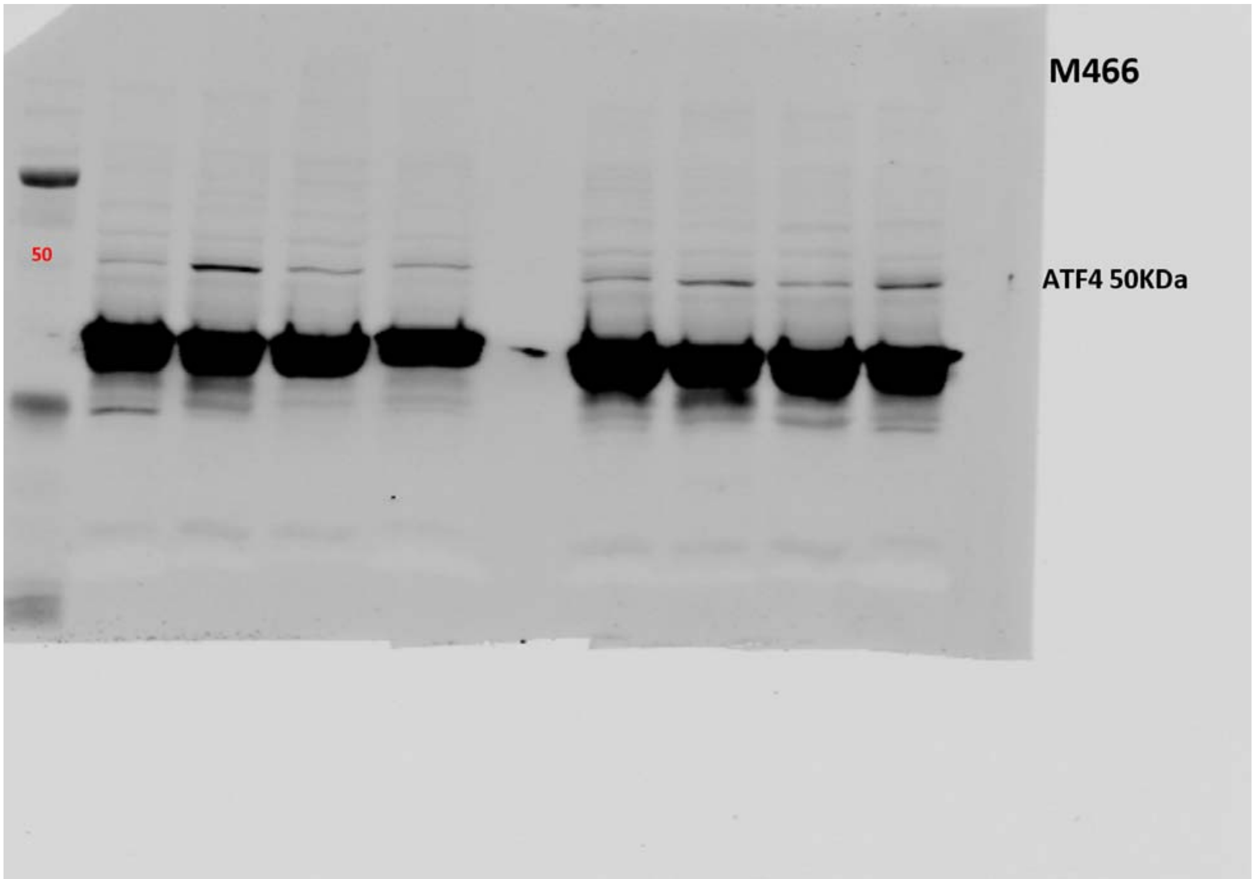

Acquisition Information (continued)

#	Comment	Image Modifications	Experiment
1	M466 Human Muscle Samples ATF4 M (1:500)		
